# Aligning machine and human visual representations across abstraction levels

**DOI:** 10.1038/s41586-025-09631-6

**Published:** 2025-11-12

**Authors:** Lukas Muttenthaler, Klaus Greff, Frieda Born, Bernhard Spitzer, Simon Kornblith, Michael C. Mozer, Klaus-Robert Müller, Thomas Unterthiner, Andrew K. Lampinen

**Affiliations:** 1 Google DeepMind, Berlin, Germany; 2 Machine Learning Group, Technische Universität Berlin, Berlin, Germany; 3 BIFOLD, Berlin Institute for the Foundations of Learning and Data, Berlin, Germany; 4 Max Planck Institute for Human Cognitive and Brain Sciences, Leipzig, Germany; 5 Max Planck Institute for Human Development, Berlin, Germany; 6 TUD Dresden University of Technology, Dresden, Germany; 7 Anthropic, San Francisco, CA USA; 8 GoogleDeepMind, San Francisco, CA USA; 9 Department of Artificial Intelligence, Korea University, Seoul, South Korea; 10 Max Planck Institute for Informatics, Saarbrücken, Germany

**Keywords:** Computational science, Human behaviour

## Abstract

Deep neural networks have achieved success across a wide range of applications, including as models of human behaviour and neural representations in vision tasks^1,2^. However, neural network training and human learning differ in fundamental ways, and neural networks often fail to generalize as robustly as humans do^3,4^, raising questions regarding the similarity of their underlying representations. We need to determine what is missing for modern learning systems to exhibit more human-aligned behaviour. Here we highlight a key misalignment between vision models and humans: whereas human conceptual knowledge is hierarchically organized from fine- to coarse-scale distinctions (for example, ref. ^5^), model representations do not accurately capture all these levels of abstraction. To address this misalignment, we first train a teacher model to imitate human judgements, then transfer human-aligned structure from its representations to refine the representations of pretrained state-of-the-art vision foundation models via fine-tuning. These human-aligned models more accurately approximate human behaviour and uncertainty across a wide range of similarity tasks, including a dataset of human judgements spanning multiple levels of semantic abstractions. They also perform better on a diverse set of machine learning tasks, increasing generalization and out-of-distribution robustness. Thus, infusing neural networks with additional human knowledge yields a best-of-both-worlds representation that is both more consistent with human cognitive judgements and more practically useful, paving the way towards more robust, interpretable and human-aligned artificial intelligence systems.

## Abstract (web summary)

Aligning foundation models with human judgments enables them to more accurately approximate human behaviour and uncertainty across various levels of visual abstraction, while additionally improving their generalization performance.

## Main

Although deep learning has recently driven rapid progress in areas of artificial intelligence such as natural language processing^6^ and computer vision^7–9^, even the best of these systems often fail in ways that humans would not^4,10–13^. These failures have led to renewals^3,12^ of older arguments^14,15^ that neural networks lack the essential ingredients of human intelligence. Therefore, we need to determine how can we build systems that produce more human-aligned behaviour.

Human perception is robust and generalizes across different visual settings^4,16,17^. However, model performance declines—often markedly—if the data distribution shifts between the training and test sets (for example, refs. ^11,18^). This lack of robustness in vision model representations poses a challenge for downstream applications that require generalization (for example, refs. ^10,11,19^). In addition, humans tend to be well calibrated—for example, when they are asked to judge visual similarity^17^—that is, humans’ (un)certainty tends to correlate with their (in)accuracy. Artificial intelligence systems, however, are often overconfident and show high certainty even when their predictions are incorrect^20^. Thus, many differences remain to be reconciled before we can ultimately achieve human-like artificial intelligence.

Here we highlight a key misalignment between humans and deep learning models that may underlie some of these differences: model representations tend to fail to capture the full multi-level conceptual structure of human knowledge. Although model representations successfully encode the local human-perceived similarity structure among closely related entities (for example, different dog breeds), the global relationships between concepts with more abstract semantic relations (for example, dogs and fish, which are both animate but visually dissimilar) are modelled much less systematically. Human neural representations, however, are organized by global features such as animacy^5,21^, and at multiple finer scales that capture nuanced semantic relationships^21–24^. This lack of global organization in the representations of deep learning models across levels of the conceptual hierarchy likely contributes to the aforementioned weaknesses of these models.

A challenge for addressing this misalignment is that collecting representative datasets of human judgements is challenging and expensive. We therefore propose a method for synthesizing simulated (approximately) human-aligned similarity judgements via a surrogate teacher model—a large foundation model that we align using an affine transformation^25^ and uncertainty distillation on a small existing dataset^26^. We use this surrogate to produce the AligNet dataset—human-aligned pseudolabels (compare ref. ^27^) from the surrogate model for triplets sampled from ImageNet^28^ using a clustering-based data-grouping method. We fine-tune various vision foundation models on AligNet using a similarity-space distillation objective. These models show substantially more human-aligned predictions on various cognitive science tasks—including Levels, a dataset of human semantic judgements reflecting multiple levels of abstraction. Furthermore, these aligned models show improved accuracy and out-of-distribution robustness across many downstream machine learning tasks, thus showing the improved generalizability of the aligned model representations. We release our aligned models and training and evaluation datasets.

In summary, our work contributes to better understanding a key difference between artificial and natural intelligence. Moreover, our results illustrate a principle for aligning models to humans—focusing on the multi-scale relational structure of human knowledge—that may contribute to the more general problem of achieving human-aligned artificial intelligence.

**Fig. 1:**
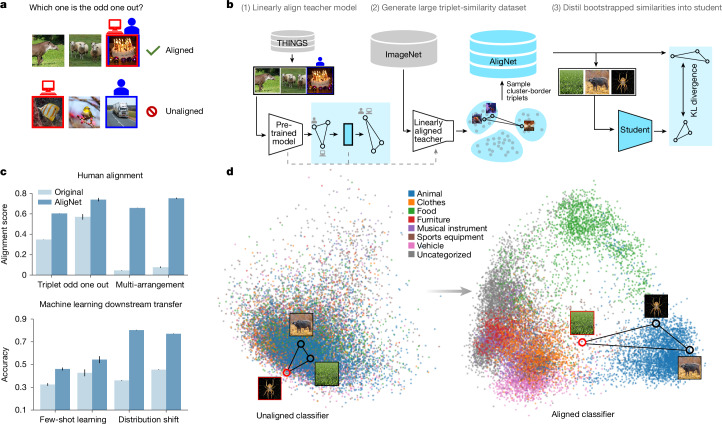
Overview. **a**, An example of the triplet odd-one-out task where a human and a neural network model choose the same (top) and a different (bottom) odd-one-out image, respectively. **b**, The different parts of the AligNet framework depicted from end to end. First, we develop a teacher model of human judgements using the THINGS dataset. Second, we apply this model to ImageNet and cluster its latent representations into semantically meaningful categories. This allows us to generate arbitrarily many similarity judgements. Third, the obtained human-aligned similarity structure information is distilled into a student vision foundation model using a loss function. KL divergence, Kullback–Leibler divergence. **c**, Representative human alignment (top) and machine learning downstream (bottom) results show significant performance improvements of the aligned over the non-aligned version of the ViT-B classifier (up to 123% of relative improvement for ML downstream transfer). Error bars are 95% confidence intervals (CIs). For the human alignment results, we ran 100 bootstraps (repetitions) on the item level where we computed model performance for a single bootstrap using a subset of 1,000 randomly (with replacement) selected items from the respective dataset. Triplet odd-one-out datasets are THINGS and coarse-grained Levels respectively. The multi-arrangement panel represents the results for the ViT-B classifier from Fig. 2c. For machine learning downstream results, we computed the error bars using the binomial proportion CI. We used a normal approximation (‘Wald interval’) to compute the binomial proportion 95% CI which can be calculated using the number of data points in a dataset and the observed prediction performance (here, accuracy) of a model for that dataset. Few-shot learning datasets are Flowers (*n *= 6,149) and UC Merced (*n* = 1,050). Distribution shift datasets are Entity-13 (*n* = 167,592) and Entity-30 (*n* = 153,565) from the BREEDS benchmarks. **d**, Two-dimensional latent space projection for visualizing the change in the representations after alignment. Although the representations of a standard ViT-B classifier model are unstructured and categories overlap, after alignment the representations are grouped into meaningful categories. All photos are taken from Pixabay and are under a Creative Commons licence CC BY 0.

To build foundation models with more human-aligned behaviour, we inject additional supervision about human behaviour into the model representations, using a surrogate teacher model: a vision foundation model whose representations are linearly transformed to approximate human judgements and uncertainty on the THINGS dataset^26^. We use clusters from this teacher model’s representations to sample triplets from ImageNet^28^ and soft-label them using distances in the teachers representation space, then distil these soft labels into the student representations via a Kullback–Leibler divergence objective (Fig. 1). For details, see Methods.

## Towards more human-aligned models

### THINGS triplet odd one out

We first validate that our teacher model performs well on the test data for the THINGS dataset^26^ used in training; as expected, the teacher achieves high performance (61.7% accuracy, close to the human noise ceiling of 66.67%). We then align a variety of student models—trained with objectives ranging from image captioning to classification or self-supervised learning—using this teacher’s representations; all models show substantially improved human alignment on the THINGS tests (relative performance increases from 21.28–74.47%).

### Other cognitive tasks

**Fig. 2:**
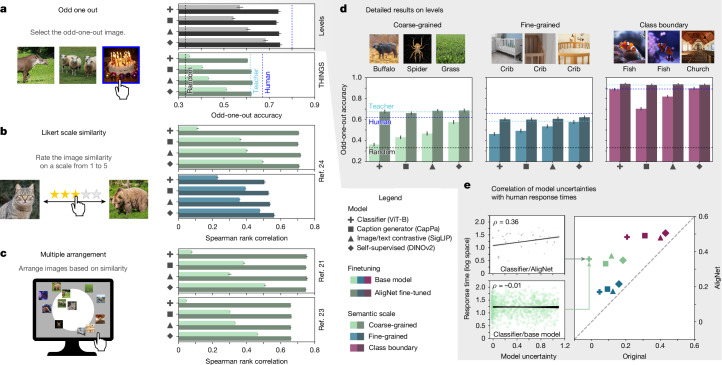
Human alignment results. **a**, Odd-one-out accuracies on the THINGS dataset and performance averaged across all three levels of abstraction for Levels. **b**, Spearman rank correlations for the human-response datasets from ref. ^24^ for the coarse-grained various category and averaged across all fine-grained single-domain categories. **c**, Spearman rank correlations for the multi-arrangement datasets from refs. ^21^,^23^. **d**, Odd-one-out accuracies on our datasets shown individually for the three levels of abstraction. **e**, Spearman rank correlation of model uncertainties and human response times. Model uncertainties are modelled as discrete Shannon entropy of the pairwise similarities in a triplet. All error bars are 95% confidence intervals obtained by bootstrapping. For each dataset and model, we ran 100 bootstraps (repetitions) on the item level where we computed model performance for a single bootstrap using a subset of 1,000 randomly (with replacement) selected items from the respective dataset. All photos are taken from Pixabay and are under a Creative Commons licence CC BY 0.

Our findings generalize across various object similarity tasks that are commonly used in the cognitive sciences—triplet odd-one-out task (relative performance increases up to 73.35%; Fig 2a), Likert scale similarity ratings (up to 6.3-fold increase in Spearman rank correlation coefficient; Fig 2b) and multiple-arrangement tasks (up to 14.47-fold increase in Spearman rank correlation coefficient; Fig. 2c). All performance increases are statistically significant at *α* = 0.05; for details, see Supplementary Information.

## Alignment at multiple levels of abstraction

### Levels dataset

Because previous cognitive datasets were not specifically targeted for assessing knowledge of vision foundation models across levels of abstraction, we collected a dataset of human judgements—which we call Levels—that is based on the triplet odd-one-out task, but stratified across different levels of the semantic hierarchy. Specifically, we collect global coarse-grained semantic, which requires deciding on the odd one out among broadly different categories; local fine-grained semantic, involving discerning subtle distinctions within the same category; and class boundary, testing the capacity to identify category boundaries. For details, see Methods.

### Alignment at multiple levels

The Levels dataset allows us to systematically study discrepancies between human and model decisions across these different levels. We find that our soft-alignment method reduces these discrepancies at all levels, but especially for the global coarse-grained judgements, as we predicted.

### Global coarse-grained

This level shows the largest improvements. The base models achieved low accuracies of 36.09% (classifier ViT-B (ref. ^8^)) to 57.38% (self-supervised DINOv2 (ref. ^29^)). AligNet models significantly improved; all models performed well, with accuracies of 65.70% (ViT-B) to 68.56% (DINOv2)—above the human-to-human reliability score of 61.92% (Fig. 2d, leftmost column). That is, the AligNet models’ responses were more similar to average human responses (as each triplet response is the majority response of the participants) than the level of agreement among the human participants. AligNet models’ relative improvements ranged from 19.48% (DINOv2) to 93.51% (ViT-L).

### Local fine-grained

Most base models did not strongly align to human responses for fine-grained semantics either; all models achieved poor alignment scores of 46.04% (ViT-B) to 57.72% (DINOv2), except for DINOv1, which performed significantly better (62.92%; near the human noise ceiling of 65.92%; Supplementary Table 3). AligNet models achieved increased accuracies of 58.93 (ViT-S) to 62.92% (DINOv1), with relative improvements ranging from 7.84% (DINOv2) to 46.03% (ViT-L) (Fig. 2d, middle column).

### Class boundary

Supervised classifiers and image/text contrastive base models performed close to the noise ceiling; accuracies ranged from 81.96% (SigLIP) to 93.67% (ViT-L). Others performed worse; the CapPa (ref. ^30^) captioning model achieved 70.37%. AligNet fine-tuning brought all models to a similar level, achieving accuracies up to 93.24% (ViT-L), higher than the human noise ceiling of 89.21% (Fig. 2d, rightmost column). Relative improvements ranged from 0.62% (ViT-L) to 32.29% (CapPa). For more performance details, see Supplementary Table 3.

### AligNet model uncertainties correspond to human latencies

We also collected (continuous) human response times, which we use as a proxy of the participants’ uncertainty^31,32^. We measured model uncertainty as the entropy of the three pairwise similarities within each triplet. Base model uncertainties were not correlated with human response times for the coarse (*ρ* = −0.014–0.184) and fine-grained (*ρ* = 0.047–0.160) settings and moderately correlated for the class-boundary setting (*ρ* = 0.208–0.432). All AligNet models showed substantially increased uncertainty alignment across all levels (Fig. 2e), especially the coarse-grained abstraction level (*ρ* = 0.479–0.506).

### Evaluating other model classes

To confirm that the aforementioned representational weaknesses are present in other classes of deep learning models, we evaluated two state-of-the-art natively multimodal large language models, Gemini 2.0 Flash and Gemini 2.5 pro^33^, on Levels. These visual-language models perform similarly to—or slightly better than—the better pretrained vision models across all levels; however, they still substantially underperform our AligNet fine-tuned models (Supplementary Information section 1.7). In addition, we evaluated models trained on Ecoset^34^, an ecologically motivated natural image dataset, and find that Ecoset models have severe difficulties matching the human similarity judgements (Supplementary Information section 1.6). These results confirm that the AligNet fine-tuning offers greater improvements in human alignment than merely incorporating language modelling or ecological training.

## Aligned models reflect the conceptual hierarchy

**Fig. 3:**
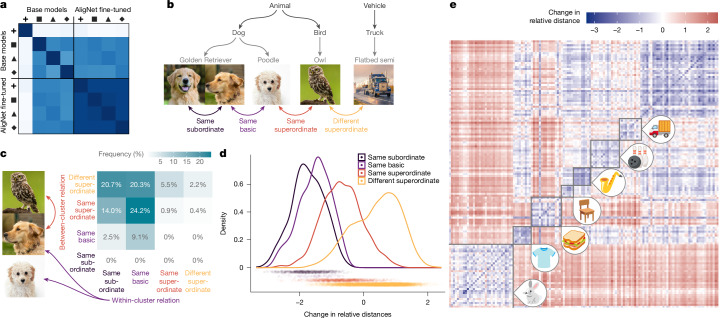
Aligned models reflect the semantic hierarchy. **a**, Before alignment, models trained with different losses have dissimilar representation structures—particularly those trained for supervised classification. After alignment, however, model representation structures are much more similar to each other. **b**, To understand the alignment, we study how the models’ representations change across the semantic hierarchy, from relations between images within the same subordinate category to relations across superordinate categories. **c**, The cluster-driven triplet sampling tends to produce triplets where two images have a closer relation than the third. **d**, The result is that the relations between image representations change following the semantic hierarchy—images from the same subordinate, basic or superordinate category tend to move closer together, whereas those from different superordinate categories move farther apart. Effects are highly statistically significant: *t* > 3.93, *P* < 0.001. **e**, Visualizing the distance changes in more detail, with some superordinate categories boxed on the diagonal and labelled with icons. Panels **d** and **e** are for the representations of ViT-B. Icons in **e**: Copyright 2021 Google Inc. All Rights Reserved.

Next we consider how model representations change after soft alignment. In Fig. 3, we show that although the model representations are dissimilar before alignment, they become more aligned with each other after soft alignment. This convergence is driven by models aligning better with the human conceptual hierarchy (Fig. 3b; compare ref. ^35^). Our soft-alignment procedure embeds this global structure at two levels: first in the cluster-based sampling (Fig. 3c) and then in the soft labels (see above). Because of these factors, the relationships between image representations change during alignment according to their semantic relationship; representations of images from the same basic category tend to move closer together, those of images from the same superordinate category tend to move somewhat closer, and those from different superordinate categories tend to move apart (all effects are highly significant; *t* > 3.93, *P* < 0.001).

As an illustrative example, in a base ViT-B, the representations of lizards are close to those of some plants and fruits owing their similarity in texture, colour or background; after alignment, they become similar to representations of other animals and more distant from those of other, unrelated superordinate categories. This reorganization yields better generalization, for example, when a lizard image is used as an example depicting an abstract category such as animals.

**Extended Data Fig. 2:**
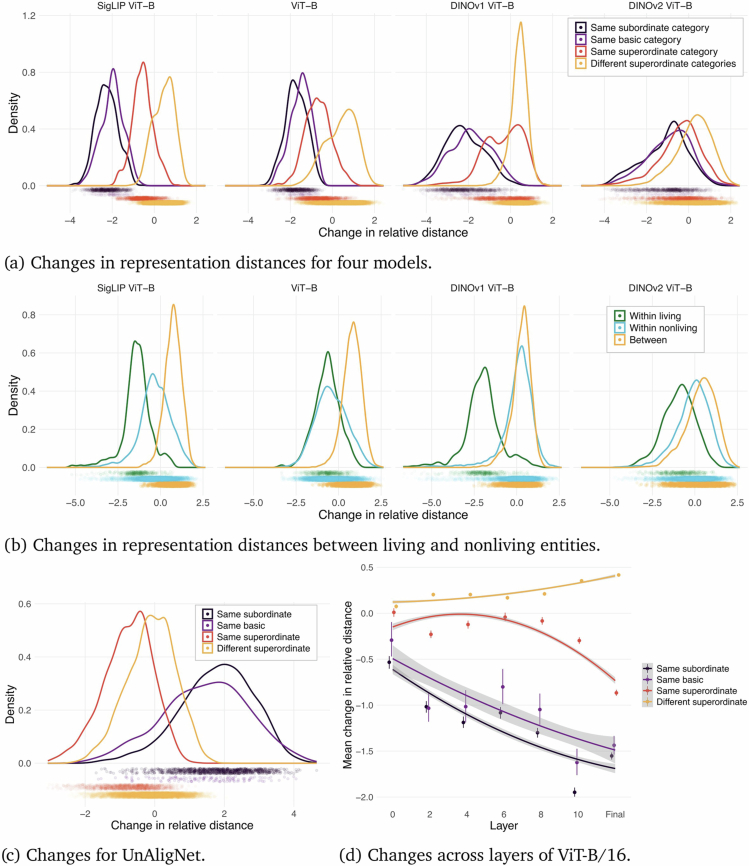
Changes in relative distances between stimuli reflect the superordinate category structure in AligNet models—including at earlier layers and at higher levels of the hierarchy— but not in UnAligNet models. (a) Representation reorganization is similar across four AligNet models; representations move closer or farther depending on their degree of relatedness in the semantic hierarchy. (a) This reorganization also occurs for the animacy distinction in all four models. (c) By contrast, an UnAlignet ablation model shows reorganization at the basic and subordinate levels that is the opposite of what would be desired; viz. these closely-related categories are moving farther apart, with changes that are larger in magnitude than the changes in the superordinate categories. (d) Changes across the layers of ViT-B/16. Although we only optimize representations at the final representation layer, we see directionally consistent reorganization across all layers, with the magnitude of the changes increasing at higher layers. (Errorbars are bootstrap 95%-CIs. Curves are quadratic regression fits to illustrate trends).

**Extended Data Fig. 3:**
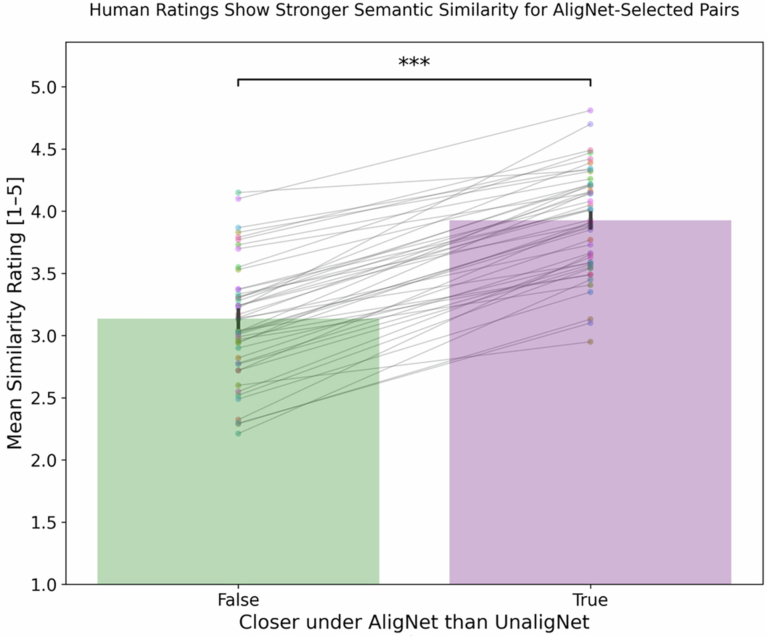
Where AligNet and UnAlignet disagree, AligNet focuses on semantics while UnAligNet relies on more superficial appearance—resulting in humans overwhelmingly favoring AligNet. Human ratings of semantic similarity on image pairs on which AligNet and UnaligNet maximally disagree. The human participants consistently rate the pairs that AligNet represents more similarly as in fact more semantically similar.

Supplementary Information section 3.2 and Extended Data Fig. 2 present more detailed analyses corroborating these results, including reorganization at higher levels such as living versus non-living, across model layers, and in other models and ablations. Furthermore, Supplementary Information section 3.3 and Extended Data Fig. 3 show that where the AligNet model and a baseline unaligned model disagree, human judgements are strongly correlated with those of AligNet, but not the ablation model (which relies more on superficial stylistic features)—in fact, every human participant in the study agreed more with AligNet.

## Alignment improves generalization and robustness

Next we considered how human-aligned representations affect the performance on machine learning tasks. We investigated how alignment improves generalization and out-of-distribution robustness across a variety of downstream tasks.

### One-shot classification

**Fig. 4:**
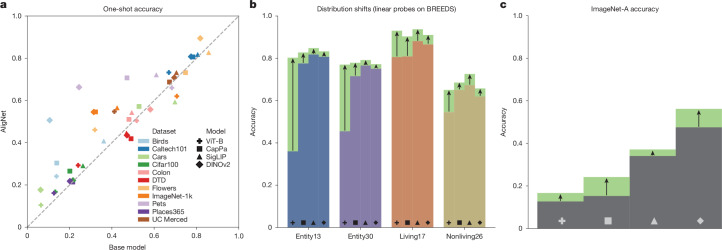
Machine learning downstream results. We evaluated how AligNet fine-tuning affected the downstream task performances of various pretrained vision foundation models using linear probing. **a**, One-shot accuracy before (*x*-axis) versus after (*y*-axis) AligNet fine-tuning on various image datasets, including the Describable Textures Dataset (DTD) and the UC Merced land use dataset. **b**, Accuracy improvements on the four BREEDS distribution shift benchmark datasets. **c**, Accuracy improvements on the ImageNet-A dataset that is used to evaluate model robustness.

We first test an extremely challenging generalization setting: classifying images given only a single labelled example per class. In Fig. 4, we show one-shot performance before and after soft alignment on ten image-classification datasets from varied domains, such as fine-grained bird (Birds^36^) and flower (Flowers^37^) types classification, multi-domain natural image classification (ImageNet^38^) and scene recognition (Places365^39^). The majority of cases (32 of 40) show an improvement, sometimes by a substantial margin (for example, DINOv2 shows a 2.7-fold increase on the Pets dataset); overall, alignment significantly increases the generalization performance on these tasks (*P* < 0.05). These results show how human-aligned representations support strong generalization from little data. See Supplementary Information section 2.1 for additional results, including the complementary benefits of combining our method with other approaches to few-shot generalization.

### Distribution shift

A long-standing problem for applying machine learning algorithms is distribution shift (compare refs. ^40,41^): in deployment, data often differ in subtle ways from the training data, leading to unexpected model failures. To evaluate whether the global structure induced by alignment helps ameliorate this issue, we evaluated our models on the BREEDS benchmarks^42^—which specifically tests generalization under input distribution shifts, using datasets where training and test data points are sampled from different subpopulations. Figure 4b shows that AligNet fine-tuning consistently improves performance significantly across all benchmarks and models types (especially the image classifier, ViT-B). See Supplementary Information section 2.3 for further results.

### Model robustness

Alignment also improves robustness. We evaluate on ImageNet-A^43^, a challenging dataset of natural images that are adversarial (that is, models tend to misclassify them, but humans perform better). Again, alignment improves accuracy for all models (compare Fig. 4c), with improvements of up to 9.5 percentage points (1.6-fold improvement for CapPa). Although our method is not designed for improving out-of-distribution robustness, its improvements are comparable to state-of-the-art methods designed for precisely this problem (Supplementary Table 6).

Together, these machine learning results corroborate that AligNet fine-tuning improves the generalization, transfer and robustness of model representations.

## Discussion

The differences between natural intelligence and the capabilities of neural networks are the subject of long-standing debates^3,14^. Despite the recent progress in artificial intelligence, these discussions persist, because deep learning systems still seem to fail in non-human-like ways^10,11^.

Here, we have highlighted—and addressed—a key deficiency in a broad class of vision foundation models: their representations do not adequately represent the multi-level conceptual structure of human semantic knowledge (‘Towards more human-aligned models’ section). We demonstrate this deficiency through Levels, a dataset of human similarity judgements across multiple levels of abstraction. To address this deficiency, we established a methodological framework for aligning deep learning models’ representations with human similarity judgements to create more human-aligned systems. This framework involves bootstrapping from a small quantity of human data to train a surrogate teacher model, and using this teacher to create a large synthetic dataset (AligNet), which we use to fine-tune various vision foundation models to inject human-aligned structure.

This approach yields significantly increased alignment to human judgements on cognitive science tasks (‘Towards more human-aligned models’ section), and better generalization and robustness on representative machine learning tasks (‘Alignment improves generalization robustness’ section). Thus, soft alignment helps to reduce the brittleness of machine learning models under changing environments. Moreover, our results illustrate how the broader paradigm of studying representational alignment^44,45^ can not only yield insights about how different systems relate but also be leveraged to actively align model representations with human knowledge to improve the models’ generalization abilities.

These results contribute to long-standing debates over which features of human intelligence neural networks may lack^3,14,15^. In particular, one line of critique argues that neural networks lack the capability to appropriately represent abstract relations such as same and different^46,47^, or to organize knowledge into hierarchies of concepts^15^. Although aspects of these critiques have been refuted in simple synthetic settings (for example, ref. ^48^), similar criticisms persist for modern foundation models^12^. Our results show that, although standard training objectives do not adequately capture hierarchical category relations, these relations can be distilled into the models—which improves the models’ resilience under the distribution shifts highlighted in previous critiques. These results show that hierarchical representations may emerge from a system that is neither explicitly hierarchically structured nor trained explicitly on the hierarchy.

Although we focused on vision, similar global misalignments probably arise in other areas of research. For instance, in natural language processing, models are similarly trained with objectives that focus on distinguishing between close matches (for example, prediction objectives that primarily distinguish words that are likely to occur, rather than considering their relations to less probable concepts). Applying alignment techniques may therefore analogously help to better capture the global structure of semantic and syntactic relationships among language inputs that these objectives might miss.

More broadly, artificial intelligence systems have been successfully adopted in many areas. However, these deployments lead to practical^49^ and conceptual^50^ concerns about trustworthiness and safety. It is therefore increasingly important to identify the reasons why these systems occasionally fail and how to alleviate these failures. Our work advances the understanding of the deficiencies of vision model representations, and simultaneously shows a viable path for ameliorating these deficiencies by alignment with human judgements.

Our work has a number of limitations that could be addressed in future efforts. First, the models we used account for neither context in similarity judgements nor higher-order relations. Second, human representations may vary systematically across individuals, cultures and so on. Finally, human judgement is full of flaws, intrinsic contradictions and discrepancies. Given these issues, perfect alignment to human performance may not always be desirable for a technical system. Thus, future work could explore how to best learn from human knowledge without imitating human imperfections.

In summary, we have provided an initial approach to distil global, human-aligned similarity structures into the representations of modern deep neural networks. We have demonstrated an efficient path towards a best-of-both-worlds representation that is both more consistent with human judgements and more practically useful, paving the way towards more robust, interpretable and human-aligned artificial intelligence systems. We hope that our work will inspire more general approaches for aligning foundation models by distiling human priors into their representations.

## Methods

### Soft alignment

This section is organized as follows. We start by describing how we transform model representations into a space that matches human similarity judgements about coarse-grained semantic object relations. We introduce an affine transformation that matches human similarity judgements and injects the uncertainties that humans assign to their triplet odd-one-out choices into a model’s representation space creating a surrogate teacher model. Using the teacher model’s human-aligned representations, we sample triplets of ImageNet^38^ images differently than uniform random sampling by clustering the representations into superordinate categories and using those clusters for data partitioning. We pseudo-label these triplets with human-aligned judgement distributions from the surrogate teacher model. Finally, after having created AligNet triplets, we fine-tune student models with a triplet loss object function.

#### Representational alignment

##### Data

To increase the degree of alignment between human and neural network similarity spaces, we begin from the publicly available THINGS dataset, which is a large behavioural dataset of 4.7 million unique triplet responses from 12,340 human participants for *m* = 1,854 natural object images^51^ from the public THINGS object concept and image database^26^. The THINGS dataset can formally be defined as $D{(\{{a}_{s},{b}_{s}\}|\{{i}_{s},{j}_{s}\,,{k}_{s}\})}_{s=1}^{n}$, which denotes a dataset of *n* object triplets and corresponding human odd-one-out responses, where $\{{a}_{s},{b}_{s}\}\subset \{{i}_{s},{j}_{s}\,,\,{k}_{s}\}$ and $\{{a}_{s},{b}_{s}\}$ is the object pair that was chosen by a human participant among the *s-*th triplet to have the highest similarity. Let ${\bf{X}}\in \,{{\mathbb{R}}}^{m\times p}$ be the teacher model representations for the *m* = 1,854 objects in the THINGS dataset, where *p* is the dimension of the image-representation vector. It is noted that each category in the THINGS dataset is represented by one object image. From **X** we can construct a similarity matrix for all object pairs ${\bf{S}}:= {\bf{X}}\,{{\bf{X}}}^{{\rm{\top }}}\in {{\mathbb{R}}}^{m\times m}$, where ${S}_{i,j}={{\bf{x}}}_{i}^{{\rm{\top }}}{{\bf{x}}}_{j}$ is the representational similarity for objects *i* and *j*, ${\rm{\top }}$ denotes the matrix transpose, and **x**_*i*_ refers to the *i-*th column of **X**.

##### Odd-one-out accuracy

The triplet odd-one-out task is frequently used in the cognitive sciences to measure human notions of object similarity^52–55^. To measure the degree of alignment between human and neural network similarity judgements in the THINGS triplet task, we embed the *m* = 1,854 THINGS images into the representation space of a neural network with ${\bf{X}}\in {{\mathbb{R}}}^{m\times p}$. Given vector representations **x**_1_, ***x***_2_ and **x**_3_ of the 3 images in a triplet, we first construct a similarity matrix ${\bf{S}}\in {{\mathbb{R}}}^{3\times 3}$ where ${S}_{i,j}\,:= \,{{\bf{x}}}_{i}^{{\rm{\top }}}{{\bf{x}}}_{j}$ is the dot product between a pair of image representations. We identify the closest pair of images in the triplet as ${{\rm{a}}{\rm{r}}{\rm{g}}\,{\rm{m}}{\rm{a}}{\rm{x}}}_{i,j > i}{S}_{i,j}$ with the remaining image being the odd one out. We define odd-one-out accuracy as the fraction of triplets where the odd one out ‘chosen by a model’ is identical to the human odd-one-out choice. Thus, our goal is to learn an affine transformation into the THINGS human object similarity space of the form: ${{\bf{x}}}^{^{\prime} }={\bf{W}}{\bf{x}}+{\bf{b}}$. Here, ${\bf{W}}\in {{\mathbb{R}}}^{p\times p}$ is a learned transformation matrix, ${\bf{b}}\in {{\mathbb{R}}}^{p}$ is a bias and ${\bf{x}}\in {{\mathbb{R}}}^{p}$ is the neural network representation for a single object image in the THINGS dataset. We learn the affine transformation for the representation of the image encoder space of the teacher model (see the ‘Surrogate teacher model’ section for details about the teacher model). Using this affine transformation, an entry in the pairwise similarity matrix **S**′—which represents the similarity between two object images *i* and *j*—can now be written as ${S}_{i,j}^{^{\prime} }\,:= \,{({\bf{W}}{{\bf{x}}}_{i}+{\bf{b}})}^{{\rm{\top }}}({\bf{W}}{{\bf{x}}}_{j}+{\bf{b}})$.

##### Hard-alignment loss

Given a similarity matrix of neural network representations **S** and a triplet {*i*, *j*, *k*}, the likelihood of a particular pair, $\{a,b\}\subset \{i,j,k\}$, being most similar to the remaining object being the odd one out, is modelled by the softmax of the object similarities,

1 $$\sigma ({\bf{S}},\tau ):= \exp ({S}_{a,b}/\tau )/\exp ({S}_{i,j}/\tau )+\exp ({S}_{i,k}/\tau )+\exp ({S}_{j,k}/\tau )$$

We can then define the probability of the neural network model to choose the most similar pair (according to the human participants) to be $q(\{a,b\}|\{i,j,k\},{\bf{S}}):= \sigma ({\bf{S}},\tau )$ with a temperature parameter *τ* = 1. For *n* triplet responses, the discrete negative log-likelihood is defined as follows

$${L}_{{\rm{h}}{\rm{a}}{\rm{r}}{\rm{d}}\mbox{-}{\rm{a}}{\rm{l}}{\rm{i}}{\rm{g}}{\rm{n}}}({{\bf{S}}}^{^{\prime} })\,:= \,-\frac{1}{n}\mathop{\sum }\limits_{s=1}^{n}\mathrm{log}\,q(\{{a}_{{\rm{s}}},{b}_{{\rm{s}}}\}|\{{i}_{{\rm{s}}},\,{j}_{{\rm{s}}},\,{k}_{{\rm{s}}}\},\,{{\bf{S}}}^{^{\prime} })$$

##### Modelling human uncertainties

As each triplet response is a discrete choice, we do not have direct access to the uncertainties of a human participant over the objects in a triplet. Thus, the above loss function optimizes a transform to match the human choice but does not take into account the uncertainties over the three odd-one-out alternatives. However, it is possible to model these uncertainties using variational interpretable concept embeddings (VICE^55^), a recently proposed, approximate Bayesian inference method for learning an interpretable object concept space from human similarity judgements. VICE has shown remarkable performance in predicting the (dis-)agreement in human similarity judgements for multiple similarity judgement datasets, including THINGS^55^.

We train a VICE model on the official THINGS train triplet dataset using the (default) hyperparameters recommended by the authors. To capture the uncertainties in human triplet responses, VICE learns a mean, $\mu \in {{\mathbb{R}}}^{m\times d}$, and a variance, $\sigma \in {{\mathbb{R}}}^{m\times d}$, for each object image *m* and each object dimension *d*, respectively. Therefore, the set of VICE parameters is defined as $\theta =\{\mu ,\sigma \}$. VICE uses the reparameterization trick^56,57^ to generate an embedding matrix ${\bf{Y}}\in {{\mathbb{R}}}^{m\times d}$, ${{\bf{Y}}}_{\theta ,{\varepsilon }}=\mu +\sigma \odot {\varepsilon }$, where $\varepsilon \in {{\mathbb{R}}}^{m\times d}$ is entrywise *N*(0, 1), and ⊙ denotes the Hadamard (element-wise) product.

After convergence, we can use a VICE model to obtain a posterior probability distribution for each triplet in the data. We approximate the probability distribution using a Monte Carlo estimate^58–60^ from *R* samples ${{\bf{Y}}}^{({\bf{r}})}={{\bf{Y}}}_{\hat{\theta },\varepsilon (r)}$ for *r* = 1, …, *R*, yielding

$$\hat{p}(\{\,{y}_{s},\,{z}_{s}\}|\{{i}_{s},\,{j}_{s},\,{k}_{s}\}):= -\frac{1}{R}\mathop{\sum }\limits_{r=1}^{R}\mathop{\underbrace{p(\{\,{{\rm{y}}}_{s},\,{z}_{s}\}|\{{i}_{{\rm{s}}},{j}_{s},{k}_{s}\},{{\bf{Y}}}^{(r)})}}\limits_{{\rm{M}}{\rm{o}}{\rm{n}}{\rm{t}}{\rm{e}}-{\rm{C}}{\rm{a}}{\rm{r}}{\rm{l}}{\rm{o}}\,{\rm{e}}{\rm{s}}{\rm{t}}{\rm{i}}{\rm{m}}{\rm{a}}{\rm{t}}{\rm{e}}}$$

where we set *R* = 50 because we found it to yield the best predictive performance on the official THINGS validation set. This gives a representative probability estimate for each of the three pairs in a triplet to be selected as the most similar pair.

##### Soft-alignment loss

Using the posterior probability estimates obtained from VICE, we transform the original THINGS triplet dataset of discrete triplet choices into a triplet dataset of probability distributions that reflect the human uncertainties of the triplet alternatives. Let ${D}^{\dagger }\,:= \,{({p}_{s}^{\ast }(\{{i}_{s},{j}_{s}\,,\,{k}_{s}\}))}_{s=1}^{n}$ be the transformed triplet dataset, where

$${p}_{s}^{\ast }(\{{i}_{s},{j}_{s}\,,\,{k}_{s}\}):= \hat{p}(\{\,{y}_{s},{z}_{s}\}|\{{i}_{s},{j}_{s}\,,\,{k}_{s}\})\,{\rm{\forall }}\,\{\,y,z\}\subset \{i,j\,,\,k\}.$$

Now, for *n* triplet responses we can define the negative log-likelihood for the soft alignment loss as

3 $$\begin{array}{l}{L}_{{\rm{s}}{\rm{o}}{\rm{f}}{\rm{t}}\mbox{-}{\rm{a}}{\rm{l}}{\rm{i}}{\rm{g}}{\rm{n}}}({{\bf{S}}}^{^{\prime} })\,:= \,\frac{1}{n}\mathop{\sum }\limits_{s=1}^{n}{p}_{s}^{\ast }(\{{i}_{s},{j}_{s}\,,\,{k}_{s}\})\mathrm{log}{p}_{s}^{\ast }(\{{i}_{s},{j}_{s}\,,\,{k}_{s}\})\\ \,\,\,\,\,\,-{p}_{s}^{\ast }(\{{i}_{s},{j}_{s}\,,\,{k}_{s}\})\mathrm{log}{q}_{s}^{\ast }(\{{i}_{s},{j}_{s}\,,\,{k}_{s}\})\end{array}$$

where $${q}_{s}(\{{i}_{s},{j}_{s}\,,\,{k}_{s}\},{\bf{S}})\,:= \,q(\{\,{y}_{s},{z}_{s}\}|\{{i}_{s},{j}_{s}\,,\,{k}_{s}\},{\bf{S}})\,{\rm{\forall }}\,\{\,y,z\}\subset \{i,j,k\}.$$

##### Uncertainty distillation

We mainly follow the optimization process introduced in ref. ^61^. However, we modify their approach by injecting uncertainty measures about human odd-one-out responses into the representation space of the teacher, using a recent approximate Bayesian inference method for learning object concepts from human behaviour^55^. Thus, we replace the negative log-likelihood of the discrete human odd-one-out choices—which we refer to as hard alignment—with the negative log-likelihood of the probabilities for the pairwise triplet similarities obtained from the Bayesian inference model—referred to as soft alignment. The final objective for learning the uncertainty distillation transformation is thus defined as

4 $$\mbox{arg}\,\mathop{\mbox{min}}\limits_{W,b}\,{L}_{\mbox{soft-align}}({\bf{X}},{\bf{W}},{\bf{b}})+\lambda \Vert {\bf{W}}-(\mathop{\sum }\limits_{j=1}^{p}{W}_{jj}/p){\bf{I}}{\Vert }_{F}^{2},$$

where $I\in {{\mathbb{R}}}^{p\times p}$ is the identity matrix and ||.||_F_^2^ denotes the squared Frobenius norm. The right-hand side of the above objective is an ℓ_2_-regularization whose aim is to preserve the nearest-neighbour information (or equivalently, the local similarity structure) of the pretrained representations while learning an affine transformation into the THINGS human object similarity space. The above equation is minimized using standard stochastic gradient descent.

Although this expression is similar to the global transform defined in ref. ^61^, we find it to yield equally strong downstream task performance as the gLocal transform proposed in ref. ^61^ while predicting human uncertainties better than the global transform. It appears as though there is barely any trade-off between representational alignment and downstream task performance for using the uncertainty distillation, whereas ref. ^61^ found that the global transform yields slightly better human alignment but worse downstream task performance compared wit the gLocal transform. We use the uncertainty distillation transformation for generating human-like similarity judgements by transforming a model’s representation space with uncertainty distillation.

#### Data generation

In the following section, we describe the AligNet data-generation process. We start by introducing the data that we use for constructing the triplets. We continue with a detailed description of the different sampling strategies that we consider in our analyses. Finally, we explain how we collect model responses using transformed representations and define the objective function for fine-tuning models on AligNet.

##### Image data

For creating AligNet, we use the publicly available ImageNet database^38^. ImageNet is a natural image dataset with approximately 10^6^ training data points and 1,000 image categories^28^. The categories are almost equally distributed in the data with small variations in the number of images between the different classes. Hence, ImageNet can be considered a highly balanced dataset. ImageNet has been the dominant image dataset for training large computer vision models until the advent of image/text multimodal training a few years ago. Although, so far, larger image datasets exist, ImageNet is still is one of the largest open-source and most widely used image datasets in the field of computer vision.

##### Triplet sampling

For generating triplets of images, we use three different sampling strategies: random, class-border and cluster-boundary sampling. Let *m*′ be the number of images in the data where *m*′ = 1, 281, 167 and *C* be the number of classes with *C *= 1,000. Let ${D}_{{\rm{i}}{\rm{m}}{\rm{a}}{\rm{g}}{\rm{e}}}\,:= \,{({x}_{i},\,{y}_{i})}_{i=1}^{{m}^{^{\prime} }}$ be the ImageNet dataset of *m*′ image–label pairs.

##### Random

Uniform random sampling is the vanilla sampling approach used to create the THINGS datasets (see above). In random sampling, three images are chosen uniformly at random without replacement from all of the *m* images in the data to create a triplet. As there are *C* = 1,000 classes and each class has approximately 1,000 images, most of the triplets generated with this approach contain 3 images from 3 different classes. The number of triplets different from triplets with images from three distinct classes is negligible. It is noted that this is the same sampling approach that was used to generate the THINGS triplets^54^. A triplet generated via random sampling can be defined as the following triplet set ${\bf{S}}:= \{{x}_{i},{x}_{j},{x}_{k}\}$ with the constraint $({x}_{i}\ne {x}_{j}\ne {x}_{k})$.

##### Class boundary

Another way to sample image triplets is to exploit the label information associated with each data point. Instead of three random images from three distinct classes, we determine class-boundary triplets to contain two images from the same class and one image from a different class. This is similar to the approach introduced in ref. ^62^ where each odd-*k*-out set of images contains a majority class and *k* odd class singletons. This sampling approach allows models to learn class boundaries similar to the standard supervised learning setting. A triplet generated via class-boundary sampling can be defined as the following triplet set ${\bf{S}}:= \{{x}_{i},\,{x}_{j},\,{x}_{k}\}$ with the constraint $(\,{y}_{i}={y}_{j}\ne {y}_{k})\vee (\,{y}_{i}\ne {y}_{j}={y}_{k})\vee (\,{y}_{i}={y}_{k}\ne {y}_{j})$ where the labels are used for data partitioning.

##### Cluster boundary

As we want to introduce a general approach that does not rely on label information, we use a third sampling strategy that is, in principle, similar to the class-boundary approach but does not require labels. Let ${\bf{Z}}\in {{\mathbb{R}}}^{{m}^{^{\prime} }\times p}$ be the stacked representations of a neural network model for every image in *D*_image_. The representations can essentially be computed for any layer of a model. Here we use the image encoder for image/text models and the CLS token representation of the penultimate layer for any other model (as we only use ViT-based models). We then apply *k*-means clustering to the encoded image representations **Z** and ${{\bf{Z}}}^{^{\prime} }\,:= \,{({\bf{W}}{{\bf{Z}}}^{{\rm{\top }}}+({{\bf{b}}}_{1},\ldots ,{{\bf{b}}}_{{m}^{^{\prime} }}))}^{{\rm{\top }}}$ respectively (where the transformation variables **W** and **b** are computed via uncertainty distillation optimization using equation (4)) into *c* representation clusters where *c* can be regarded as similar to *C*, the number of labels in the original dataset. We use the Elbow criterion to select *c*. For all of our main experiments, we set *c* = 500. Hence, the ImageNet dataset is transformed into a ImageNet dataset of image and cluster pairs. After the clustering, we apply the same sampling method as for class-boundary triplets: for each triplet, we choose uniformly at random two images without replacement from one cluster and one image from a different cluster. Thus, a triplet generated via cluster-boundary sampling can be defined as the following set ${\bf{S}}:= \{{x}_{i},{x}_{j},{x}_{k}\}$ with the constraint $({y}_{i}={y}_{j}\ne {y}_{k})\vee ({y}_{i}\ne {y}_{j}={y}_{k})\vee ({y}_{i}={y}_{k}\ne {y}_{j})$ where instead of the original labels we use the cluster labels for partitioning the data.

##### Triplet-response generation

We use the responses of a surrogate teacher model (see below) to simulate a dataset of human-aligned triplet odd-one-out responses. More formally, let ${D}_{\mbox{triplets}}\,:= \,{(\{{x}_{i},\,{x}_{j},\,{x}_{k}\})}_{s=1}^{{n}^{^{\prime} }}$ be the dataset of sampled ImageNet triplets for which we want to collect responses using transformed model representations. It is noted that we can sample an arbitrary number of triplets—upper-bounded by the binomial coefficient *m*′/*k* with *k* = 3—and can thus set *n*′ to essentially any natural number. For the experiments that we report in the main text, we set *n*′ = 10^7^ because we found a larger *n*′ to not yield any downstream task improvements. For now, we regard our surrogate model as a blackbox model with transformed ImageNet representations ${{\bf{Z}}}^{^{\prime} }\,:= \,{({\bf{W}}{{\boldsymbol{Z}}}^{{\rm{\top }}}+({{\bf{b}}}_{1},\ldots ,{{\bf{b}}}_{{m}^{^{\prime} }}))}^{{\rm{\top }}}\in {{\mathbb{R}}}^{{m}^{^{\prime} }\times p}$ where the affine transformation was found via uncertainty distillation optimization (equation (4)). Given transformed representations *z*_1_′, *z*_2_′ and *z*_3_′ of the three images in a triplet, we can construct a similarity matrix ${{\bf{S}}}^{^{\prime} }\in {{\mathbb{R}}}^{3\times 3}$ where ${{S}^{^{\prime} }}_{i,j}:={{\rm{z}}}_{i}^{{\rm{\top }}}{{\rm{z}}}_{j}$ is the dot product between a pair of of representations. Similarly to how we do this for learning the uncertainty distillation transformation (see above), we identify the closest pair of images in a triplet as $\arg \mathop{max}\limits_{i,j > i}{S}_{i,j}^{^{\prime} }$ with the remaining image being the odd one out. Let ${D}_{{\rm{a}}{\rm{l}}{\rm{i}}{\rm{g}}{\rm{n}}}\,:= \,{({\{{x}_{a},{x}_{b}\}}_{s}|\{{x}_{i},{x}_{j},{x}_{k}\})}_{s=1}^{{n}^{^{\prime} }}$ then constitute the final AligNet dataset of ImageNet triplets and corresponding model responses, where $\{{x}_{a},{x}_{b}\}\subset \{{x}_{i},{x}_{j},{x}_{k}\}$ and $\{{x}_{a},{x}_{b}\}$ is the image pair that was chosen by the transformed model representations to have the highest pairwise similarity. The model choices are the closest approximation to the human choices due to the uncertainty distillation transformation.

It is noted that the dataset includes not only the discrete model choices but also the exact relationships among all pairwise similarities in a triplet obtained from the probability space of the teacher model. Thus, we have access to soft distributions over the labels for use in distillation.

#### Objective function

Let *f*_*θ*_ be a neural network function parameterized by *θ*, the set of its weights and biases. For every input image *x* that the function *f*_*θ*_(*x*) processes it yields a representation *f*_*θ*_(*x*) = z. Here, *z* refers to the image encoder representation of image/text models or the CLS token representation before the final linear layer for other model types. From the representations of the three images in a triplet, we can again construct a similarity matrix ${{\boldsymbol{S}}}^{\dagger }\in {{\mathbb{R}}}^{3\times 3}$ where ${S}^{\dagger }:= {{\rm{z}}}_{i}^{{\rm{\top }}}{{\rm{z}}}_{j}$ is the dot product between a pair of image representations. The AligNet loss function is defined as the following KL divergence between teacher and student triplet probabilities,

5 $$\begin{array}{c}{L}_{\mbox{alignet}}({{\bf{S}}}^{^{\prime} },{{\bf{S}}}^{\dagger })\,:= \,\\ \,-\frac{1}{B}\mathop{\sum }\limits_{s=1}^{B}{\sigma }{([{S}_{i,j}^{^{\prime} },{S}_{i,k}^{^{\prime} },{S}_{j,k}^{^{\prime} }],{\tau }^{^{\prime} })}_{s}\mathrm{log}\,{\sigma }{([{S}_{i,j}^{^{\prime} },{S}_{i,k}^{^{\prime} },{S}_{j,k}^{^{\prime} }],{\tau }^{^{\prime} })}_{s}\\ \,-{\sigma }{([{S}_{i,j}^{^{\prime} },{S}_{i,k}^{^{\prime} },{S}_{j,k}^{^{\prime} }],{\tau }^{^{\prime} })}_{s}\mathrm{log}\,{\sigma }{([{S}_{i,j}^{\dagger },{S}_{i,k}^{\dagger },{S}_{j,k}^{\dagger }],{\tau }^{\dagger })}_{s},\end{array}$$

where *τ*′ = 1 and *τ*^†^ > 1 and *B* is the batch size. We find *τ*^†^ via grid search and set it to *τ*^†^ = 100 for all of our experiments. Recall that *σ* is a softmax function that models the probabilities over the three image similarity pairs (equation (1)). The final AligNet objective is defined as the following minimization problem

6 $$\mbox{arg}\,\mathop{\mbox{min}}\limits_{\theta }\,{L}_{\mbox{alignet}}({f}_{\theta })+\lambda {\Vert {\theta }^{\ast }-{\theta }^{\dagger }\Vert }_{2}^{2}$$

where θ^*^ are the parameters of the pretrained base student model and *θ*^†^ are the parameters of the fine-tuned student model. This ℓ_2_-regularization, which we refer to as weight decay to initialization, encourages the fine-tuned set of parameters to stay close to its base during training. It is similar to the regularization used for learning the uncertainty distillation transformation (equation (4)) but adapted to the set of all model parameters rather than a linear transform.

#### Surrogate teacher model

Reference ^25^ showed that image and text models and models trained on large, diverse datasets are better aligned with human similarity judgements than vision models trained with a self-supervised learning objective or supervised models trained on ImageNet. Thus, we use the best-performing image and text model according to various computer vision benchmarks at the time of writing this paper as our teacher model. This model is called SigLIP^63^. SigLIP, similar to contrastive language–image pretraining (CLIP)^64^ and ALIGN^65^, is trained via contrastive language–image pretraining using millions of image and text pairs. The difference between CLIP and SigLIP is that the latter uses a paired sigmoid loss instead of the standard softmax function usually used for pretraining image and text models via cross-entropy. Image and text pretraining allows the model to learn an aligned representation space for images and text; thus, adding more semantic information about the objects in an image to the model representations.

We use the SigLIP-So400m variant of SigLIP as our teacher model. This variant uses an optimized ViT backbone whose performance is similar to one of the largest ViTs, ViT-g/14^66^ while having fewer parameters and thus being smaller. The number of parameters of SoViT-400m/14 lies somewhere between that of ViT-L/16 and ViT-g/14. The output dimensionality of the image and text encoder representations of SoViT-400m/14 is *p *= 1,152 each. We align the image encoder representations with human odd-one-out choices using the uncertainty distillation optimization outlined in equation (6). This allows us to increase the triplet odd-one-out accuracy of SigLIP-So400m from 44.24% to 61.7% (rightmost column in Supplementary Table 1), which is close to the human noise ceiling of 66.67% for THINGS (compare ref. ^54^) and thus among the best human-aligned models without AligNet fine-tuning (compare ref. ^25^). It is noted that this is a relative increase in performance of 39.47%. Throughout this paper, we use the human-aligned version of SigLIP-So400m as the surrogate teacher model for generating human-aligned similarity judgements and distiling human-like similarity structure into student vision foundation models (VFMs). We select a diverse and representative set of student VFMs.

#### Student models

As previous research has demonstrated that a model’s architecture has no significant impact on the degree of alignment with human similarity judgements^25,61^, we use the same architecture for all student models that we fine-tune on AligNet. Specifically, we use the ViT^8^ for the backbone of each student model. We use the ViT rather than a convolutional-neural-network-based model because ViTs have recently emerged as the dominant neural network architecture for computer vision application and VFMs. Every large VFM used in practice is based on the ViT^30,63,67,68^. Unless otherwise mentioned, we use the base model size, that is, ViT-B. ViT-B has 12 attention layers and an internal (hidden) representation size of *p* = 768. It has been shown that both the training data and the objective function have a substantial impact on the degree of alignment with human behaviour. Thus, we use student models that were trained on different pretraining task with different training data and objective functions.

Supervised pretraining is still the prevailing mode of training computer vision models. Therefore, we trained ViT-B on the popular ImageNet dataset consisting of 1.4 million natural images^38^. To examine how model performance changes as a function of the model size, we train ViT instances of three different sizes on ImageNet: ViT-S/16, ViT-B/16 and ViT-L/16. The image patch size is the same for each of those models. To evaluate the effect of AligNet on self-supervised pretraining, we use pretrained DINOv1^69^ and DINOv2^29^ models of which DINOv1 was pretrained on ImageNet and DINOv2 was pretrained on a different, larger image dataset as denoted below. In addition, we investigate multimodal training of vision models that add textual information in the form of both image captioning via the CapPa model^30^ and CLIP via SigLIP^63^. The latter model is considered state of the art on many downstream computer vision applications and is used as the image embedding model in modern large visual-language models^67,68^. The full list of student models that we consider in our analyses is as follows:

- ViT-{S,B,L}
  - Training data: ImageNet^38^
  - Objective: supervised learning

- CLIP (ViT-B) (see Appendix A.2.4)
  - Training data: WebImageText^64^
  - Objective: CLIP^64^

- SigLIP (ViT-{B,SO400M})
  - Training data: WebLI^63^
  - Objective: CLIP^64^

- SigLIP2 (ViT-B) (Supplementary Information section 2.4)
  - Training data: WebLI^63^
  - Objective: combination of various objectives (see ref. ^70^ for details)

- DINOv1 (ViT-B)
  - Training data: ImageNet^38^
  - Objective: self-supervised image pretraining^69^

- DINOv2 (ViT-B)
  - Training data: DINOv2 data (see ref. ^29^ for details)
  - Objective: self-supervised teacher-student distillation^29^

- CapPa (ViT-B)
  - Training data: JFT-3B (Google proprietary dataset)
  - Objective: multimodal image captioning^30^

- Randomly initialized ViT-B (Supplementary Information section 2.5)
  - Training data: AligNet
  - Objective: AligNet objective (equation (5))

### Representational similarity analysis

Representational similarity analysis is a well-established method for comparing neural network representations—extracted from an arbitrary layer of the model—to representations obtained from human behaviour^44^. In representational similarity analysis, one first obtains representational similarity matrices (RSMs) for the human behavioural judgements and for the neural network representations (more specific details can be found in Supplementary Information). These RSMs measure the similarity between pairs of examples according to each source. As in previous work^23,25,54,61,71^, we flatten the upper triangular of human and model RSMs respectively and quantify their similarities using use the Spearman rank correlation coefficient. In contrast to Pearson correlation, the Spearman rank correlation is scale invariant and thus better suited to measure similarities of judgements obtained from different sources.

#### Multi-arrangement task

Human similarity judgements for refs. ^23,71^ were obtained by using a multi-arrangement task. In a multi-arrangement task, participants are presented with a computer screen showing images of several different objects. The participants are asked to arrange the images into semantically meaningful clusters, given the instruction that images of objects that lie close together are considered more similar. From this arrangement, one can infer pairwise (dis-)similarities of the objects and average those across all participants to obtain a representative (dis-)similarity matrix.

#### Likert scale

In refs. ^24,72^, pairwise similarity judgements were obtained by asking human participants to rate the similarity of pairs of objects on an ordinal scale that ranges from 0 (‘not similar at all’) to 10 (‘very similar’). The pairwise similarity ratings can be averaged across the different participants, which in turn yields a matrix of similarities between pairs of objects.

#### Neural network representations

RSMs for neural network representations are obtained by first embedding the same set of images that were presented to the human participants in the *p*-dimensional latent space of a model. The latent space could be any layer of a neural network. For the base models, we use the representations of the image encoder for SigLIO and the CLS token of the penultimate layer for CapPa, DINOv2 and ViT-B. We do this because previous work has shown that the penultimate layer space and the image encoder space of image and text models, respectively, yield the highest similarity to human behaviour^24,25,73^. After embedding the images into the neural net’s latent space, we get a representation matrix ${\bf{X}}\in {{\mathbb{R}}}^{n\times p}$ for the *n* images in the data. Instead of simply computing the dot-product similarity matrix ${\bf{S}}:= {\bf{X}}{{\bf{X}}}^{{\rm{\top }}}$, in representational similarity analysis one typically uses either a cosine similarity or a Pearson correlation kernel to compute the affinity matrix

$$\cos ({{\bf{x}}}_{i},{{\bf{x}}}_{j})\,:= \,\frac{{{{\bf{x}}}_{i}}^{{\rm{\top }}}{{\bf{x}}}_{j}}{{\Vert {{\bf{x}}}_{i}\Vert }_{2}{\Vert {{\bf{x}}}_{j}\Vert }_{2}};\,\,\,\,\,\phi ({{\bf{x}}}_{i},{{\bf{x}}}_{j})\,:= \,\frac{{({{\bf{x}}}_{i}-{\bar{{\bf{x}}}}_{i})}^{{\rm{\top }}}({{\bf{x}}}_{j}-{\bar{{\bf{x}}}}_{j})}{{\Vert {{\bf{x}}}_{i}-{\bar{{\bf{x}}}}_{i}\Vert }_{2}{\Vert {{\bf{x}}}_{j}-{\bar{{\bf{x}}}}_{j}\Vert }_{2}},$$

where the cosine similarity kernel function cos(**x**_*i*_, **x**_*j*_) or the Pearson correlation kernel function *ϕ*(**x**_*i*_, **x**_*j*_) is applied to every (**x**_*i*_, **x**_*j*_) vector pair of the matrix **X** for obtaining the final RSM ${{\bf{S}}}^{^{\prime} }\in {{\mathbb{R}}}^{n\times n}$. Here we use the Pearson correlation kernel function *ϕ*(**x**_*i*_, **x**_*j*_) to obtain a neural net’s RSM. Pearson correlation is the centred version of cosine similarity and the ranking of the obtained similarities does not differ between the two kernel functions but Pearson correlation first centres the vectors to have zero mean and is therefore a more robust measure. For obtaining RSMs with transformed representations, the transforms are first applied to **X** before computing **S**′.

### Alignment with conceptual hierarchy

When analysing alignment with the conceptual hierarchy, we use the original ImageNet category labels for the images^38^. ImageNet is structured by the WordNet hierarchy, from which we extract basic and superordinate categories aligned with the previous cognitive work. Within and across categories, we measure change in representation distance relative to other changes (by *z*-scoring across all representation distances for the given model checkpoint), because relative distances are more meaningful than absolute ones (for example, scaling all representations by two would change absolute distances, but not relative ones), and absolute scales of all representations tend to increase during training. We quantify changes with mixed-effects linear regressions that account for the non-independence of representational changes across the different clusters (see Supplementary Information section 3.2 for details).

### Levels data

We collected a new multi-level similarity judgement dataset from *N* = 473 human participants, which we named Levels. The dataset contains odd-one-out judgements on three different types of triplet: coarse-grained semantic, which requires deciding on the odd one out in broadly different categories; fine-grained semantic, which involved discerning subtle within category distinctions; and class boundary, which tested for category-boundary detection. Consistent selection of the same odd-one-out image (for example, *i*) in multiple participants indicated that the remaining two images (for example, *j* and *k*) were closer to each other in the participants’ concept space than either was to the odd one out (see Supplementary Information for details about the data collection). Levels allowed us to evaluate model–human alignment for the same set of stimuli on various levels of abstraction, and to assess how well the models capture the inherent uncertainty in human judgements, inferred from response latencies.

#### Participants

We recruited *N* = 508 participants (209 female, 289 male, 3 diverse, *N* = 7 missing demographic information owing to revocation of study consent; mean age 31.75 ± s.d. = 8.04 years) online via Prolific Academic (https://www.prolific.ac). The eligibility criteria were that participants had to be between 18 and 50 years old, fluent in English, have normal or corrected-to-normal vision, no colourblindness, and have a minimum approval rating of 95% on Prolific. Participants provided informed consent before starting the experiment. The experiment lasted approximately 45 minutes. Participants were reimbursed with £7.70 for completing the experiment and received an additional bonus payment of £0.77. Partial payments were made if the experiment was not completed owing to technical issues (*N* = 6) or early termination by the participant (*N* = 1). Participants performing below 90% correct on catch trials (*N* = 19, 3 female, 16 male), or failing to respond in the allotted time window (15 s) in more than 10 trials (*N* = 9, 4 female, 4 male, 1 diverse) were excluded. Thus, *N* = 473 participants remained in the dataset (202 female, 269 male, 2 diverse; mean age 31.82 ± s.d. = 8.03 years). Of these participants, *N* = 448 were each tested with a different selection of triplets, while ensuring that each triplet was presented *N* = 5 times across the entire sample of participants (see information on stimuli sampling below). Owing to a server glitch during trial assignment, the remaining *N* = 25 participants shared their exact triplet selection with one other participant in the sample. These *N* = 25 participants were excluded from the response times and uncertainty estimation (see ‘Alignment at multiple levels of abstraction’ section) to restrict analysis to participants with different sets of triplets. The experiment was approved by the internal review board of the Max Planck Institute for Human Development.

#### Stimuli

The experimental stimuli were images taken from the ImageNet dataset^38^. Another nine images were used for instructions only and depicted natural objects selected from the Bank of Standardized Stimuli (BOSS)^74^, available at https://drive.google.com/drive/folders/1FpnEFkbqe_huRwfsCf7gs5R1zuc1ZOkn. We grouped the visual stimuli presented in the triplets according to different levels of abstraction: coarse-grained semantic, which comprised three images from three different categories; fine-grained semantic, showing three images from the same category; and class boundary, where two images were from the same and one from a different category.

Instead of randomly sampling triplets—which would reproduce dataset biases—we stratified sampling by superclasses. ImageNet classes follow the WordNet hierarchy^28,38^, which includes higher-level classes. For instance, all dog breeds can be summarized as the dog superclass. To avoid presenting dogs, birds and other fine-grained classes that are overrepresented in ImageNet more frequently to the participants than other categories, we grouped the ImageNet classes into 717 coarse-grained WordNet superclasses. We uniformly at random sampled images from those 717 superclasses to construct the different kinds of triplets. It is noted that for all superclasses with more than one class, we uniformly at random chose one subclass and uniformly at random sampled one image, two images (without replacement) or three images (without replacement) from that subclass, depending on the triplet type. For most superclasses that comprised a single subclass only, that is, a one-to-one-mapping, we could skip the subclass sampling part. Triplet sampling resulted in *N* = 450 predefined experiment trial sets, of which *N *= 448 were used for testing. Across these, each triplet was presented within *N* = 5 different experiment files. This sampling process ensured a balanced distribution of triplets across the sample, and the repetition of each triplet in five different participants allowed for the calculation of an uncertainty distribution for each triplet.

#### The triplet odd-one-out task

On each trial, participants were presented with a triplet of images (*i*, *j*, *k*). Participants were asked to select the image that was the most different from the other two, that is, the odd one out. During the instructions, participants saw different triplets with increasing ambiguity regarding which image would likely be picked as the odd one out. Participants were given explanations for potential odd-one-out choices, clarifying that decisions could be based on different criteria, such as semantic or perceptual features of the shown images.

#### Procedure

The experiment was run online using jsPsych v7.3.3 (www.jspsych.org/7.3/) and custom plugins. Participants were asked to provide demographic information, including their age and gender. Thereafter, they viewed written instructions about the task and performed six practice trials (two trials per triplet level of abstraction). Participants were free to repeat the instructions until they felt confident to perform the experiment. The experiment proper comprised *N* = 330 experiment trials. Each trial started with a fixation cross (1 s), followed by the presentation of a triplet (maximum 15 s). Participants were asked to select the ‘odd one out’ using the right-, left- or downwards-facing arrow keys on their keyboard. Responses could be entered between 1 s and 15 s after triplet onset, after which the next trial started. Trials in which participants failed to submit a response were rare (*M* = 0.27% of trials; minimum 0.00%, maximum 6.06%). The serial order of triplet types (for example, fine-grained or coarse-grained semantic) and ImageNet classes (for example, dogs or birds) was counterbalanced across the experiment. We additionally counterbalanced the serial position of trial types across participants using a Latin-square design^75^. Participants could take short breaks (self-paced for up to 2 min) after *N* = 50, 150 and 200 experiment trials. Experimental trials were interleaved with *N* = 16 catch trials (class-border triplets), which were predefined based on low model uncertainty and 100% agreement among participants on these specific triplets during piloting. Catch trial performance was used as an indicator of adequate task engagement (see participant inclusion criteria above).

#### Preprocessing of human response times and uncertainty estimation

Descriptive statistics on response times and uncertainty estimation (see ‘Alignment at multiple levels of abstraction’ section) were calculated based on participants with unique experimental trial sets (*N* = 448). The response-time data were log transformed (log(RT)), in accordance with current best practices for response-time analysis. Trials with response times longer than 10 s were excluded from analysis (on average *M* = 2.64% of trials per participant). As responses could be given no earlier than 1 s after triplet onset (see ‘Procedure’ above), no lower bound was set for response-time exclusion. To estimate uncertainty (in terms of the level of (dis-)agreement among observers) for each triplet, we used the discrete (Shannon) entropy of the response distribution across participants.

#### Human-to-human alignment

We computed the human noise ceiling for each abstraction setting in Levels using a leave-one-out cross-validation approach. In leave one out, the agreement level for a triplet is computed as the average match rate between a held-out participant’s response and the majority response of the remaining population. Thus, for a triplet that was used for five participants, on each leave-one-out iteration, one participant response is held out and the remaining four comprise the population. The human-to-human reliability score is then calculated as the average agreement level across all triplets in the dataset.

### Reporting summary

Further information on research design is available in the Nature Portfolio Reporting Summary linked to this article.

## Online content

Any methods, additional references, Nature Portfolio reporting summaries, source data, extended data, supplementary information, acknowledgements, peer review information; details of author contributions and competing interests; and statements of data and code availability are available at 10.1038/s41586-025-09631-6.

## Supplementary information

Supplementary Information Supplementary Notes, including the detailed results of statistical models, and other analysis details (9 tables and 15 figures).

Reporting Summary

## Figures and Tables

**Extended Data Fig. 1:**
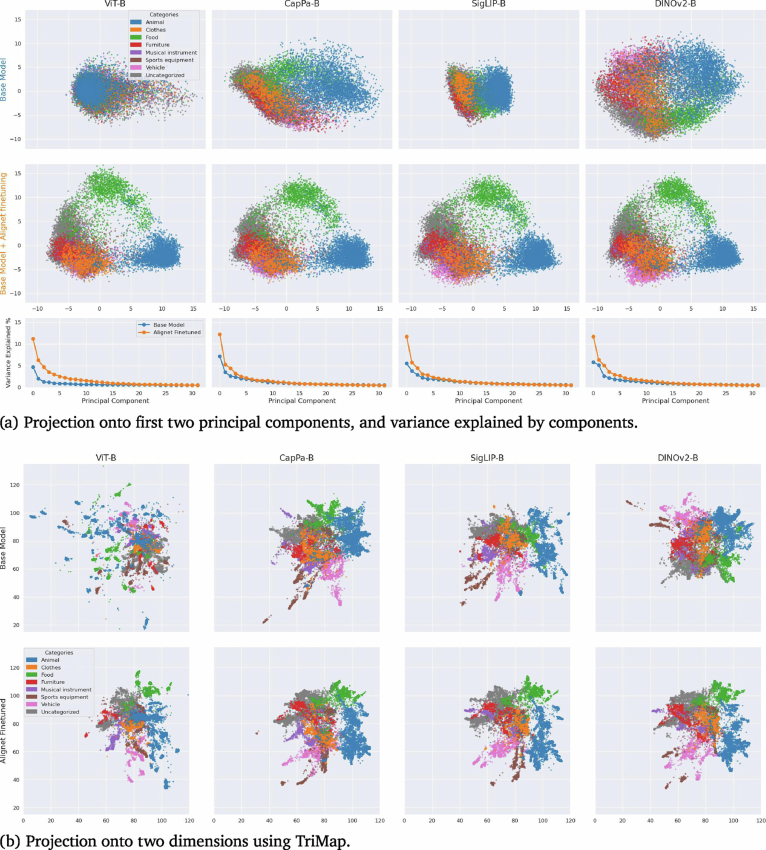
Visualizing the representational reorganization in two dimensions using PCA and TriMap. Each panel shows a low-dimensional visualization (a: PCA; b: TriMap) the representations of 85k ImageNet images from four different models (columns) onto their first two principal components both before (first row) and after fine-tuning on AligNet (second row). Colors correspond to high-level categories of the images. As shown in both visualizations, AligNet training results in more systematic differentiation of the high-level categories, and more consistent organization across the models. (The third row of the top panel visualizes the proportion of explained variance for the first 32 principal components).

**Extended Data Fig. 4:**
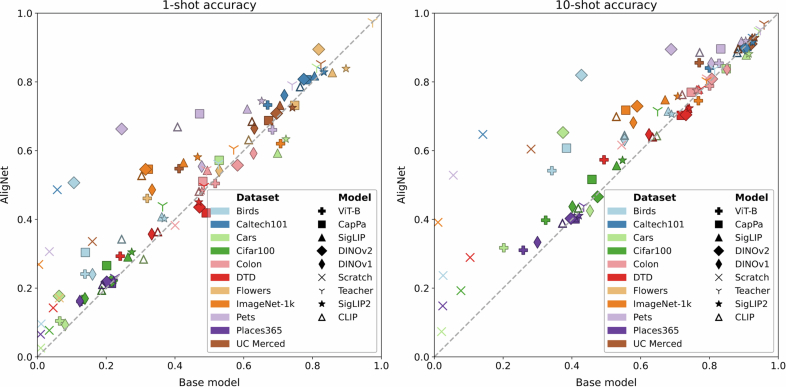
1-shot and 10-shot linear probing performance on 11 common datasets. This plots compares the few-shot performances of the pre-trained (base) models vs. their performance after being aligned-finetuned. AligNet finetuning is clearly beneficial on most datasets in both 1-shot and 10-shot evaluations. Even models that have previously been trained on ImageNet data (e.g. DINOv1 ViT-B/16 and supervised ViT-B/16) show improvements on 1-shot ImageNet performance, which indicates that the benefits do not merely come from being exposed to ImageNet data, but also from the label information we distilled into AligNet. This plot also includes results for the widely known CLIP^18^ model. It generally performs worse compared to the SigLIP model, which is why we picked the latter as the representative text/image model. For the more recent SigLIP2^27^ we instead find that it has even stronger baseline performance than SigLIP, but it still does benefit in most cases from AligNet finetuning.

**Extended Data Fig. 5:**
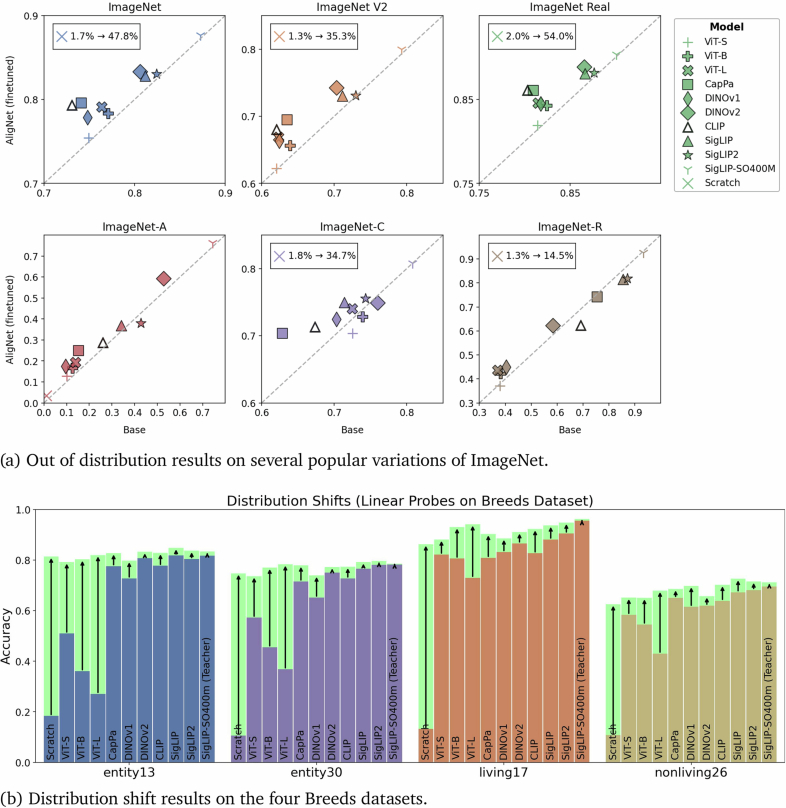
AligNet improves out-of-distribution generalization, and robustness wrt. distributional shifts. (a) Accuracy of a linear readout head trained on top of the (frozen) base models is plotted on the *x*-axis, against the corresponding performance a linear readout head trained on top of the (frozen) AligNet-finetuned models on the *y*-axis. Every symbol above the diagonal indicates improved performance (through AligNet), while symbols below the diagonal indicate a performance degradation. Note that for better readability the range of the plot is zoomed in on the pre-trained models. The model trained from scratch (×) is thus often out of bounds due to its substantially lower performance, and its accuracy before and after finetuning is instead written out in a small inset. (b) Additional results for all student models on the four Breeds datasets: “entity13”, “entity30”, “living17”, and “nonliving26”. Student models are sorted according to their objective function (supervised, captioning, self-supervised, image/text). The randomly initialized ViT-B has the poorest base performance but benefits the most (leftmost column in each panel). Conversely, SigLIP-So400m—the teacher model—has the strongest base performance and benefits the least but consistently (rightmost column in each panel). Vertical arrows indicate improvements in performance compared to the base performance.

## Data Availability

The synthetically created AligNet data are publicly available at https://console.cloud.google.com/storage/browser/alignet. The Levels data are available on GIN at 10.12751/g-node.hg4tdz.

## Code availability

The training code is available at https://github.com/google-deepmind/alignet. The aligned model checkpoints are publicly available at https://console.cloud.google.com/storage/browser/alignet. Both the experiment and analysis code for Levels and for the human validation of the representational differences between AligNet and UnaligNet are hosted on GitHub and archived on Zenodo at https://zenodo.org/records/15554034 (ref. ^76^) and https://zenodo.org/records/15554174 (ref. ^77^).

## References

[1] Khaligh-Razavi, S.-M. & Kriegeskorte, N. Deep supervised, but not unsupervised, models may explain it cortical representation. *PLoS Comput. Biol.* **10**, e1003915 (2014). 25375136 10.1371/journal.pcbi.1003915 PMC4222664

[2] Peterson, J. C., Battleday, R. M., Griffiths, T. L. & Russakovsky, O. Human uncertainty makes classification more robust. In *Proc. IEEE/CVF International Conference on Computer Vision (ICCV)* 9616–9625 (IEEE, 2019).

[3] Lake, B. M., Ullman, T. D., Tenenbaum, J. B. & Gershman, S. J. Building machines that learn and think like people. *Behav. Brain Sci.* **40**, e253 (2017). 27881212 10.1017/S0140525X16001837

[4] Geirhos, R. et al. Generalisation in humans and deep neural networks. In *Proc. Advances in Neural Information Processing Systems* Vol. 31 (eds Bengio, S. et al.) 7538–7550 (Curran Associates, 2018).

[5] Hebart, M. N., Zheng, C. Y., Pereira, F. & Baker, C. I. Revealing the multidimensional mental representations of natural objects underlying human similarity judgements. *Nat. Hum. Behav.* **4**, 1173–1185 (2020). 33046861 10.1038/s41562-020-00951-3 PMC7666026

[6] Brown, T. et al. Language models are few-shot learners. In *Proc. Advances in Neural Information Processing Systems* Vol. 33 (eds Larochelle, H. et al.) 1877–1901 (Curran Associates, 2020).

[7] Radford, A. et al. Learning transferable visual models from natural language supervision. In *Proc. 38th Interna- tional Conference on Machine Learning:**Proc. Machine Learning Research* Vol. 139 (eds Meila, M. & Zhang, T.) 8748–8763 (PMLR, 2021).

[8] Dosovitskiy, A. et al. An image is worth 16×16 words: transformers for image recognition at scale. In *Proc. The Ninth**International Conference on Learning Representations* (ICLR, 2021).

[9] Zhai, X., Mustafa, B., Kolesnikov, A. & Beyer, L. Sigmoid Loss for language image pre-training. In *Proc. IEEE/CVF International Conference on Computer Vision* 1975–11986 (IEEE, 2023).

[10] Lapuschkin, S. et al. Unmasking clever Hans predictors and assessing what machines really learn. *Nat. Commun.* **10**, 1096 (2019). 30858366 10.1038/s41467-019-08987-4 PMC6411769

[11] Geirhos, R. et al. Shortcut learning in deep neural networks. *Nat. Mach. Intell.* **2**, 665–673 (2020).

[12] Bowers, J. S. et al. Deep problems with neural network models of human vision. *Behav. Brain Sci.* **46**, e385 (2022). 10.1017/S0140525X22002813 36453586

[13] Muttenthaler, L. et al. Improving neural network representations using human similarity judgments. In *Proc. Advances in Neural Information Processing Systems* Vol. 36 (eds Oh, A. et al) 50978–51007 (Curran Associates, 2023).

[14] Fodor, J. A. & Pylyshyn, Z. W. Connectionism and cognitive architecture: a critical analysis. *Cognition* **28**, 3–71 (1988). 2450716 10.1016/0010-0277(88)90031-5

[15] Tenenbaum, J. B., Kemp, C., Griffiths, T. L. & Goodman, N. D. How to grow a mind: statistics, structure, and abstraction. *Science* **331**, 1279–1285 (2011). 21393536 10.1126/science.1192788

[16] Lake, B. M., Salakhutdinov, R. & Tenenbaum, J. B. Human-level concept learning through probabilistic program induction. *Science* **350**, 1332–1338 (2015). 26659050 10.1126/science.aab3050

[17] Roads, B. D. & Mozer, M. C. Improving human-machine cooperative classification via cognitive theories of similarity. *Cogn. Sci.* **41**, 1394–1411 (2017). 27445204 10.1111/cogs.12400

[18] Hendrycks, D. et al. The many faces of robustness: a critical analysis of out- of-distribution generalization. In *Proc. IEEE/CVF International Conference on Computer Vision* 8340–8349 (IEEE, 2021).

[19] Pooch, E. H. P., Ballester, P. & Barros, R. C. Can we trust deep learning based diagnosis? The impact of domain shift in chest radiograph classification. In *Proc.**Thoracic Image Analysis: Second International Workshop* (eds Petersen, J. et al.) 74–83 (Springer, 2020).

[20] Minderer, M. et al. Revisiting the calibration of modern neural networks. In *Proc. Advances in Neural Information Processing Systems* Vol. 34 (eds Ranzato, M. et al.) 15682–15694 (Curran Associates, 2021).

[21] Cichy, R. M., Kriegeskorte, N., Jozwik, K. M., van den Bosch, J. J. F. & Charest, I. The spatiotemporal neural dynamics underlying perceived similarity for real-world objects. *NeuroImage* **194**, 12–24 (2019). 30894333 10.1016/j.neuroimage.2019.03.031 PMC6547050

[22] Connolly, A. C., et al. The representation of biological classes in the human brain. *J. Neurosci.* **32**, 2608–2618 (2012). 22357845 10.1523/JNEUROSCI.5547-11.2012 PMC3532035

[23] King, M. L., Groen, I. I. A., Steel, A., Kravitz, D. J. & Baker, C. I. Similarity judgments and cortical visual responses reflect different properties of object and scene categories in naturalistic images. *NeuroImage* **197**, 368–382 (2019). 31054350 10.1016/j.neuroimage.2019.04.079 PMC6591094

[24] Peterson, J. C., Abbott, J. T. & Griffiths, T. L. Evaluating (and improving) the correspondence between deep neural networks and human representations. *Cogn. Sci.* **42**, 2648–2669 (2018). 30178468 10.1111/cogs.12670

[25] Muttenthaler, L., Dippel, J., Linhardt, L., Vandermeulen, R. A. & Kornblith, S. Human alignment of neural network representations. In *Proc.**The Eleventh International Conference on Learning Representations* (ICLR, 2023).

[26] Hebart, M. N. et al. THINGS: a database of 1,854 object concepts and more than 26,000 naturalistic object images. *PLoS One* **14**, e0223792 (2019). 31613926 10.1371/journal.pone.0223792 PMC6793944

[27] Lee, D.-H. et al. Pseudo-label: the simple and efficient semi-supervised learning method for deep neural networks. In *Proc.**Workshop on Challenges In Representation Learning (WREPL)* 896–902 (ICML, 2013).

[28] Russakovsky, O. et al. Imagenet large scale visual recognition challenge. *Int. J. Comput. Vis.* **115**, 211–252 (2015).

[29] Oquab, M. et al. DINOv2: learning robust visual features without supervision. *Transact. Mach. Learn. Res.* https://openreview.net/forum?id=a68SUt6zFt (2024).

[30] Tschannen, M. et al. Image captioners are scalable vision learners too. In *Proc. Advances in Neural Information Processing Systems* Vol. 36 (eds Oh, A. et al.) 46830–46855 (Curran Associates, 2023).

[31] Ratcliff, R. A theory of memory retrieval. *Psychol. Rev.* **85**, 59 (1978).

[32] Kiani, R., Corthell, L. & Shadlen, M. N. Choice certainty is informed by both evidence and decision time. *Neuron* **84**, 1329–1342 (2014). 25521381 10.1016/j.neuron.2014.12.015 PMC4271191

[33] Gemini Team et al. Gemini 2.5: pushing the frontier with advanced reasoning, multimodality, long context, and next generation agentic capabilities. Preprint at https://arxiv.org/abs/2507.06261 (2025).

[34] Mehrer, J., Spoerer, C. J., Jones, E. C., Kriegeskorte, N. & Kietzmann, T. C. An ecologically motivated image dataset for deep learning yields better models of human vision. *Proc. Natl Acad. Sci. USA* **118**, e2011417118 (2021). 33593900 10.1073/pnas.2011417118 PMC7923360

[35] Rosch, E., Mervis, C. B., Gray, W. D., Johnson, D. M. & Boyes-Braem, P. Basic objects in natural categories. *Cogn. Psychol.* **8**, 382–439 (1976).

[36] Wah, C., Branson, S., Welinder, P., Perona, P. & Belongie, S. The Caltech-UCSD Birds-200-2011 dataset. *Caltech Vision Lab* https://www.vision.caltech.edu/datasets/cub_200_2011/ (2011).

[37] Nilsback, M.-E. & Zisserman, A. Automated flower classification over a large number of classes. In *Proc.**Sixth Indian Conference on Computer Vision, Graphics & Image Processing* 722–729 (IEEE, 2008).

[38] Deng, J. et al. ImageNet: a large-scale hierarchical image database. In *Proc.**2009 IEEE Conference on Computer Vision and Pattern Recognition* 248–255 (IEEE, 2009).

[39] Zhou, B., Lapedriza, A., Khosla, A., Oliva, A. & Torralba, A. Places: a 10 million image database for scene recognition. *IEEE Trans. Pattern Anal. Mach. Intell.* **40**, 1452–1464 (2018). 28692961 10.1109/TPAMI.2017.2723009

[40] Sugiyama, M. & Kawanabe, M. *Machine Learning in Non-stationary Environments: Introduction to Covariate Shift Adaptation* (MIT Press, 2012).

[41] Farahani, A., Voghoei, S., Rasheed, K. & Arabnia, H. R. in *Advances in Data Science and Information Engineering: Proceedings from ICDATA 2020 and IKE 2020* (eds Stahlbock, R. et al.) 877–894 (Springer, 2021).

[42] Santurkar, S., Tsipras, D. & Madry, A. BREEDS: benchmarks for subpopulation shift. In *Proc. The Ninth International Conference on Learning Representations* (ICLR, 2021).

[43] Hendrycks, D., Zhao, K., Basart, S., Steinhardt, J. & Song, D. Natural adversarial examples. In *Proc. IEEE/CVF Conference on Computer Vision and Pattern Recognition* 15262–15271 (IEEE, 2021).

[44] Kriegeskorte, N., Mur, M. & Bandettini, P. A. Representational similarity analysis-connecting the branches of systems neuroscience. *Front. Syst. Neurosci.* **2**, 4 (2008). 10.3389/neuro.06.004.2008 PMC2605405 19104670

[45] Sucholutsky, I. et al. Getting aligned on representational alignment. Preprint at https://arxiv.org/abs/2310.13018 (2023).

[46] Gary, F. Marcus. Rethinking eliminative connectionism. *Cogn. Psychol.* **37**, 243–282 (1998). 9892549 10.1006/cogp.1998.0694

[47] Holyoak, K. J. & Hummel, J. E. in *Cognitive Dynamics* (eds Dietrich, E. & Markman, A. B.) 229–263 (Psychology Press, 2014).

[48] Geiger, A., Carstensen, A., Frank, M. C. & Potts, C. Relational reasoning and generalization using nonsymbolic neural networks. *Psychol. Rev.* **130**, 308 (2023). 35834185 10.1037/rev0000371

[49] Paleyes, A., Urma, R.-G. & Lawrence, N. D. Challenges in deploying machine learning: a survey of case studies. *ACM Comput. Surv.* **55**, 1–29 (2022).

[50] Amodei, D. et al. Concrete problems in AI safety. Preprint at https://arxiv.org/abs/1606.06565 (2016).

[51] Hebart, M. N., et al. THINGS-data, a multimodal collection of large-scale datasets for investigating object representations in human brain and behavior. *eLife* **12**, e82580 (2023). 36847339 10.7554/eLife.82580 PMC10038662

[52] Fukuzawa, K., et al. Internal representations and the conceptual operation of color in pure alexia with color naming defects. *Brain Lang.* **34**, 98–126 (1988). 3382935 10.1016/0093-934x(88)90126-5

[53] Robilotto, R. & Zaidi, Q. Limits of lightness identification for real objects under natural viewing conditions. *J. Vis.* **4**, 779–797 (2004). 15493970 10.1167/4.9.9

[54] Hebart, M. N., Zheng, C. Y., Pereira, F. & Baker, C. I. Revealing the multidimensional mental representations of natural objects underlying humansimilarity judgements. *Nat. Hum. Behav.* **4**, 1173–1185 (2020). 33046861 10.1038/s41562-020-00951-3 PMC7666026

[55] Muttenthaler, L. et al. VICE: variational interpretable concept embeddings. *Adv. Neural Inf. Process. Syst.* **35**, 33661–33675 (2022).

[56] Kingma, D. P. & Welling, M. Auto-encoding variational Bayes. In* Proc. The Second International Conference on Learning Representations* (eds Bengio, Y. & LeCun, Y.) (ICLR, 2014).

[57] Jimenez Rezende, D., Mohamed, S. & Wierstra, D. Stochastic backpropagation and approximate inference in deep generative models. In *Proc. 31st International Conference on Machine Learning* Vol. 32 (eds Xing, E. P. & Jebara, T.) 1278–1286 (PMLR, 2014).

[58] Graves, A. Practical variational inference for neural networks. In *Proc. Advances in Neural Information Processing Systems* Vol. 24 (eds Shawe-Taylor, J. et al.) 2348–2356 (Curran Associates, 2011).

[59] Blundell, C., Cornebise, J., Kavukcuoglu, K. & Wierstra, D. Weight uncertainty in neural network. In *Proc. 32nd International Conference on Machine Learning* Vol. 37 (eds Bach, F. & Blei, D.) 1613–1622 (PMLR, 2015).

[60] Blei, D. M., Kucukelbir, A. & McAuliffe, J. D. Variational inference: a review for statisticians. *J. Am. Stat. Assoc.* **112**, 859–877 (2017).

[61] Muttenthaler, L. et al. Improving neural network representations using human similarity judgments. In* Proc. Advances in Neural Information Processing Systems* Vol. 36 (eds Oh, A. et al.) 50978–51007 (Curran Associates, 2023).

[62] Muttenthaler, L., Vandermeulen, R. A., Zhang, Q., Unterthiner, T. & Müller, K.-R. Set learning for accurate and calibrated models. In *Proc.**The Twelfth International Conference on Learning Representations* (ICLR, 2024).

[63] Zhai, X., Mustafa, B., Kolesnikov, A. & Beyer, L. Sigmoid loss for language image pre-training. In *Proc. IEEE/CVF International Conference on Computer Vision* 11975–11986 (IEEE, 2023).

[64] Radford, A. et al. Learning transferable visual models from natural language supervision. In *Proc 38th International Conference on Machine Learning* Vol. 139 (eds Meila, M. & Zhang, T.) 8748–8763 (PMLR, 2021).

[65] Jia, C. et al. Scaling up visual and vision-language representation learning with noisy text supervision. In *Proc. 38th International Conference on Machine Learning* Vol. 139 (eds Meila, M. & Zhang, T.) 4904–4916 (PMLR, 2021).

[66] Alabdulmohsin, I. M., Zhai, X., Kolesnikov, A. & Beyer, L. Getting ViT in shape: scaling laws for compute-optimal model design. In *Proc. Advances in Neural Information Processing Systems* Vol. 36 (eds Oh, A. et al.) 16406–16425 (Curran Associates, 2023).

[67] Chen, X. et al. Pali-3 vision language models: smaller, faster, stronger. Preprint at https://arxiv.org/abs/2310.09199 (2023).

[68] Beyer, L. et al. PaliGemma: aversatile 3B VLM for transfer. Preprint at https://arxiv.org/abs/2407.07726 (2024).

[69] Caron, M. et al. Emerging properties in self-supervised vision transformers. In *Proc. IEEE/CVF International Conference on Computer Vision* 9650–9660 (IEEE, 2021).

[70] Tschannen, M. et al. SigLIP 2: multilingual vision-language encoders with improved semantic understanding, localization, and dense features. Preprint at https://arxiv.org/abs/2502.14786 (2025).

[71] Cichy, R. M., Kriegeskorte, N., Jozwik, K. M., van den Bosch, J. J. F. & Charest, I. The spatiotemporal neural dynamics underlying perceivedsimilarity for real-world objects. *NeuroImage* **194**, 12–24 (2019). 30894333 10.1016/j.neuroimage.2019.03.031 PMC6547050

[72] Peterson, J. C., Abbott, J. T. & Griffiths, T. L. Adapting deep network features to capture psychological representations. In *Proc. 38th Annual Meeting of the Cognitive Science Society, Recognizing and Representing Events* (eds Papafragou, A. et al.) (Cognitive Science Society, 2016).

[73] Peterson, J. C., Battleday, R. M., Griffiths, T. L. & Russakovsky, O. Human uncertainty makes classification more robust. In *Proc. IEEE/CVF International Conference on Computer Vision* 9616–9625 (IEEE, 2019).

[74] Brodeur, M. B., Guérard, K. & Bouras, M. Bank of standardized stimuli (BOSS) phase II: 930 new normative photos. *PLoS One* **9**, e106953 (2014). 25211489 10.1371/journal.pone.0106953 PMC4161371

[75] Grant, D. A. The Latin square principle in the design and analysis of psychological experiments. *Psychol. Bull.* **45**, 427 (1948). 10.1037/h0053912 18885731

[76] Born, F. Frieda-Josefine/HumanEval_experiment: HumanEval experiment. *Zenodo*10.5281/zenodo.13749102 (2025).

[77] Born, F. AligNet Human-Validation-Experiment. *Zenodo*10.5281/zenodo.15554173 (2025).

