# Supplementary material for: Aligning machine and human visual representations across abstraction levels
Source: Nature. 2025 Nov 12;647(8089):349–55. doi: 10.1038/s41586-025-09631-6 (PMC12611773; doi:10.1038/s41586-025-09631-6)
Supplement: Supplementary file 1 — Supplementary Notes, including the detailed results of statistical models, and other analysis details (9 tables and 15 figures). [file 41586_2025_9631_MOESM1_ESM.pdf]

nature portfolio

https://doi.org/10.1038/s41586-025-09631-6

Supplementary information

Aligning machine and human visual representations across abstraction levels

In the format provided by the authors and unedited

Supplementary Material

A. Additional/Detailed Results

In addition to the results that we presented in the main paper, here, we report the same set of results in more detail and add further analyses that we touched upon in the main part but did not go into much detail. We start by presenting findings from a comprehensive set of evaluations that we performed to test the alignment of model representations with human similarity judgments both for similarity judgments collected in previous efforts [cf. 1–4] and for our own similarity judgment dataset Levels (see Methods). Subsequently, we demonstrate benefits of our method for machine learning downstream tasks that test generalization, such as *few-shot learning* and *out-of-distribution detection*. Finally, we perform a rigorous qualitative analysis of the changes in the model representations after applying our alignment framework to the various student models.

A.1. Human Alignment

In this section, we present additional evaluation results regarding the alignment of model representations with human similarity judgments, complementing Sec. 2.1. For every student vision foundation model, we consider four settings of model representations and their refinements,

- *Original*: We use the pretrained representations of every student model without any changes to their representation space.
- *Distillation without alignment (UnAligNet)*: We perform AligNet fine-tuning without the first step (see Fig. 1) of aligning the teacher model with the human similarity judgments in THINGS. That is, we distill the teacher similarity structure of the pretrained, non-aligned SigLIP-So400m model (see Methods) into a student VFM.
- *Uncertainty Distillation (UD)*: We learn a linear transform of the models' pretrained representation space into a human global object similarity space (using the THINGS triplet odd-one-out choices) while preserving the model's local similarity structure (see

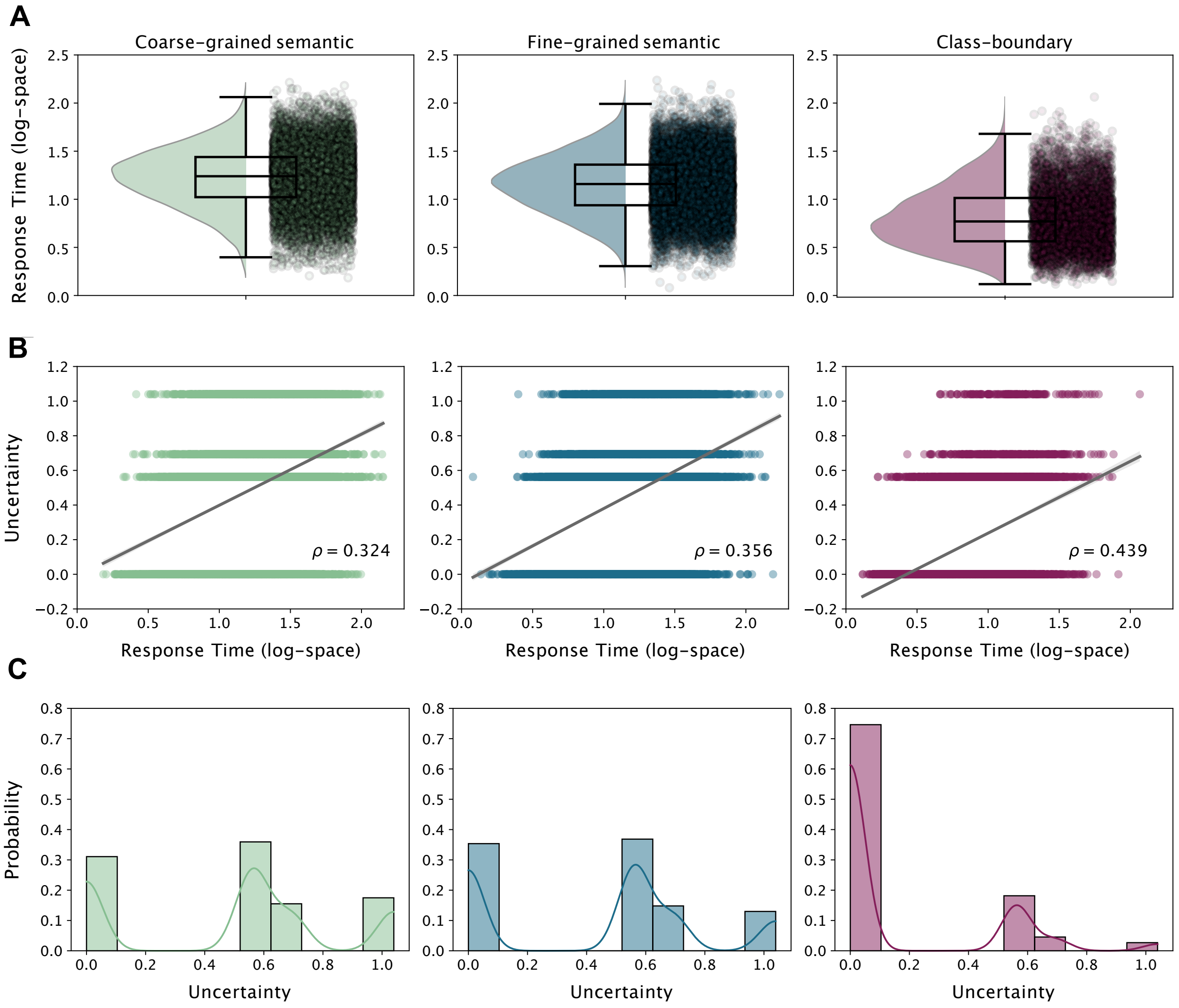

Figure 1 | Human response times and disagreement levels for the different triplet types. **A**: Distribution of human response times (in log-space) for the three different triplet type settings. **B**: Spearman rank correlation and linear regression fit of the participant disagreement levels/uncertainties with the participants' response times (in log-space). **C**: Probability distributions of the participant disagreement levels/uncertainties for the three triplet type settings.

Eq. 4). We remark that this does not involve any step of the AligNet framework. The weights of the student models are frozen during the UD optimization.

- *AligNet/Soft-alignment*: We learn a new representation space using the full AligNet framework as outlined in the Methods. This can be referred to as *soft-alignment*.

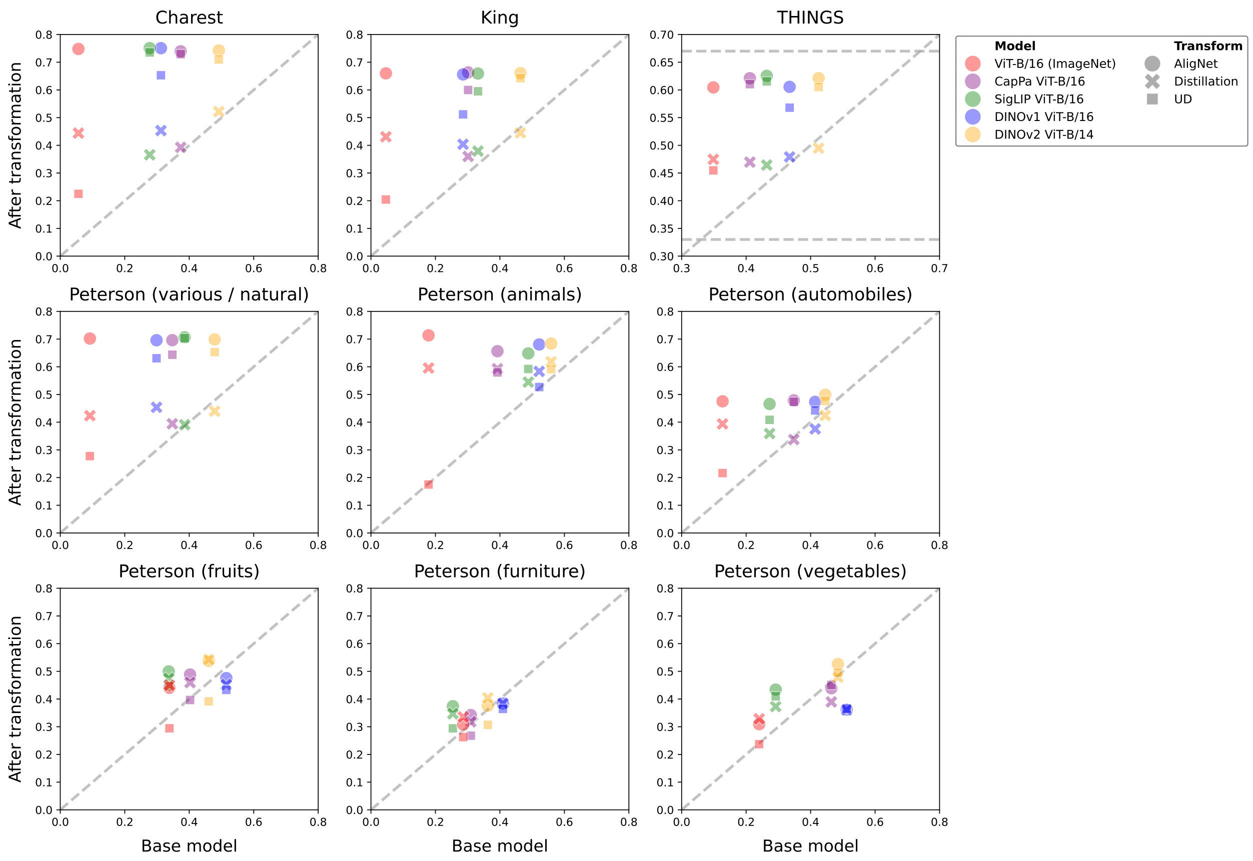

Figure 2 | Alignment of model representations with human similarity judgments for the four different representation settings *Original*, *UnAlignNet*, *UD*, and *AligNet* for all models with a ViT-B backbone. The x-axis shows the degree of alignment for the base model, i.e., the *original* representations, whereas the y-axis displays the degree of alignment after linearly transforming the representations via UD or fine-tuning the models using distillation (without alignment) or the full AligNet framework. For all datasets but THINGS alignment is measured as the Spearman rank correlation coefficient between human and model RSMs. For THINGS, alignment is measured as triplet odd-one-out accuracy, i.e., the fraction of triplets for which models selected the same odd-one-out object as the human participants. Dashed horizontal lines for THINGS depict random guessing (0.333) and the human noise-ceiling/reliability score (0.667) respectively.

A.1.1. Detailed results for RSA

In Tab. 1 we present the average alignment scores for all student models that we considered in our analyses (see Methods) and each of the four representation settings defined above for the datasets from Cichy et al. [2], King et al. [3], and Hebart et al. [4]. For the former two datasets, alignment is measured via RSA using Spearman rank correlation between the upper triangulars of the pairwise similarity matrices. For those datasets, human similarity judgments were collected using a multi-arrangment task where participants were asked to

arrange (natural) objects on a computer screen (see Methods for details). For the THINGS dataset, alignment is measured via triplet odd-one-out accuracy, i.e., the fraction of triplets for which models selected the same odd-one-out object as the human participants.

Model \ Fine-tuning	Cichy et al. [2]				King et al. [3]				Hebart et al. [4]			
	Original	UnAligNet	UD	AligNet	Original	UnAligNet	UD	AligNet	Original	UnAligNet	UD	AligNet
ViT-S	0.083	0.520	0.367	0.743	0.143	**0.486**	0.353	**0.665**†	39.28%	**49.97**%	50.01%	59.44%
ViT-B	0.056	0.444	0.225	0.748	0.046	0.431	0.204	0.660	34.92%	47.47%	45.47%	60.45%
ViT-L	0.041	**0.540**	0.227	**0.751**†	0.095	0.463	0.201	0.664	34.84%	49.30%	44.40%	60.75%
CapPa ViT-B	0.373	0.393	0.729	0.739	0.301	0.360	0.599	0.663	40.58%	46.97%	61.04%	62.11%
DINOv1 ViT-B	0.312	0.453	0.653	**0.751**†	0.286	0.404	0.512	0.656	46.75%	47.91%	56.80%	60.56%
DINOv2 ViT-B	**0.492**	0.522	0.710	0.743	**0.464**	0.445	_	0.661	**51.24**%	49.49%	60.51%	62.13%
SigLIP ViT-B	0.277	0.366	**0.735**	**0.751**†	0.332	0.379	0.595	0.659	43.20%	46.44%	61.53%	**62.54**%†
SigLIP So400m (Teacher)	0.226	_	0.723	_	0.267	_	**0.638**	_	44.24%	_	**61.70**%	_

Table 1 | Human alignment results for three different human similarity judgment datasets. The datasets from Cichy et al. [2] and King et al. [3] were collected using a multi-arrangement task. For those datasets, alignment with human similarity judgments is measured via RSA using the Spearman rank correlation. The dataset from Hebart et al. [4] used a triplet odd-one-out task for collecting human judgments. Here, alignment with human choices is measured via triplet odd-one-out accuracy (%). Bold face indicates highest performance within a single column and † indicates best performance for a dataset overall.

We find that base models—that is the *original* representation setting (see above)—performed poorly for almost every human similarity judgment dataset (see Tab. 1 and Fig. 2). The differences among the models in this setting is significant. While DINOv2 ViT-B achieves a Spearman rank correlation coefficient of $\rho = 0.492$, $p < 0.001$ for the dataset in Cichy et al. [2] and an odd-one-out accuracy of 51.21% for the THINGS dataset, ViT-L is not correlated with the human similarity judgments from Cichy et al. [2] and King et al. [3] ($\rho = 0.041$ and $\rho = 0.095$ respectively) and shows a close to chance-level odd-one-out accuracy of 34.84% for the THINGS dataset (see Tab. 1 and x-axis in Fig. 3). AligNet fine-tuning significantly improved the alignment scores of all models for all datasets. In addition, it minimized the differences between the models to an extent that their differences in the degree of alignment are not statistically significant. Thus, AligNet fine-tuning models are equally well aligned with human similarity judgments irrespective of their architecture, pretraining data, and objective function.

In the top row of Fig. 2 we show the performance of the transformed representations as a function of the base model performance for those datasets. We can see that AligNet

fine-tuning significantly improves upon the base model representations (x-axis). It yields the most human-aligned models across all three datasets (depicted by the circles) and often substantially improves upon the linearly aligned representations (depicted by the squares).

Model \ Fine-tuning	Peterson et al. [1, 5] (coarse-grained) Original	UnAligNet	UD	AligNet	Peterson et al. [1, 5] (fine-grained) Original	UnAligNet	UD	AligNet
ViT-S	0.249	**0.454**	0.379	0.663	0.330	0.461	0.320	0.459
ViT-B	0.092	0.423	0.278	0.702	0.234	0.421	0.237	0.449
ViT-L	0.118	0.448	0.231	0.704	0.221	0.421	0.234	0.447
CapPa ViT-B	0.347	0.394	0.644	0.696	0.383	0.419	0.434	0.481
DINOv1 ViT-B	0.299	**0.454**	0.631	0.696	**0.474**	0.431	0.426	0.475
DINOv2 ViT-B	**0.479**	0.440	0.653	0.699	0.462	**0.494**	**0.452**	**0.524**†
SigLIP ViT-B	0.386	0.391	**0.703**	**0.707**†	0.329	0.419	0.430	0.484

Table 2 | Human alignment results for the dataset from Peterson et al. [1, 5]. Human pairwise similarity judgments were collected using an ordinal Likert scale. Scores reflect the average Spearman rank correlation coefficients with the human pairwise similarity judgments. To obtain an average alignment score for the fine-grained setting, we averaged the performances across the five single category settings: `animals`, `automobiles`, `fruits`, `furniture`, and `vegetables`. Bold face indicates highest performance within a single column and † indicates best performance for a dataset overall.

Human similarity judgments from the dataset introduced in Peterson et al. [1, 5] were collected using an ordinal Likert scale (see Methods for details). The authors collected pairwise similarity ratings for `various` natural objects with one image per category, similar to the other three human similarity judgment datasets. Thus, those pairwise similarity ratings reflect global coarse-grained semantic structure. In addition, the authors collected pairwise similarity ratings for multiple images from a single category that reflect local fine-grained semantics. The specific categories for which similarity ratings were collected are `animals`, `automobiles`, `fruits`, `furniture`, and `vegetables`. We average model performances across those five single category pairwise similarity rating datasets to obtain a single measure for fine-grained semantic and refer to it as the fine-grained dataset of Peterson et al. [1, 5]. In Tab. 2, we report Spearman rank correlation coefficients of the model RSMs with the RSMs obtained from the human similarity ratings for all student models and the four representation settings. AligNet fine-tuning significantly improves performance across the board, even for the fine-grained setting. While the best model for the coarse-grained setting is AligNet fine-tuned SigLIP ViT-B ($\rho = 0.707, p < 0.001$), AligNet fine-tuned DINOv2 ViT-B is most

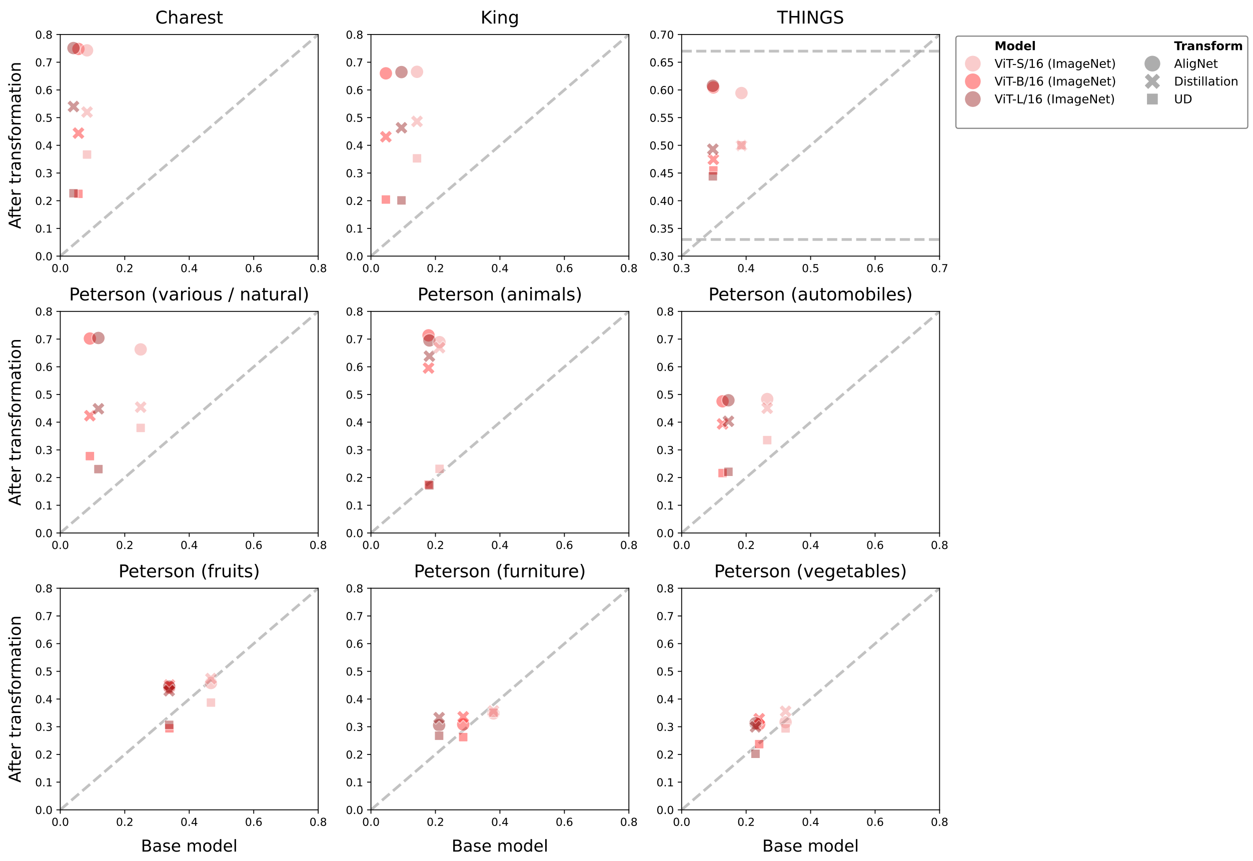

Figure 3 | Alignment of model representations with human similarity judgments for the four different representation settings *Original*, *UnAligNet*, *UD*, and *AligNet* for the three supervised ImageNet-trained ViT models. The x-axis shows the degree of alignment for the base model, i.e., the *original* representations, and the y-axis displays the degree of alignment after linearly transforming the representations via UD or fine-tuning the models using distillation (without alignment) or the full AligNet framework. For all datasets but THINGS alignment is measured as the Spearman rank correlation coefficient between human and model RSMs. For THINGS, alignment is measured as triplet odd-one-out accuracy, i.e., the fraction of triplets for which models selected the same odd-one-out object as the human participants. Dashed horizontal lines for THINGS depict random guessing (0.333) and the human noise-ceiling (0.667) respectively.

human-aligned model for the fine-grained setting ($\rho = 0.524$, $p < 0.001$). AligNet fine-tuning significantly improves the Spearman rank correlation coefficients for all models compared to their original representations in the coarse-grained setting.

In the bottom two rows of Fig. 2 and Fig. 3 respectively we show model performances individually for the single category pairwise similarity rating datasets. We observe that AligNet fine-tuning significantly improved the degree of alignment for all models for the `various`, `animals`, and `automobiles` categories but there does not appear to be a differ-

ence in performance between the different model transformation settings for the categories `fruits`, `furniture`, and `vegetables`. We hypothesize that this difference in alignment benefits stems from the fact that animal- and automobile-related concepts are more frequently represented in the THINGS dataset than fruits-, furniture-, and vegetable-related concepts [cf. 4, 6]. Moreover, THINGS is a human similarity judgment dataset that reflects global coarse-grained semantic, similar to the coarse-grained setting in Levels. Thus, it is rather surprising to find benefits for human local fine-grained semantic by either UD or fine-tuning on AligNet.

A.1.2. Larger models benefit more from AligNet

In Fig. 3 we compare supervised ImageNet-trained ViTs of different sizes. Interestingly, the smallest version of the ViTs, ViT-S, achieved the best base model performance across the board. That is, their original representation space was better aligned with the different human similarity judgments than the representation spaces of the larger ViT backbones (see Fig. 3 and Tables 1, 2). However, after fine-tuning the models on AligNet the larger ViT versions were either better or equally-well aligned than ViT-S.

A.1.3. Descriptive statistics for human response times and uncertainty measures on Levels

We examined the human response time (RT) differences across the three abstraction settings as an indicator of cognitive processing demands during odd-one-out selection (see Fig. 1A). As expected, responses were fastest for the class-boundary triplets (Mdn = 2.04 s, SE = 0.03), significantly slower for fine-grained semantic triplets (Mdn = 3.07 s, SE = 0.05; $t(447) = 45.920$, $p < 0.001$), and slowest for coarse-grained semantic triplets (Mdn = 3.25 s, SE = 0.06; $t(447) = 14.160$, $p < 0.001$ compared to fine-grained). Unlike the simpler choices enabled by clear semantic boundaries, longer RTs at higher levels of semantic abstraction likely reflected more complex decisions, where participants may have weighed information across multiple perceptual and semantic dimensions. Consistent with these RT results, we

also found higher levels of cross-participant agreement (see Fig. 1C) in the class boundary condition compared to both the coarse-grained semantic and the fine-grained semantic conditions.

Participants' RTs were used as a proxy for decision uncertainty. RTs also correlated positively with cross-participant disagreement within each abstraction levels (coarse-grained semantic: $r = 0.325$, $p < 0.001$; fine-grained semantic: $r = 0.364$, $p < 0.001$; class-boundary: $r = 0.443$, $p < 0.001$; see Methods and Fig. 1B), suggesting that human RTs provided a reasonable approximation of decision uncertainty for model-to-human comparison.

A.1.4. Detailed results for Levels

In Tab. 3 we present triplet odd-one-out accuracies for every model that we considered in our analyses for each of the three levels of abstraction in the Levels dataset.

Global coarse-grained. In this setting, AligNet fine-tuning improved alignment with human similarity judgments most significantly. Before fine-tuning, models achieved poor odd-one-out accuracies of 35.45% (ViT-L) – 57.38% (DINOv2 ViT-B). Fine-tuning models on AligNet closed this gap and significantly improved the alignment between human and model responses to the extent that all models performed with odd-one-out accuracies of 65.70% (DINOv1 ViT-B) – 68.60% (ViT-L) above the human-to-human reliability score of 61.92%. Interestingly, the most poorly performing model before fine-tuning, ViT-L, achieved the highest odd-one-out accuracy after fine-tuning on AligNet, with a relative improvement of almost 93.51%. Even the worst performing AligNet-tuned model, DINOv1 ViT-B, performed with an odd-one-out accuracy of 65.70% significantly better than the best performing base model, DINOv2 ViT-B with 57.38%, and better than the best performing model after vanilla distillation without alignment, ViT-S with 58.82%, (see the coarse-grained column in Tab. 3). The relative performance improvements ranged from 19.48% (DINOv2 ViT-B) – 93.51% (ViT-L). Three models—SigLIP ViT-B, DINOv2 ViT-B, and ViT-L—performed even better than the teacher model which is the strongest baseline in that setting that exists at the time of

writing this manuscript.

Local fine-grained. The responses of most base models' did not strongly correspond to human responses for fine-grained semantics either and, thus, model performances were far from the human reliabiltiy score. The human noise-ceiling was with 65.92% similar to the noise-ceiling of the coarse-grained setting. Prior to fine-tuning, models achieved poor alignment scores of 40.75% (ViT-L) – 57.72% (DINOv2 ViT-B), except for DINOv1 ViT-B whose base model performance (62.92%) was significantly better than the performance of the other models (see Tab 3).

Soft-alignment improved this mismatch to some degree but not as significantly as it did for the coarse-grained abstraction setting. AligNet models achieved odd-one-out accuracies of 58.93 (ViT-S) – 62.92% (DINOv1 ViT-B) (see the fine-grained column in Tab. 3). Here, relative increases in performance ranged from 7.84% (DINOv2 ViT-B) - 46.03% (ViT-L). For a single model, DINOv1 ViT-B, the alignment score did not change after soft-alignment. Again, the best performing soft-aligned model, DINOv1 ViT-B, performed with an odd-one-out accuracy of 62.92% better than any other model—whether linearly aligned using UD or fine-tuned via teacher distillation—in that setting. Note that UD and fine-tuning via distillation decreased the performance of DINOv1 ViT-B. This model performed closest to the human reliability score of 65.92%. Every soft-aligned model performed better than the teacher in that abstraction setting.

It is rather surprising that AligNet models were better aligned than their base models for this setting because the THINGS triplets reflect coarse-grained semantic structure and, hence, neither teacher nor student representations have ever explicitly learned anything about a fine-grained human object similarity space. We hypothesize that the fusion of information about human coarse-grained semantic and the teacher model's local similarity structure leads to a representation space that better reflects human object similarity structure in general—irrespective of the level of granularity.

Class-boundary. Supervised classifiers and image/text contrastive models performed close

to the noise ceiling for class-boundary triplets prior to any fine-tuning. Their odd-one-out accuracies ranged from 81.96% (SigLIP ViT-B) to 93.67% (ViT-L) (see Tab. 3). However, the caption generator model (CapPa) often responded differently from the human participants in that setting and achieved a significantly lower odd-one-out accuracy score compared to the other student models. It selected the same odd-one-out image as the human participants in 70.37% of the triplets.

AligNet fine-tuning changed the representation spaces of all models to be equally well aligned with the human respondents. The differences between AligNet models was significantly smaller compared to the other settings, with odd-one-out accuracies of 93.09% – 94.24%. Surprisingly, the odd-one-out accuracies of models fine-tuned on AligNet were higher than the human reliability score of 89.21% (see Fig. 2D, rightmost column) with the best model achieving an impressive alignment score of 94.24% (ViT-L). This means that the responses of AligNet models were more similar to the average human responses—since each triplet response is the majority response of the subject population—than the level of agreement among the human subjects themselves. The performances of all models improved by fine-tuning them on AligNet. The relative increases in performance were between 0.62% – 32.39%.

Simply performing *distillation without alignment* did not improve the models' alignment with humans as significantly. For some models (e.g., ViT-L) simply distilling the similarity structure of the teacher model into the student without aligning the teacher's representations even decreased the degree of alignment with the human odd-one-out responses (see the class-boundary column in Tab. 3). Similarly, applying the UD transformation to a model's representation space sometimes slightly improved the human-model fit (e.g. ViT-B) and sometimes significantly decreased model performance (e.g. ViT-S) with large differences between models. Moreover, AligNet fine-tuning was the only transformation technique that could significantly decrease the variance in performance across the different student models. The difference between the worst (CapPa ViT-B) and the best (ViT-L) AligNet fine-tuned

model was just 1.15%

Model \ Fine-tuning	Coarse-grained				Fine-grained				Class-boundary			
	Original	UnAligNet	UD	AligNet	Original	UnAligNet	UD	AligNet	Original	UnAligNet	UD	AligNet
ViT-S	40.08%	**58.82%**	46.29%	67.09%	51.30%	58.80%	43.41%	58.93%	92.23%	92.64%	85.31%	93.80%
ViT-B	36.10%	56.15%	40.96%	67.72%	46.04%	57.04%	42.66%	60.02%	88.73%	91.68%	**93.31%**	93.99%
ViT-L	35.45%	57.75%	41.84%	**68.60%**†	40.75%	57.47%	39.34%	59.51%	**93.67%**	**93.18%**	**93.31%**	**94.24%**†
CapPa-B ViT-B	42.99%	53.55%	66.73%	66.06%	49.36%	57.15%	53.07%	60.21%	70.37%	90.85%	87.92%	93.09%
DINOv1 ViT-B	52.44%	55.86%	57.82%	65.70%	**62.92%**†	**59.76%**	**60.91%**	**62.92%**†	90.09%	92.39%	88.84%	94.03%
DINOv2 ViT-B	**57.38%**	49.37%	66.09%	68.56%	57.72%	55.31%	55.72%	62.24%	89.74%	88.46%	90.13%	93.45%
SigLIP ViT-B	46.88%	51.72%	**68.17%**	68.47%	53.44%	57.39%	57.71%	60.94%	81.96%	90.81%	91.32%	93.75%
SigLIP So400m (Teacher)	50.24%	_	68.03%	_	57.27%	_	58.86%	_	90.42%	_	93.11%	_

Table 3 | Human alignment results for Levels. Here, we show triplet odd-one-out accuracies (in %)—measured as the fraction of triplets for which models selected the same odd-one-out image as the majority of the human participants for the three levels of abstraction—*coarse-grained*, *fine-grained*, and *class-boundary*—in the Levels dataset that we collected via online crowdsourcing. Bold face indicates highest performance within a single column and † indicates best performance for a triplet type setting overall.

A.1.5. Human alignment depends on the abstraction level

We found that the best model before soft-alignment at a particular abstraction level was often not the best model after soft-alignment. While DINOv2 was the best aligned model before AligNet fine-tuning for the fine-grained (57.38%) and coarse-grained (57.72%) abstraction settings (see Tab. 3), it remained the best aligned model only for the fine-grained setting (62.24%) but did not improve as significantly as the other models for the coarse-grained (relative improvement of 18.78%) and class-boundary settings (relative improvement of 4.13%). On the other hand, ViT-L was the worst-aligned model before fine-tuning for the coarse-grained setting (35.45%), but after AligNet fine-tuning, it became the best-aligned model, achieving the highest odd-one-out accuracy (68.60%) across all models. The relative improvement in performance was 93.51%. In addition, ViT-L was both the best aligned model before (93.67%) and after (94.24%) AligNet fine-tuning for the class-boundary setting (both scores are higher than the human reliability score of 89.21%).

We observed a similar phenomenon for the coarse- and fine-grained human responses datasets of Peterson et al. [1, 5]. DINOv2 showed the highest Spearman rank correlation coefficient with the human similarity judgments prior to any fine-tuning for both abstraction levels but remained the best aligned model only for the fine-grained datasets after fine-tuning

(see Tab. 2 for details). This suggests that DINOv2 fine-tuned on AligNet best captured fine-grained human semantic structure whereas image/text contrastive and supervised models best reflected coarse-grained and class-boundary semantic structure respectively after the fine-tuning process.

A.1.6. ImageNet vs. Ecoset models on Levels

Here, we evaluate vision models trained on Ecoset [7] on the Levels data. Ecoset is an ecologically motivated natural image dataset, designed in the hope to produce models that better reflect human perception than models trained on ImageNet. The number of (training) data points is the same between Ecoset and ImageNet (approx. 1.3M) but the number of classes is differs between the two datasets: 565 (Ecoset) vs. 1000 (ImageNet). We find that models trained on Ecoset are worse aligned with human similiarty judgments than the same set of models trained on ImageNet in most abstraction settings (see Tab. 4). This is in line with previous findings of Muttenthaler et al. [8] who found that Ecoset models are worse aligned than ImageNet models with the human similarity judgment datasets from Hebart et al. [4], King et al. [3], and Cichy et al. [2]. The reason for this is most likely the higher number of classes in ImageNet which has been found to play a crucial role for the alignment of neural network representations with human semantic cognition [8]. Note that Ecoset is designed for supervised learning, and weakly-/self-supervised image/text model representations generally correspond better to human behavior and brain data than the representations of supervised ImageNet models [8, 9].

A.1.7. Evaluating vision-language models on Levels

Although we focus on vision models in the main text, a growing area of research focuses on Vision-Language Models (VLMs). As language captures much of the detail of human semantic knowledge, it is natural to ask how these models perform on the Levels dataset. We therefore evaluated Gemini 2.0 Flash [10] and Gemini 2.5 Pro [11] on Levels, using a prompt that approximately follows the human instructions, except edited to describe a

Model \ Data	Coarse-grained Ecoset	ImageNet	Fine-grained Ecoset	ImageNet	Class-boundary Ecoset	ImageNet
AlexNet	42.83%	38.41%	44.39%	44.31%	55.47%	49.81%
VGG-16	42.65%	40.18%	45.16%	49.95%	60.34%	64.58%
ResNet-50	44.98%	46.46%	44.80%	49.98%	63.85%	91.90%
Inception v3	45.86%	36.08%	49.59%	35.76%	70.38%	82.28%

Table 4 | Human alignment results for Ecoset vs. ImageNet models on the Levels data. Here, we show triplet odd-one-out accuracies (in %)—measured as the fraction of triplets for which models selected the same odd-one-out image as the majority of the human participants for the three levels of abstraction—*coarse-grained*, *fine-grained*, and *class-boundary*—in the Levels dataset that we collected via online crowdsourcing.

single trial rather than a full experimental procedure: "I will show you three object images presented in sequence. These images show different things. Your task is to pick one image that is least similar to the other two. If you do not recognize what is shown to you in one of the images, simply base your judgement on your best guess at what the image might show. Which of these images is least similar to the others? Please give your answer ("first", "second", or "third") before explaining."

After this prompt, we presented the three images, and then evaluated the model's response. As expected, the models—and especially the stronger 2.5 Pro model—perform fairly well at the class-boundary judgments, and moderately well at the others; they are comparable to or better than the stronger vision models before alignment. However, neither VLM achieves the performance of AligNet, and they are especially weak on the coarse-grained judgments. These results suggest that the improvements offered by our methods are complementary to the benefits offered by the richer language supervision in VLMs.

Model \ Triplet type	Coarse-grained	Fine-grained	Class-boundary
Gemini 2.0 Flash	55.0%	58.8%	89.2%
Gemini 2.5 Pro	61.3%	61.3%	89.9%

Table 5 | Gemini 2.0 Flash and Gemini 2.5 Pro evaluation performance (odd-one-out accuracy) on the Levels dataset. The models perform well on most categories, comparable to the original versions of some of the stronger vision models—with the stronger model even outperforming them on coarse-grained triplets—but neither VLM achieves as high of performance as AligNet, especially on the coarse-grained triplets. (cf. Tab. 3.)

A.1.8. Correlation of model output uncertainties with human RTs

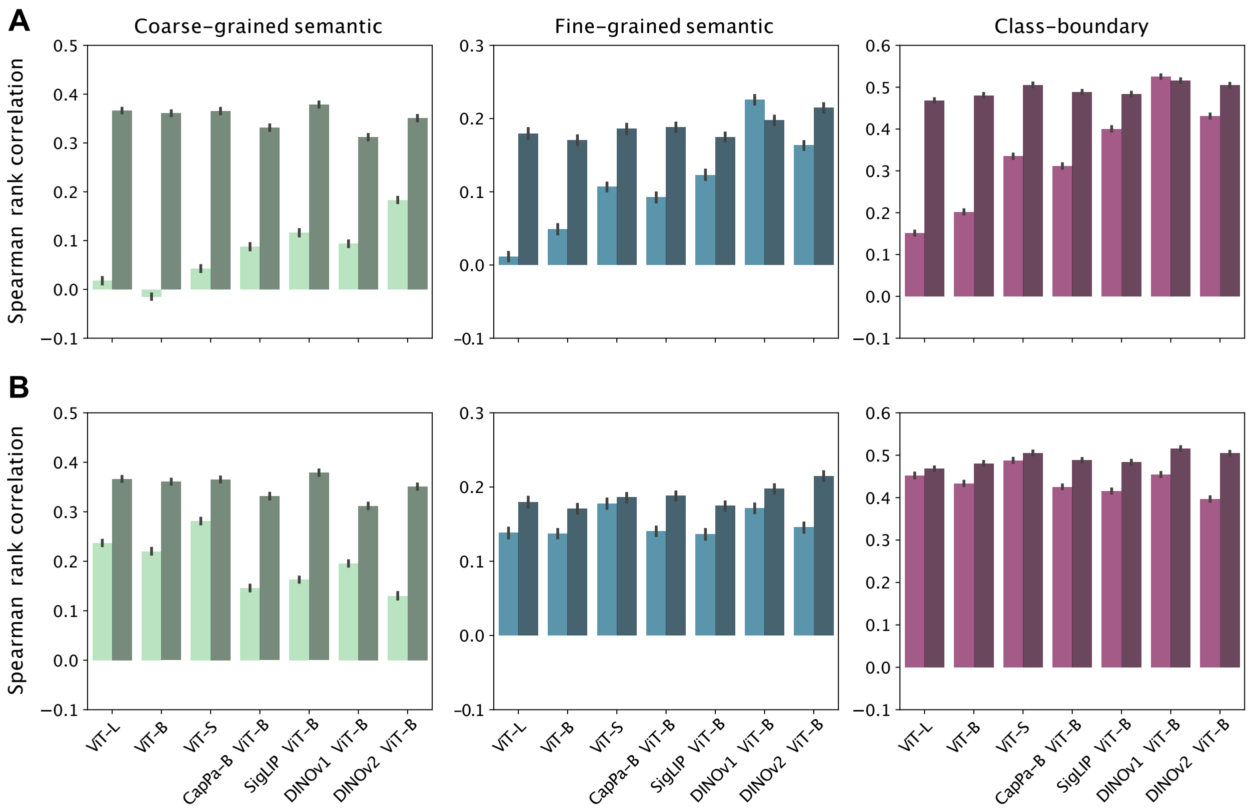

Figure 4 | Spearman rank correlation coefficients of model output uncertainties over the triplet odd-one-out choices (measured as discrete Shannon entropy) and the corresponding human response times (measured in log-space). The columns are partitioned into the three different triplet types: coarse-grained, fine-grained, and class-boundary. **A**: AligNet fine-tuning (darker colors) in contrast to the base model representations (lighter colors). **B**: AligNet fine-tuning (darker colors) in contrast to distillation without alignment (lighter colors). Error bars reflect the standard deviation (SD) on the item level over 1000 bootstraps.

Here, we evaluate how the uncertainties of the model outputs—measured as the discrete Shannon entropy of the triplet probabilities—correlate with the human response times for the different triplets. For all base models, we observed a poor (close to zero) Spearman rank correlation between its output uncertainties over the triplet odd-one-out choices and the human response times for both the global coarse-grained and the local fine-grained semantic settings (see panel A in Fig. 4). However, except for ViT-B and ViT-L, most base models showed a medium positive Spearman rank correlation (ρ=0.3-0.4) for the class-boundary setting (see rightmost column in panel A Fig. 4). Note that the class-boundary setting is the easiest setting with the least variance/disagreement among the human participants (see Methods

for details). AligNet-finetuning significantly improved the correspondence between model output uncertainties and human responses times for all models and triplet type settings. The improvements were most striking for the global coarse-grained semantic setting where every model achieved a medium to strong positive Spearman rank correlation of close to ρ=0.4. For the class-boundary setting, models fine-tuned on AligNet even achieved a strong positive Spearman rank correlation coefficient of close to ρ = 0.5.

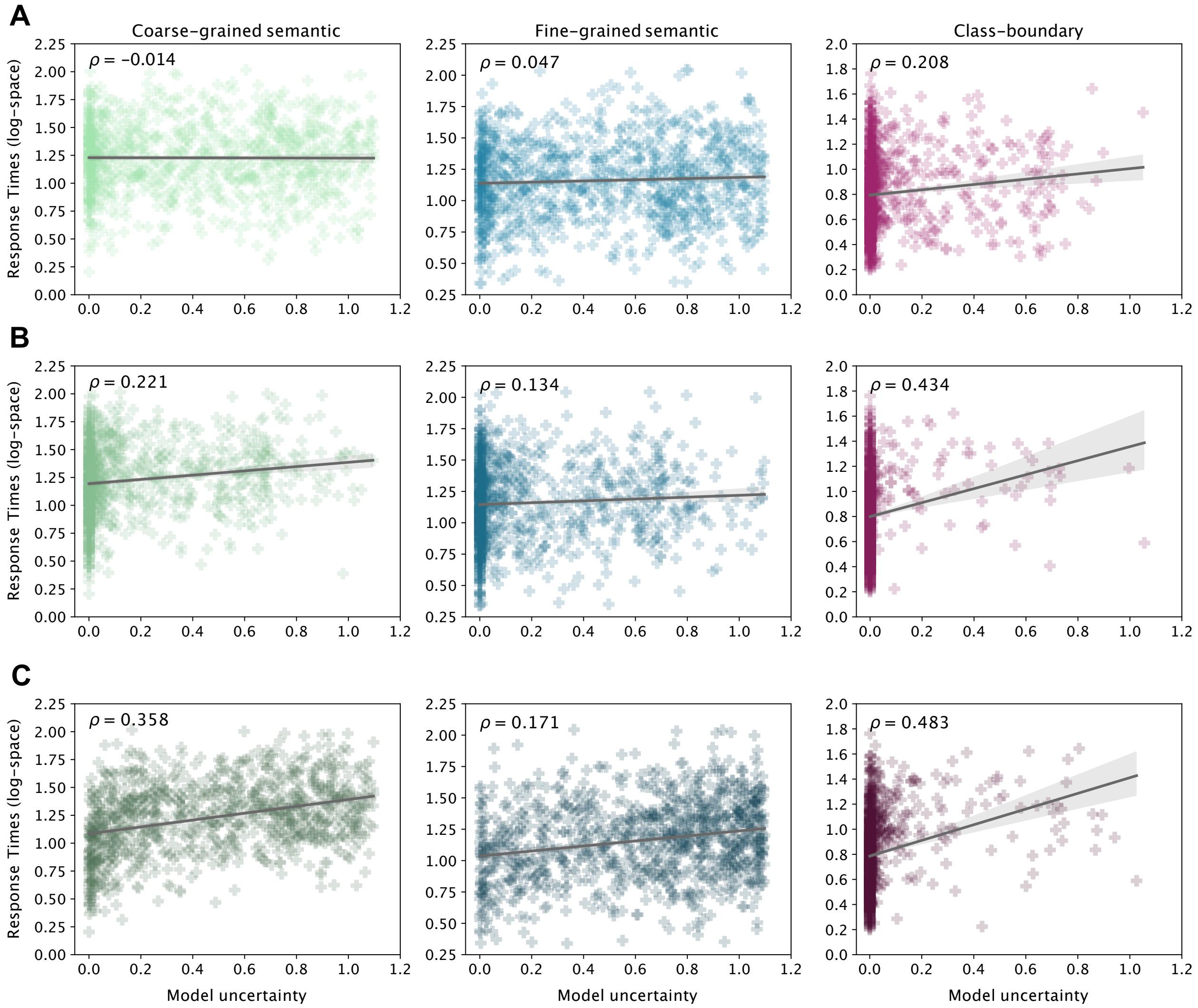

Figure 5 | Human response times (in log-space) as a function of ViT-B's output uncertainty over the triplet odd-one-out choices (measured as the discrete Shannon entropy). The columns are partitioned into the three different triplet types: coarse-grained, fine-grained, and class-boundary. **A**: Model uncertainties of *original* representations. **B**: Model uncertainties of *distilled* representations. **C**: Model uncertainties of *AligNet-finetuned* representations. Correlation coefficients are Spearman rank correlations.

In panel B of Fig. 4, we compare AligNet fine-tuned representations (darker colors) against

performing distillation without alignment (lighter colors). We can see that simply performing distillation without alignment could improve the correspondence of model uncertainties and human response times over the base representations but the improvement was not as substantial compared to applying the full AligNet framework. We find that for every model and every triplet setting, AligNet fine-tuning yielded a significantly stronger Spearman rank correlation coefficient. In Fig. 5, Fig. 6, and Fig. 7 we contrast the human response times (in log-space) against the model's output uncertainties over the triplet odd-one-out responses for ViT-B, DINOv2 ViT-B, and SigLIP ViT-B respectively for each triplet individually. We chose those three models because they reflect a representative subset of the student models (see Methods) that we fine-tuned on AligNet. Each of those models has the same backbone but was pretrained on a different datasets with a different objective function. We observe that AligNet fine-tuning significantly improved the correspondence of the models' output uncertainties to the human responses times for all three models and triplet settings. Interestingly, distillation without alignment, decreased the Spearman rank correlation coefficient of DINOv2 for each of the three triplet settings (see panel B in Fig. 6). That was not the case for ViT-B and SigLIP ViT-B (see panel B in Fig. 5 and Fig. 7 respectively).

Taken together, we find that AligNet fine-tuning significantly improved the correspondence of models' output uncertainties with the human response times irrespective of the task and objective with which a model was (pre-)trained and regardless of the specific triplet type. The improvements were most striking for global coarse-grained semantic.

A.2. Machine Learning

In this section we provide further details and evidence about the machine learning downstream task performances of our method. We start by demonstrating few-shot learning and out-of-distribution detection evaluations and end the section with showing various ablations.

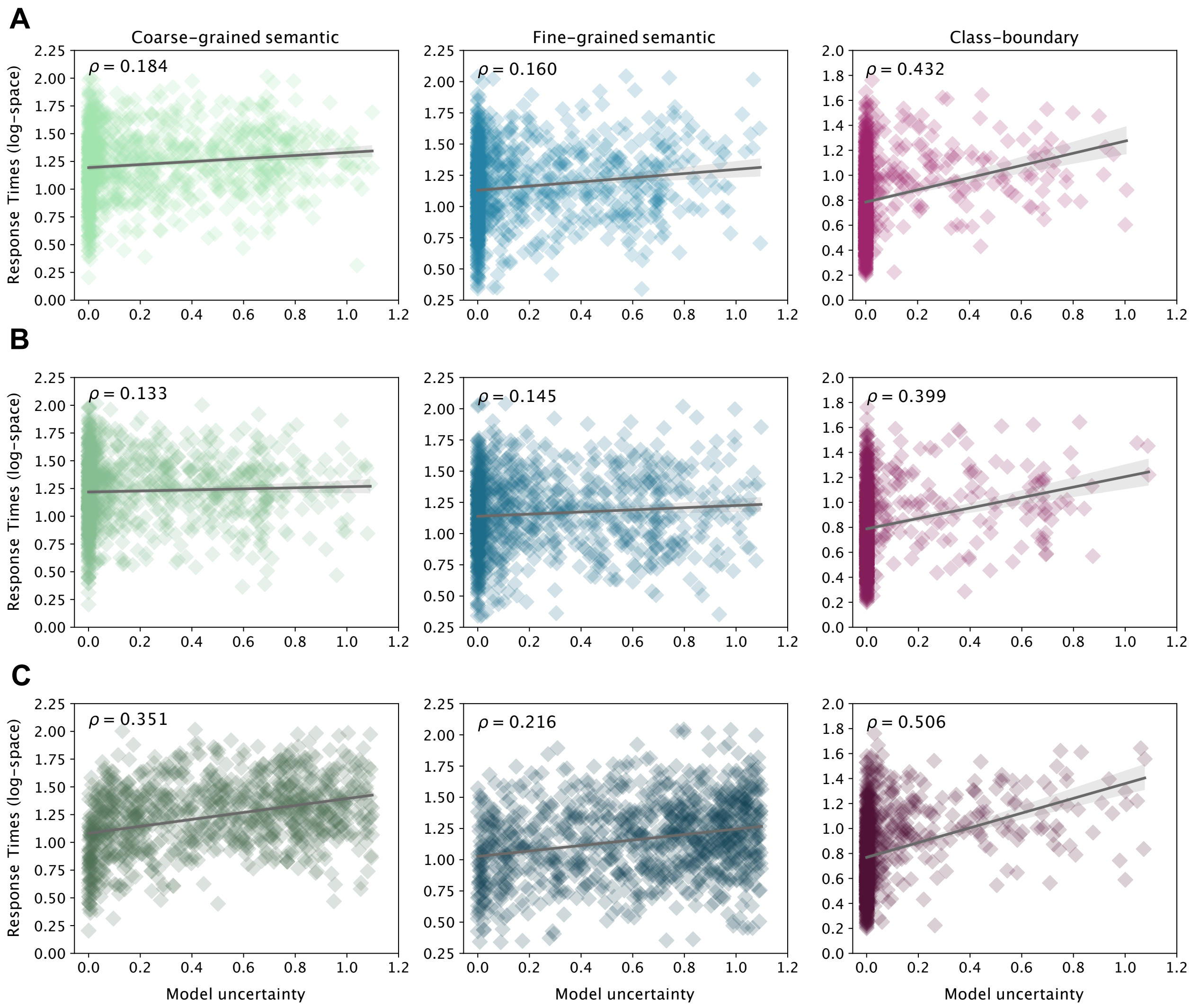

Figure 6 | Human response times (in log-space) as a function of DINOv2's output uncertainty over the triplet odd-one-out choices (measured as the discrete Shannon entropy). The columns are partitioned into the three different triplet types: coarse-grained, fine-grained, and class-boundary. **A**: Model uncertainties of *original* representations. **B**: Model uncertainties of *distilled* representations. **C**: Model uncertainties of *AligNet-finetuned* representations. Correlation coefficients are Spearman rank correlations.

A.2.1. Few-shot learning

Few-shot learning [12] is a common way to evaluate how well neural network representations generalize to new tasks: Here, the goal is to learn a new task/dataset from only a few labelled examples (typically ≤10 examples per labelled class are used to train a classifier on top of the pretrained representations), and then evaluate its performance on the full test set for that task. Good few-shot performance is indicative of models that have a solid understanding of

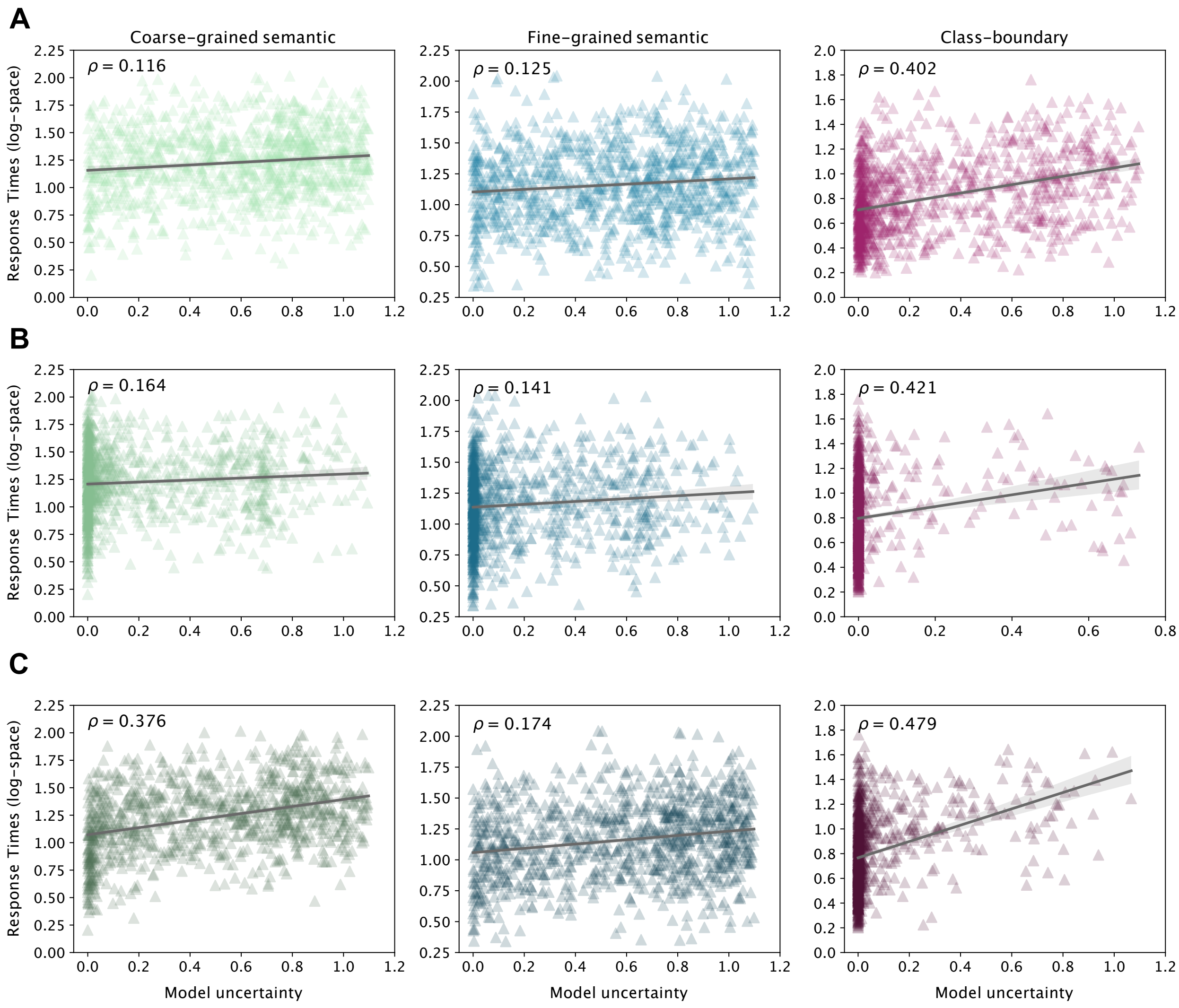

Figure 7 | Human response times (in log-space) as a function of SigLIP ViT-B's output uncertainty over the triplet odd-one-out choices (measured as the discrete Shannon entropy). The columns are partitioned into the three different triplet types: coarse-grained, fine-grained, and class-boundary. **A**: Model uncertainties of *original* representations. **B**: Model uncertainties of *distilled* representations. **C**: Model uncertainties of *AligNet-finetuned* representations. Correlation coefficients are Spearman rank correlations.

many different concepts, and that their representations are able to quickly integrate/expand to new or related concepts.

We run few-shot learning evaluations on eight different supervised learning tasks. ImageNet [13], Places365 [14] and Cifar100 [15] test for broad understanding of a wide variety of visual concepts, and gauge how easily a representation can adapt to a new learning task on concepts a (pretrained) model has likely encountered before. We also test datasets that

instead require that the model is able to differentiate between very fine-grained differences in related concepts. Concretely, we test datasets such as Caltech-UCSD Birds 200 (CUB-200) Birds [16], Stanford Cars [17], Flowers [18], Oxford-IIIT Pets [19] and the UC Merced Land Use Dataset [20]. For our evaluation, we freeze the model weights, and train a linear classifier on top of the extracted image representation [21]. On all datasets, we test two few-shot settings: the extreme case where only a single example per class is provided ('1-shot') and the more conservative, but still challenging '10-shot' challenge.

In Fig. 4, we compare the few-shot performance of pre-trained models vs. their few-shot performance after being aligned-finetuned. AligNet finetuning is clearly beneficial on most datasets in both 1-shot and 10-shot evaluations. Even models that have previously been trained on ImageNet data (e.g. DINOv1 ViT-B/16 and supervised ViT-B/16) show improvements on 1-shot ImageNet performance, which indicates that the benefits do not merely come from being exposed to ImageNet data, but also from the label information we distilled into AligNet.

Tip-Adapter. Muttenthaler et al. [22] have demonstrated that linearly transforming the pretrained representations of state-of-the-art image/text models into a human-aligned concept space can improve upon methods that were specifically designed for improving the performance of pretrained representations in few-shot learning tasks. One of these methods is Tip-Adapter [23]. Tip-Adapter is designed to yield effective few-shot classifiers for image/text models by linearly combining the output of two modules — a *zero-shot classifier* and a *key-value cache model*. UD (see Methods) and gLocal [22] have highly similar learning objectives: both linearly transform the representations of pretrained vision foundation models into a (global) human-aligned concept space while preserving the models' nearest neighbor structure. Since we find that UD either performs equally well or better in matching the human similarity judgments and predicting the human RTs for the triplets in the Levels data (see SI. A.2.7) and *soft-aligned* models outperform the UD transformation across the different human object similarity tasks that we evaluated (see SI. A.1 for details), we can be

confident that *soft-aligned* image/text models (i.e., image/text models fine-tuned on AligNet) can improve upon Tip-Adapter. For CIFAR-100, the improvements in zero-shot performance of using gLocal in conjunction with Tip-Adapter compated to Tip-Adapter alone ranged from 6.29% for a CLIP-RN50 up to 18.72% for a CLIP ViT-L/14 (see Appendix D in Muttenthaler et al. [22]). For other datasets, the improvements in zero-shot performance were similarly high. One caveat with Tip-Adapter is that its application is restricted to models that have a text encoder module, i.e., image/text models. Soft-alignment, on the other hand, does not suffer from the same constraint and can be applied to any vision model.

A.2.2. Out-of-distribution

In this section we investigate the how robustly a neural network has learned visual concepts: how well can they recognize objects under different, more challenging settings? This is an important question for many applications, and gives an idea both of the generalization properties, as well as to the practical applicability of models. To investigate robustness, we look at six different out-of-distribution testsets for the popular ImageNet dataset in Fig. 5a. ImageNet ReAL [24] relabels the dataset to fix inaccuracies/oversights in the original data. ImageNet V2 [25] is a new, updated test-set for the original dataset. ImageNet-A [26] consists of adversarial examples, whose aim is to confuse/trick modern systems. ImageNet-C [27] tests how well models can deal with corrupted inputs, e.g. very noisy or blurry images. Finally, ImageNet-R [28] shows more abstract renditions of actual objects, for example scetches, sculptures or drawings.

As we see in the results, AligNet finetuning has a positive impact on the models under investigation, with most datasets showing slight improvements. On average over all models and across all datasets, accuracy went up by 2.6 percentage points, which on Imagenet-like datasets is considered a large performance boost [24]. As can be expected, the models that were pre-trained on imagenet already showed the smallest improvements. Even on ImageNet ReAL, which can be considered a more reliable measure of model improvement than the original Imagenet, on average the models improved by 1.5%. The clearest advantage of

AligNet can be seen for adversarial examples where AligNet-finetuned models exhibit much better behavior than their baseline counterparts: the average improvement was a jump of 5.6 percentage points. For corruptions and renditions, the situation is mixed: while on average the results still show an improvement of 1.8% on ImageNet C and 1.4% on ImageNet R, the SigLIP models slightly deteriorated. Overall, this fits with our understanding of what AligNet is meant to achieve: it allows models to learn clearer concepts that generalize better, but it is not improve performance in situations where image corruption or artistic renditions are the cause of misclassifications.

In their comprehensive survey on the robustness of computer vision models, Liu et al. [29] compare state-of-the-art training methods for improving the robustness of Vision Transformers ("ViTs"), namely Adversarial Training ("AT"), Masked Autoencoder ("MAE"), and Contrastive Language-Image Pretraining ("CLIP"). These methods have been shown to substantially improve robustness upon vanilla supervised or pure image self-supervised training. Similarly to AT, our method can be employed in addition to the other four methods evaluated in that survey. In our work we align SSL models similar to MAE, such as DINO and DINOv2, and models pretrained using CLIP (we use SigLIP which is a variant of CLIP). We find that soft-alignment performs comparably to AT (e.g., ImageNet-A, ImageNet-R). On average, it is neither significantly worse nor significantly better than AT and substantially improves upon the non-aligned base model representations (see Tab. 6). Since ViT-B/16 is the current defacto standard architecture in computer vision, we use it as the basis for all of our soft-aligned models and compare against the numbers for ViT-B reported in Liu et al. [29] (see Tab. 6). We note that while our method is not primarily targeted toward optimizing OOD robustness (or has an adversarial training component), improved OOD robustness appears to be a useful side-effect that emerges from transforming the model representations into a more human-aligned and interpretable space via our method.

Backbone	(Pre-)Training	IID				Real-world OOD				Synthesized OOD
		IN-Val	IN-21K	IN-V2	IN-Real	ON	IN-A	IN-R	IN-V	IN-C
ViT-B/16	Vanilla	75.7	**91.7**	61.6	80.9	20.8	11.3	32.8	24.2	34.3
	MAE	**83.6**	90.5	73.1	**88.1**	37.4	37.4	49.8	36	49.4
	CLIP	68.4	69.8	61.9	75.1	**45**	**50**	77.7	**39.4**	29.3
	AT	73.4	86.7	60.4	80.6	19.2	8.8	50.7	20.2	36.6
	Vanilla + AligNet	77.6	—	65.4	83.6	—	15.2	40.2	—	72.3
	SigLIP + AligNet	82.8	—	**73.3**	87	—	37	**81.4**	—	**74.8**

Table 6 | Comparison of state-of-the-art (pre-)training methods for improving downstream task performance of computer vision models [cf. 29] and soft-aligned models (i.e., models fine-tuned on AligNet) for various ImageNet-based evaluation datasets, testing a model's performance either on i.i.d. data or its OOD robustness. Boldface indicates the best performance for a dataset.

A.2.3. Distribution shift

To measure whether the global category structure induced by fine-tuning on AligNet helps to alleviate model problems with distribution shifts between the training and the test set, we evaluate our models on the BREEDS benchmarks [30]. These were specifically designed to test generalization under input distribution shifts, by constructing datasets where the samples in the training and test sets are sampled from different subpopulations. For example, the training set for the "dog" class contains some selected dog breeds in the training set (e.g., Dalmatians and Schnautzers), whereas a disjoint set of dog breeds is used for the test set (e.g.. Poodles and German Shepards).

In this section we expand on the results presented in Sec. 2.3 in the main text and evaluate all of our AligNet fine-tuned student models (see Methods for details) on BREEDS. For each model, we train a linear probe on the four BREEDS training sets—"entity13", "entity30", "living17", and "nonliving26"—, and evaluate that probe on the corresponding test sets.

AligNet fine-tuning consistently improves performance for all model types across all of the BREEDs benchmarks. We find that the poorer the baseline performance of a student model is, the more that model benefits from fine-tuning on AligNet. For example, the baseline performance of the randomly initialized ViT-B model (which we refer to as "Scratch") is by far the worst but after AligNet fine-tuning that model performs as well as the pretrained student models for "entity13" and "entity30". For the "living17" and "nonliving26" datasets

its performance is slightly worse (but much less than before fine-tuning) than that of the other models, which may be due to the missing pretraining step. Conversely, SigLIP-So400m—the teacher model—has the strongest base performance and thus benefits the least from finetuning. It is striking, however, that even the teacher model consistently improves on BREEDS although that is the model that we used to generate AligNet. For "entity30" some of the student models even outperform the teacher model. This is suggestive evidence that the human part of the AligNet data plays a larger role for these kind of analyses than the teacher model part.

Another interesting observation is that larger supervised ViT models have worse base performance than smaller supervised ViT models but benefit notably more from AligNet fine-tuning than the smaller models. Before AligNet fine-tuning the order of the models according to their base performance is ViT-S, ViT-B, ViT-L across all four datasets. This order is reversed after AligNet fine-tuning for all four datasets. Thus, similarly to the human alignment results presented in Fig. 3, there seems to exist a small scaling effect where larger models benefit more from AligNet fine-tuning, at least for the models that are not the teacher itself. We show all model results for the BREEDS dataset in Fig. 5b.

A.2.4. Additional Experimental Results

CLIP. In the main paper we have focused on SigLIP [31] as the representative model for image/text contrastive models because it is a very strong model that significantly improved upon the more widely known CLIP [32] model. Here we show results for a CLIP ViT-B student model that we finetuned on AligNet using the same setup and hyperparameters as we did for the SigLIP model. As can be seen in Fig. 4, for few-shot learning the CLIP model generally benefits more from AligNet finetuning than SigLIP, while its final performance remains below that of the SigLIP model. The same pattern, can be seen for ImageNet and out-of-distribution performance (see Fig. 5a), and distribution shifts (see Fig. 5b). This further confirms the benefits of our method, and also justifies the focus on SigLIP in the main paper.

SigLIP2 The opposite pattern emerges when comparing SigLIP with the more recent SigLIP2 [33]: SigLIP2 has even stronger baseline performance than SigLIP and unsurprisingly benefits less from finetuning on AligNet. As can be seen in Fig. 4 and Fig. 5b, it still does benefit in most cases for fewshot-generalization and distribution shift cases. For the out-of-distribution experiments in Fig. 5a, on the other hand, the effect is negligible or even detrimental. Given the strong baseline performance of SigLIP2 it would be fruitful to use the largest SigLIP2 as an even stronger teacher model, which we leave for future work.

A.2.5. Training on AligNet from Scratch

AligNet finetuning provides consistent improvements across many different models, settings, and datasets. This begs the question whether pretraining is actually needed, or if we can train a randomly initialized model solely on AligNet and achieve similar results. To answer this question, we trained a ViT-B/16 architecture from scratch on the AligNet dataset using the KLD triplet objective outlined in Eq. (6). We optimize for 1M steps with the Adam optimizer using a cosine decay learning rate schedule with a peak learning rate of 0.0003, $\beta_1 = 0.9$, $\beta_2 = 0.999$, weight decay of 0.1, $\tau^\dagger = 1000$, and a batch size of 1024. These hyperparameters were determined via grid search.

The results are shown in Figs. 4, 5a, 5b and 10, with the model listed as “Scratch”. They roughly fall into two clusters: The first one concerns the distribution-shift experiments on the Breeds dataset (see Fig. 5b), and odd-one-out accuracy on the THINGS dataset (see Fig. 10a). Here our Scratch model performs almost as well as the other pretraining + finetuning models. This means that these tasks are quite closely related to the benefits that the AligNet fine-tuning provides.

The seconds cluster comprises the few-shot (see Fig. 4) and ImageNet out-of-distribution results (see Fig. 5a). While the Scratch model improves substantially over training, on these tasks its performance clearly falls far short of that of the other pretrained models. For example it achieves approx. 27% 1-shot accuracy on ImageNet compared to the 56%

accuracy of the same-sized SigLIP ViT-B model. Pretraining on other vision tasks is thus still important to augment AligNet training and clearly instills important complementary capabilities in a neural network model.

A.2.6. Perceptual patch similarity evaluations

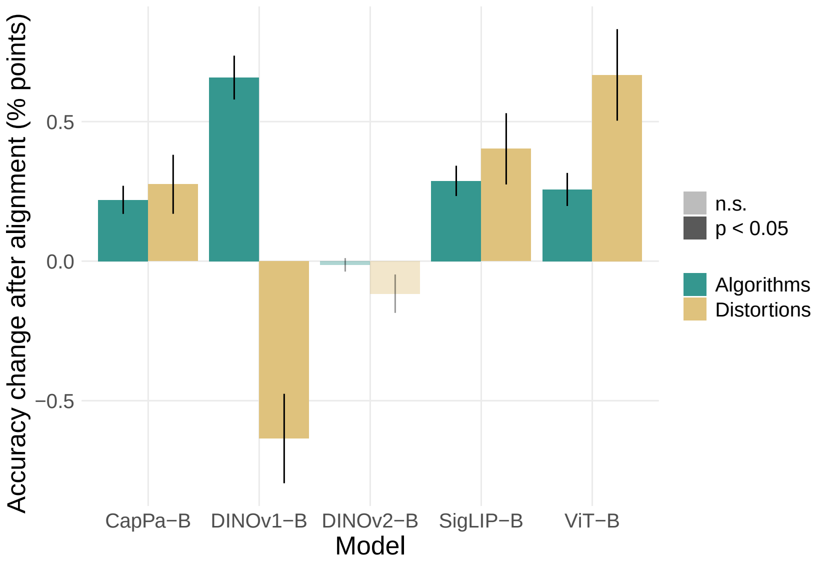

Figure 8 | Performance change on the BAPPS dataset after alignment. All changes are relatively small in absolute magnitude. Most of the AligNet models improve across both subsets of the benchmark, with the exception of the DINOv1 model, which shows significant improvements on Algorithms, but worsens on Distortions, and DINOv2, which changes nonsignificantly. These results suggest that AligNet tuning may slightly improve perceptual judgment performance in most cases. (Colors denote split subsets from the original work; transparent bars are non-signficant changes.)

Model		Distortions		Algorithms			
		cnn	traditional	color	deblur	frameinterp	superres
CapPa-B	base	84.62	59.26	63.62	60.83	54.87	65.33
CapPa-B	AligNet	84.96	59.47	64.22	60.93	54.98	65.40
DINOv1-B	base	85.23	60.28	63.79	60.96	55.08	65.47
DINOv1-B	AligNet	84.87	59.36	65.59	60.97	55.72	65.65
DINOv2-B	base	82.84	61.36	63.79	59.75	54.03	63.46
DINOv2-B	AligNet	82.71	61.25	63.83	59.65	54.03	63.46
SigLIP-B	base	84.34	59.39	62.78	61.01	54.18	65.10
SigLIP-B	AligNet	84.79	59.75	63.69	60.95	54.45	65.13
ViT-B	base	84.92	59.26	63.37	61.05	56.09	65.40
ViT-B	AligNet	85.17	60.34	64.64	61.07	55.83	65.40

Table 7 | Evaluations of models on the BAPP dataset, before and after AligNet tuning.

We also evaluated our AligNet models on the BAPPS Berkeley-Adobe Perceptual Patch Similarity (BAPPS) dataset [34]. This benchmark consists of human similarity judgments, but focuses on a different level of analysis—perceptual similarity of small image patches under

distortion. Accordingly, we follow the original work in analyzing representations aggregated across the early layers of the models (specficially, we used the the first 5 transformer layers, analogous to the 5 convolutional layers used by the original authors). Analyses on this task therefore present a different test of how our alignment procedure affects model capacities—including the effect at earlier layers.

We show the changes between the base and AligNet models in Fig. 8 and the absolute results in Tab. 7. In general, the effects are very small—presumably both because this type of perceptual similarity is relatively different from the semantic structure we focused on, and because our alignment procedure is only explicitly targeted at representations much later in the model. Nevertheless, we generally see significant improvements (via exact binomial tests on the proportion of changed answers where AligNet improves over the base model) for the models across both subsets of the dataset. The exception is the DINO models — DINOv1 gets significantly worse at the Distortions subset, though it still improves significantly on Algorithms, while DINOv2 changes nonsignificantly for both. We find these results to be overall promising—they suggest that AligNet tuning is not dramatically harming other types of perceptual judgment performance, and in fact may slightly improve it for most models.

A.2.7. Ablations

We performed several ablations to examine how the effects of AligNet change if we modify some of its central parameters.

Model Capacity. To investigate if AligNet finetuning affects models of different capacity/size in a similar way, we compared the performance of the ViT-B model (87M parameters) with the performances of a smaller ViT-S (22M parameters) and a larger ViT-L (305M parameters) model respectively. The results in Fig. 4 and Fig. 5a indicate that the positive impact of AligNet fine-tuning increases with model scale. For example, the average 10-shot performance improved by 2.4% points for ViT-S, by 6.3% points for ViT-B and by 8.3% points for ViT-L. We observe similar results for all datasets we considered. Thus, we hypothesize that a larger

model capacity makes it easier for a model to integrate the additional global coarse-grained information provided by AligNet's human-like similarity judgments.

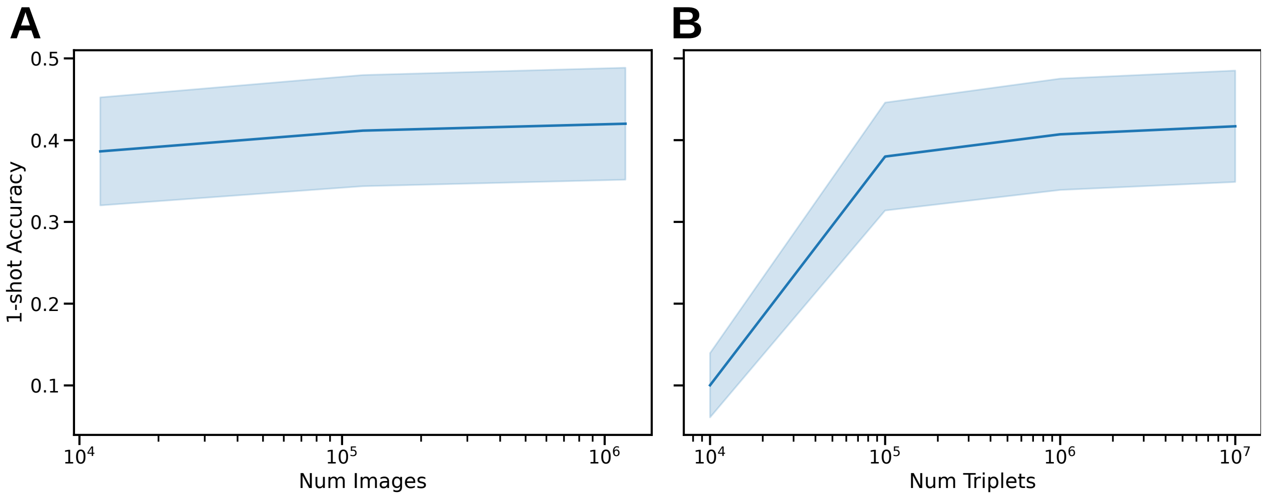

Figure 9 | Accuracy in 1-shot linear probing over a number of common few-shot evaluation datasets when scaling **A** The number of images or **B** the number of triplets. Error bars show the standard error when averaging over datasets.

Increasing the Number of Images or Triplets. We also wanted to see how AligNet scales with the amount of available training data. We individually varied the number of ImageNet images we used to construct our dataset over 3 orders of magnitude, and the number of triplets that we sampled across 4 orders of magnitude. We then looked at the 1-shot results across all the few-shot datasets described in Appendix A.2.1 (Outcomes for 10-shot were not qualitatively different). The results in Fig. 9a show that AligNet performance only increases slightly as the number of available base images increases. However, as we can see from Fig. 9b, the number of generated triplets does influence the outcome, though we hit diminishing returns eventually. We do not expect to be able to increase AligNet performance much further be simply increasing the amount of available data.

Using an Unaligned Teacher Model. An important part of the AligNet pipeline is to align the teacher model to human similarity judgments using a specially trained affine transformation. Here we investigate the effect of this step, by generating a variant of AligNet without this transformation that we call UnAligNet. For this dataset we do not use any human odd-one-out data, so finetuning on UnAligNet is equivalent to distilling the teacher model using our triplet

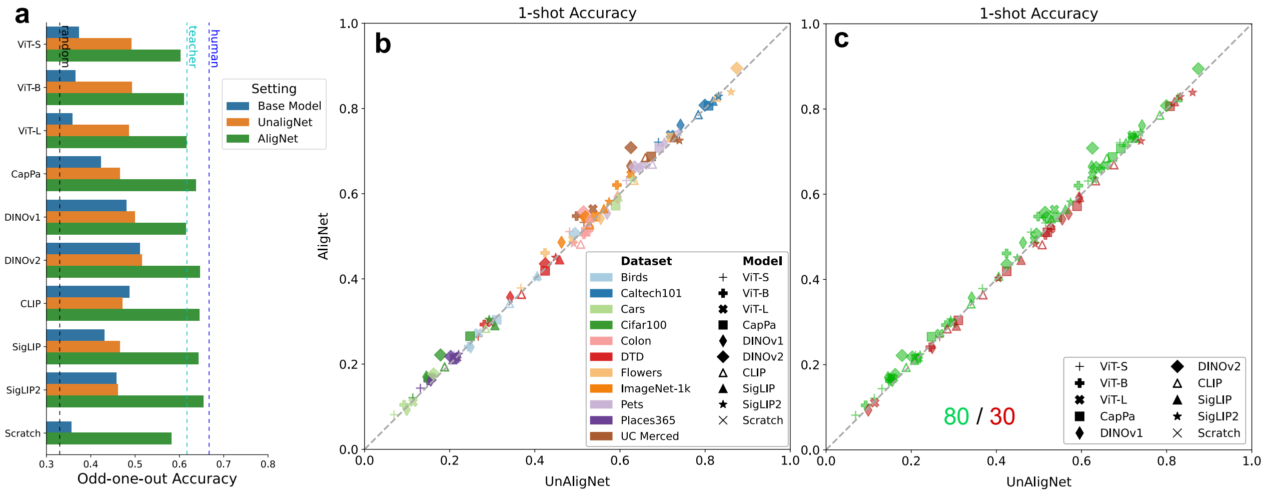

Figure 10 | **a:** Odd-one-out accuracies on the THINGS dataset for different models in different settings. This figure is similar to (an extended version of) the THINGS plot in Fig. 2A. “AligNet” and “Base Model” refer to the pretrained student model, without and without finetuning on AligNet respectively. UnAlignet refers to a student finetuned on a variant of the AligNet dataset with an unaligned teacher. **b:** Comparing 1-shot accuracy on 11 datasets after fine-tuning on UnaligNet (x-axis) with fine-tuning on AligNet (y-axis). Most points lie slightly above the diagonal indicating that alignment of the teacher leads to a small but significant improvement in 1-shot performance of the student. This ablation shows the importance of aligning the teacher model for getting aligned student models. **c:** A variation on **b** which colors each marker as green if it represents an improvement from AligNet finetuning, vs red for a degradation of performance. Note that 80 out of the 110 model/data combinations led to an improvement.

sampling procedure. Fig. 10A compares the odd-one-out accuracy of various student models when fine-tuning on AligNet vs on UnaligNet. As expected, we find that using an unaligned teacher leads to substantially worse alignment of the student models. Though it should be noted that some models (especially the ViTs trained on ImageNet) can already profit even from an unaligned teacher.

It is perhaps unsurprising that an aligned teacher improves the alignment of the student models. A more interesting question is, whether an aligned teacher also improves the students performance on other tasks such as few-shot classification. Fig. 10B thus compares 1-shot performance after training on UnaligNet (x-axis) with performance after training on AligNet (y-axis). The first thing to note here, is that the differences between AligNet and UnaligNet are much smaller than the differences between AligNet and the Base Model (cf. Fig. 4). This shows that the few-shot improvements are in large part due to the teacher model and our distillation method. But importantly, the vast majority of remaining improvements are positive, which means that using an aligned teacher gives a consistent advantage over an unaligned teacher.

We confirm the significance of this trend by fitting a repeated measurements ANOVA to the accuracy on the different datasets, and treating the choice of model and the fine-tuning method (AligNet vs UnaligNet) as conditions. Based on this ANOVA, the null-hypothesis that the fine-tuning do not affect the 1-shot accuracy, can be rejected at the $p < 0.01$ level.

Investigating different Triplet Sampling Schemes. Sampling triplets is an important part of the AligNet pipeline, and the choice of triplets can greatly influence the effect and efficacy of the student finetuning. Here we compare three different sampling strategies:

- **Cluster-border sampling**: This is the strategy used for AligNet. It is unsupervised and uses a k-means clustering of the teacher representations to sample triplets with two nearby images (same cluster) and one far away image (different cluster).
- **Class-border sampling**: This strategy is analogous to cluster-border sampling, but instead of clusters it uses the ImageNet labels to sample two nearby images (same

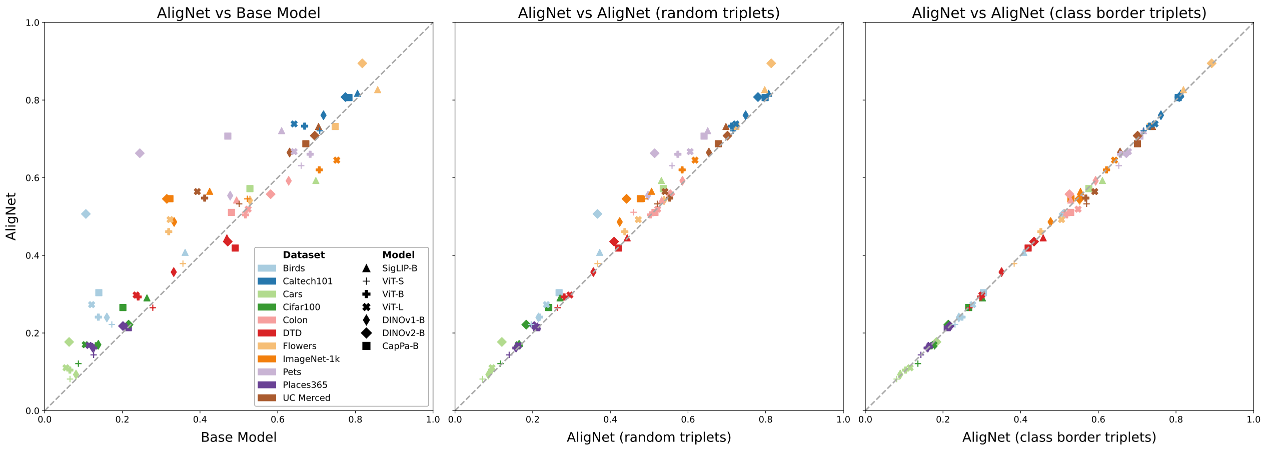

Figure 11 | 1-shot accuracy on various image datasets, comparing performance of an AligNet trained model (y-axis) with three other reference models. **Left:** Again comparing base-model (x-axis) with AligNet (y-axis) for reference. **Middle:** Comparing students fine-tuned using random triplets (x-axis) with AligNet (y-axis). **Right:** Comparing students fine-tuned using class-border triplets (x-axis) with AligNet (y-axis).

class) and one far away image (different class).

- **Random sampling**: As a control we also compare to fully random sampling of triplets, i.e. each image is chosen with equal probability from the full set of all images.

Fig. 11 shows the 1-shot accuracy of student models when comparing different sampling strategies. In all three panels, the y-axis corresponds to the performance of a model after AligNet-fine-tuning. The left panel, again shows how this compares to the performance of the (not fine-tuned) base model (x-axis). The middle panel compares shows how AligNet performance compares to fine-tuning on random-triplets (x-axis). Finally, the right panel shows comparison between AligNet and fine-tuning on triplets sampled from class-borders. Notice that the difference in the middle panel between random triplet sampling is consistently and substantially worse than cluster-based sampling. This clearly highlights the importance of the triplet sampling for down-stream performance. The panel on the right, on the other hand, shows that there is virtually no difference between cluster-border sampling and class-border sampling. So when label information is available, that can effectively be used for sampling triplets, but even if there is not, the clustering based sampling will work just as well.

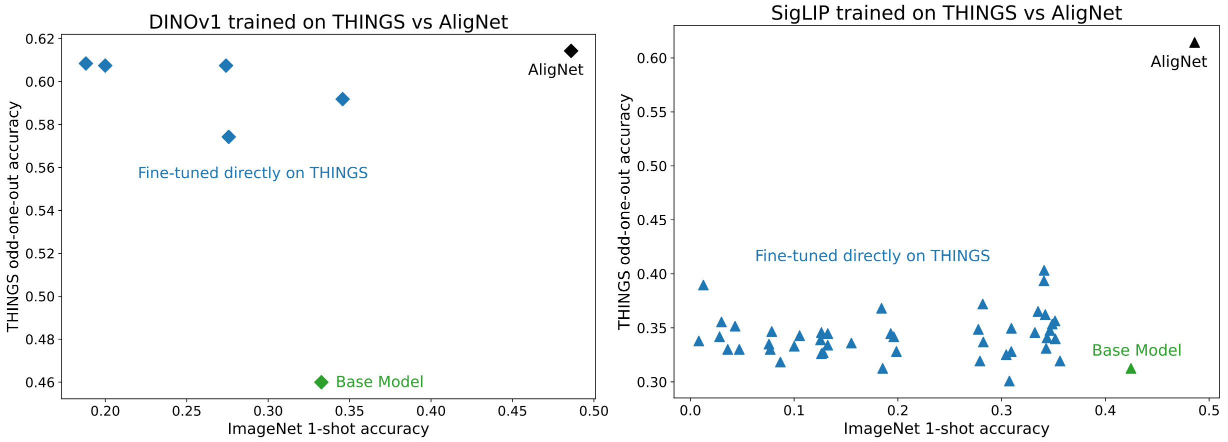

Figure 12 | Performance on two different tasks: ImageNet 1-shot accuracy (x-axis) and odd-one-out accuracy on THINGS (y-axis) for two models (DINOv1 on the left, SigLIP-B on the right). The green markers correspond to the base model, and the black marker to the model fine-tuned on AligNet. The different blue markers in the scatterplot correspond to different hyperparameter settings (learning rate, weight decay, . . .) for fine-tuning directly on THINGS.

Fine-tuning directly on the THINGS data. We have stressed that fine-tuning models to better align them with human similarity judgments depends on having sufficiently large datasets, which do not exist yet. This argument has motivated our multi-stage approach of first linearly aligning a teacher model and then using this as a surrogate model to generate a large corpus of human-like triplet odd-one-out choices. Yet, if we look at Fig. 9, it seems that decreasing the number of images or the number of triplets by up to two orders of magnitude still retains most of the benefit on downstream task performance (in terms of few-shot accuracy). This raises the question whether our pipeline as a whole might be unnecessary, given that the THINGS dataset [35] already contains 4.70 million human similarity judgments collected via online crowd- sourcing for 1854 object images.

Therefore, here we compare soft-alignment with using the THINGS odd-one-out triplets directly for fine-tuning student models. Fig. 12 shows that fine-tuning directly on THINGS is consistently worse compared to training on AligNet in terms of ImageNet 1-shot accuracy (x-axis). Importantly, it appears that in almost all cases the performance of the model actually decreases below that of the base model. It is further interesting to note that even in terms of THINGS odd-one-out accuracy, the AligNet trained models perform better than the any other model. This is somewhat surprising, because it means that there clearly exists an overfitting

problem when training on the THINGS data (even with strong ℓ_2-regularization), which seems to mostly disappear when using AligNet for fine-tuning. One reason for this could be the differences in the sampling procedure (cluster-based vs. random sampling) and the loss function (soft-choices vs. hard-choices) between AligNet and THINGS.

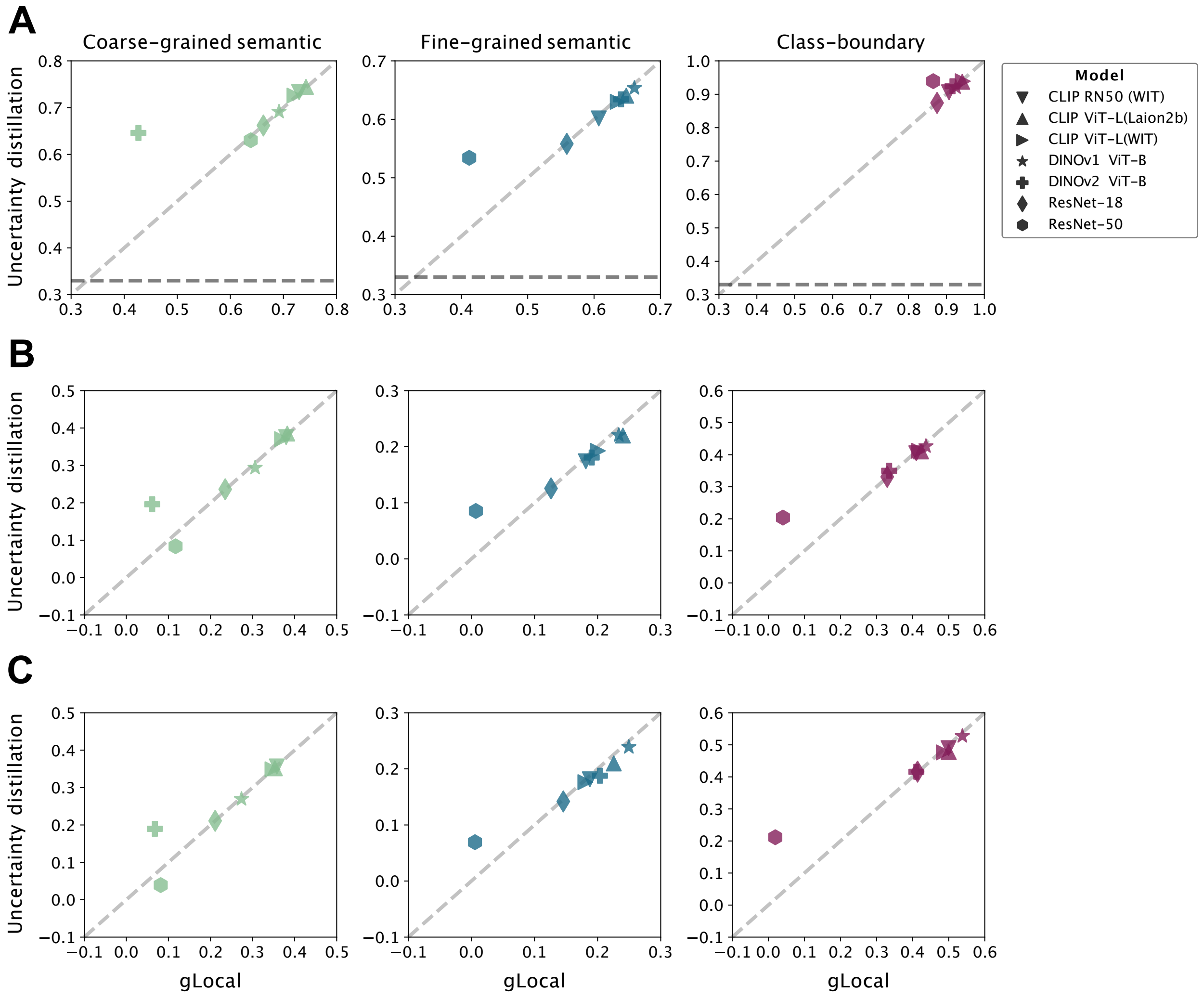

Figure 13 | gLocal vs. UD on the Levels data. Here, we compare gLocal against UD for the different abstraction levels in the Levels dataset. **A**: Fraction of triplets where humans and models selected the same odd-one-out image. **B**: Spearman rank correlations between the human disagreement levels (using the five responses per triplet) and the model uncertainties over the triplets. **C**: Spearman rank correlations between the aggregated human response times (in log-space) and the model triplet uncertainties. Dashed horizontal lines in the panels of the top row indicate random guessing.

Uncertainty Distillation vs. gLocal. Here, we compare the Uncertainty Distillation (UD) transformation that we introduced in the Methods against gLocal [cf. 22]. The learning objectives of the two linear transformations are highly similar and the goal is the same: the

representations of a pretrained vision foundation model are linearly transformed into a global coarse-grained human object similarity space using the THINGS dataset [4] while the nearest neighbor structure of the model's representation space is preserved. However, the difference between gLocal and UD is that UD directly injects the human uncertainty estimates for the triplet odd-one-out choices via approximate Bayesian inference [cf. 36] into a model's representation space and, thus, uses soft rather than hard triplet choices (see Methods for details). We use the Levels datasets (see Methods) for evaluating the UD and gLocal transformations, comparing the same set of models that were evaluated in Muttenthaler et al. [22]. The gLocal transformations for those models are publicly available via the Python library `thingsvision` [37] which we used for this analysis. In Fig. 13 we see that the UD transformation is either equally accurate or better in aligning the model's representations with the human similarity judgments, and predicting their disagreement levels and aggregated RTs respectively in the different abstraction level compared to gLocal. As before, human RTs are measured in log-space and model uncertainties are measured as discrete Shannon entropy over the triplet odd-one-out choices. Note that UD is computationally more efficient than gLocal because it does not require an extra set of images—in addition to the THINGS data—to evaluate the preservation of a representation's nearest neighbor structure which the multi-objective of gLocal necessitates [cf. 22].

A.3. Qualitative Analysis of Representations

A.3.1. Principal Components Analysis of Representations

In Extended Data Fig. 1a we show PCA projections of the space of image representations from four different models before and after fine-tuning on AligNet. It shows that the large-scale structure of the representational space becomes more structured and interpretable. Notice for example that animals (blue) and food (green) are clearly separated from artifacts such as furniture (red), musical instruments (purple) and clothing items (orange) which are much more clustered together. The same structuring effect — though to a lesser degree — can be seen for principal components three and four (see Fig. 14).

This increased structuring within the first few principal components is also reflected in the amount of variance explained by them. The third row of Extended Data Fig. 1a clearly shows a notable increase of the proportion of the total variance of the representations that can be explained with only the first 5-15 principal components. It is also striking how different the global structure of the four models is before fine-tuning on AligNet, and how similar it becomes afterwards.

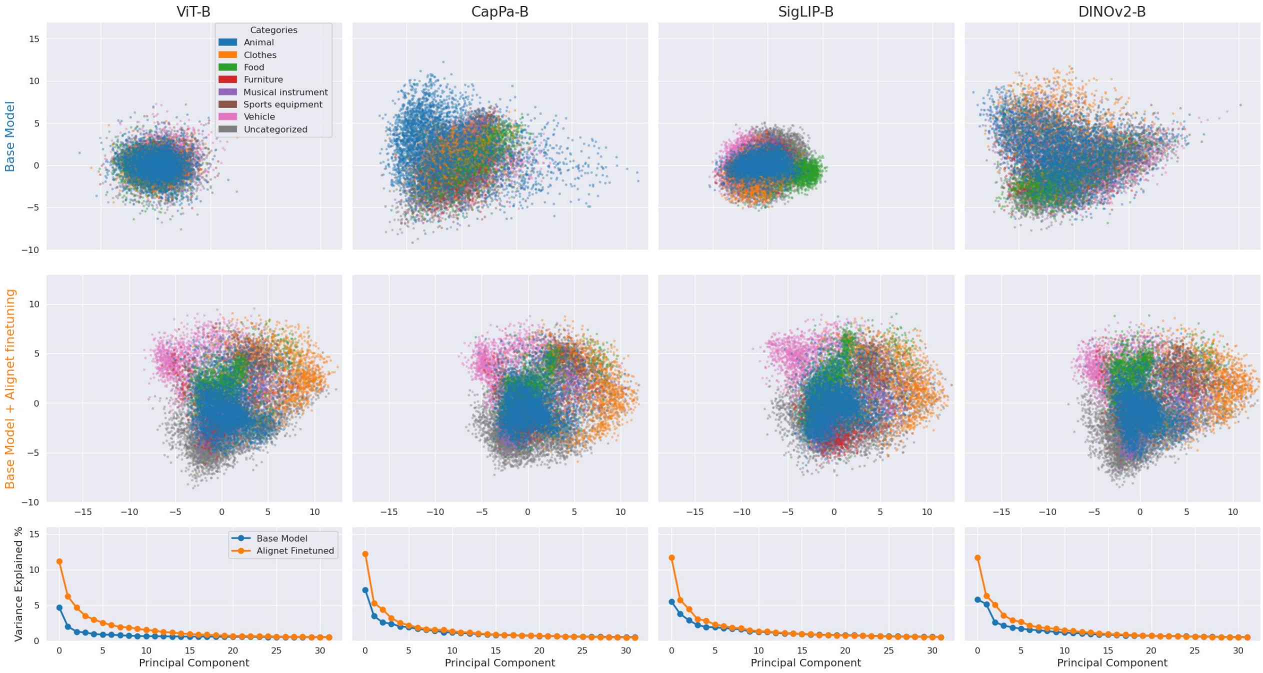

Figure 14 | Projection of the representations of 85k ImageNet images from four different models (columns) onto their first two principal components both before (first row) and after fine-tuning on AligNet (second row). Similar to Extended Data Fig. 1a but for components 3 & 4.

In Extended Data Fig. 1b we further show projections of the representations using TriMap [38], which is a non-linear dimensionality reduction technique designed to better preserve both the global and the local structure of the data. While the coarse structure is similar to that of the first two PCs (Extended Data Fig. 1a), the fine-grained structure reveals some interesting details. Note for example that in the TriMap projection the ViT-B exhibits a lot of fine-grained structure in the form of tiny clusters.

A.3.2. Detailed results on alignment with the semantic hierarchy

In Extended Data Fig. 2a we show the distribution of changes in relative distance between the representations of pairs of ImageNet images for four models. We measure this distance change relative to other changes (by z-scoring), because relative distances are more meaningful than absolute ones (e.g., scaling all representations by a factor of two would change absolute distances, but not relative ones), and absolute scales of all representations tend to increase during training.

In general, images from classes that come from the same superordinate category (such as two different species of bird) tend to end up relatively closer together, while those from different superordinate categories tend to end up farther apart. Images that come from the same basic- or subordinate-level category move even closer together. The exact distribution of changes depends on the prior representation structure of the models; the effect is stronger for models that had lower-quality initial representations.

In Fig. 15 we show a more detailed visualization, showing a matrix of average changes in the relative distances between a subset of the *basic-level* categories, grouped into several higher-level semantic categories (animals, clothes, food, furniture, musical instruments, sports equipment, and vehicles), which are plotted as blocks on the diagonals. The block structure of the changes is clear, with overall increases in similarity within these broad categories, and decreases in similarity between them.

Taken together, these results show qualitatively that our alignment process is working as intended—it is reorganizing the representation space of the model in accordance with the structure of human semantic knowledge.

Verifying the statistical significance of the changes. To quantify the statistical significance of the representational changes, we fit mixed-effects linear regressions accounting for the non-independence of the representational changes across different clusters. We use the same-subordinate condition as the reference level, as this offers the most stringent

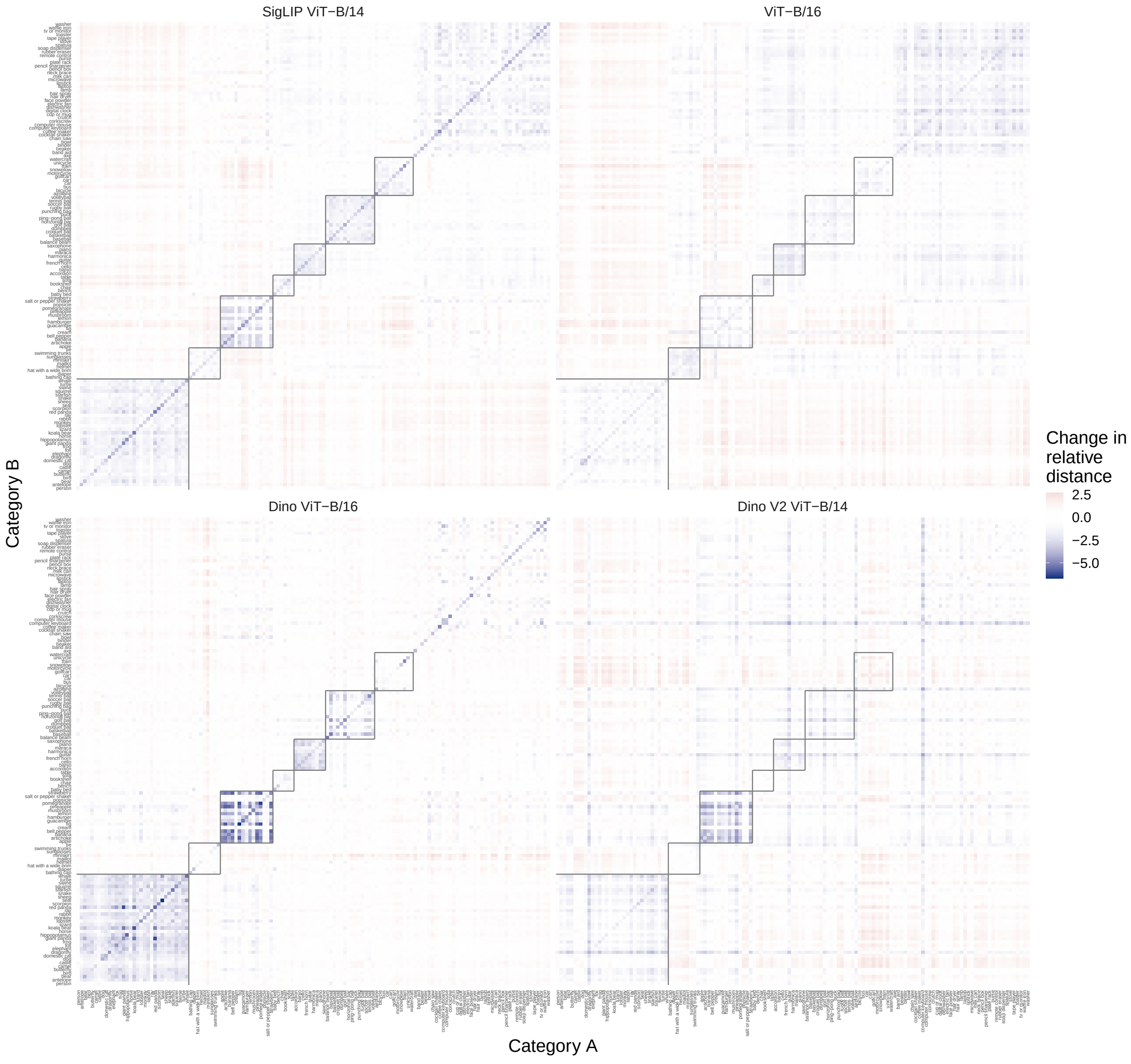

Figure 15 | Changes in relative distances between stimuli are structured by higher-level semantics. After alignment, representations end up relatively more similar to one another within broad categories (diagonal blocks)—particularly human-salient ones like "animals" or "food"—and less similar between these categories.

comparison for the same-basic condition (i.e. the effect size of the difference between the two conditions is visibly the smallest). All effects were generally highly significant, full results are presented in Tab. 8.

	Estimate	Std. Error	df	t value	Pr(>|t|)	
(Intercept)	-2.25018	0.01612	2344.28109	-139.562	< 2e-16	***
relationshipSame basic category	0.25678	0.04675	2344.28108	5.493	4.39e-08	***
relationshipSame superordinate category	1.62585	0.03372	619.98263	48.220	< 2e-16	***
relationshipDifferent superordinate categories	2.72148	0.03229	520.51180	84.275	< 2e-16	***

(a) SigLIP-B

	Estimate	Std. Error	df	t value	Pr(>|t|)	
(Intercept)	-1.73190	0.01931	2280.89874	-89.667	< 2e-16	***
relationshipSame basic category	0.22030	0.05600	2280.89872	3.934	8.62e-05	***
relationshipSame superordinate category	0.98145	0.04498	963.74930	21.818	< 2e-16	***
relationshipDifferent superordinate categories	2.13518	0.04384	870.21854	48.701	< 2e-16	***

Signif. codes: 0 ‘***’ 0.001 ‘**’ 0.01 ‘*’ 0.05 ‘.’ 0.1 ‘ ’ 1

(b) ViT-B

	Estimate	Std. Error	df	t value	Pr(>|t|)	
(Intercept)	-2.18517	0.02733	1127.92111	-79.95	< 2e-16	***
relationshipSame basic category	0.29318	0.07925	1127.92113	3.70	0.000226	***
relationshipSame superordinate category	1.75272	0.07325	819.54322	23.93	< 2e-16	***
relationshipDifferent superordinate categories	2.59777	0.07268	793.50251	35.74	< 2e-16	***

(c) Dino ViT-B

	Estimate	Std. Error	df	t value	Pr(>|t|)	
(Intercept)	-1.11179	0.03202	1312.37727	-34.719	< 2e-16	***
relationshipSame basic category	0.19989	0.09285	1312.37733	2.153	0.0315	*
relationshipSame superordinate category	0.63283	0.08885	1098.25627	7.122	1.92e-12	***
relationshipDifferent superordinate categories	1.40199	0.08847	1079.24964	15.847	< 2e-16	***

(d) Dino V2 ViT-B

Table 8 | Statistical significance of the representation reorganization for the AligNet models, via mixed-effects linear regressions. (Note that same-subordinate category is the reference level; thus, the statistics on the intercept denote the magnitude and significance of the changes in relative distances for that category, and the other coefficients and statistics are relative to that change.

Reorganization across living and nonliving categories Given that the living vs. non-living distinction is extremely salient to humans, and likely plays a large role in the odd-one-out-judgments, we also computed similar analyses to the above focusing specifically on this distinction. Specifically, we evaluated how representation distances change within- and between the living and nonliving categories. We show the results in Extended Data Fig. 2b—the changes are qualitatively as expected. These differences are also all statistically significant for all models (all ts > 2.7, all ps < 0.01) in mixed-effects regressions.

Alignment across layers Although our alignment procedure focuses on aligning the final representations of the model, because we backpropagate to optimize the weights throughout

the network, the representations will still change at earlier layers. However, it is unclear what form this reorganization may take. We therefore investigated whether there is similar reorganization according to the semantic hierarchy at earlier layers. We therefore performed similar representation-change analyses to the above across the representation layers of the model (using the mean representation across spatial positions). We show the results in Extended Data Fig. 2d—generally, we see a growing degree of reorganization across the layers of the model.

Comparing representation changes to UnaligNet Finally, we compare the representation changes in the UnaligNet ablation (see above) for ViT-B. We show the results in Extended Data Fig. 2c. This reorganization is significantly different from that observed in the AligNet version (Tab. 9). Surprisingly, the UnaligNet procedure results in *greater* divergence amongst images within the same basic- or subordinate-level category, compared to the original representations. To gain some qualitative insight into this effect, we compared pairs of images within the same subordinate category on which AligNet and UnaligNet maximally disagree (Extended Data Fig. 2c). The results suggest that perhaps UnaligNet representations are focusing more on superficial features that distinguish images, such as color or style. We validate these results with a human experiment in the next section.

	Estimate	Std. Error	df	t value	Pr(>|t|)	
(Intercept)	1.776e+00	2.470e-02	1.948e+03	71.89	< 2e-16	***
versionAligNet	-3.328e+00	2.013e-02	1.906e+04	-165.38	< 2e-16	***
relationshipSame basic	-3.288e-01	7.162e-02	1.948e+03	-4.59	4.71e-06	***
relationshipSame superordinate	-2.606e+00	6.175e-02	1.076e+03	-42.20	< 2e-16	***
relationshipDifferent superordinate	-1.877e+00	6.086e-02	1.015e+03	-30.84	< 2e-16	***
versionAligNet:relationshipSame basic	4.447e-01	5.836e-02	1.906e+04	7.62	2.65e-14	***
versionAligNet:relationshipSame superordinate	3.232e+00	2.573e-02	1.906e+04	125.60	< 2e-16	***
versionAligNet:relationshipDifferent superordinate	3.846e+00	2.143e-02	1.906e+04	179.48	< 2e-16	***

Table 9 | Statistical significance of the differences in representation changes between UnaligNet and AligNet (interaction of changes by training version). All interactions are highly significant. (Note that the relationship is dummy coded, so the simple effect of versionAligNet shows the difference in the reorganization of the same-subordinate category between the model versions.)

A.3.3. Human validation of the representational differences between AligNet and UnaligNet

In order to further ascertain the origin of the differences between the AligNet and UnaligNet tuned versions of ViT-B, we compared representations within each ImageNet category to find

the largest disagreements between the two models. Specifically, within each category we identified the pair of images for which the models' representations most strongly disagreed in both directions: the pair that the AligNet model represented much more similarly than UnaligNet, and the pair that UnaligNet represented much more similarly than AligNet (Note that in this context, since both models start from the same base model, it is equivalent to compare similarities directly or to compare the change in similarity from the base model.) We took the top 100 disagreements (in magnitude) in both directions across categories, for a total of 200 image pairs — half of which AligNet represents much more similarly than UnaligNet, and half of which UnaligNet represents much more similarly than AligNet. We find that UnaligNet tends to represent images as more similar based on superficial characteristics like texture; e.g., one of the image pairs where UnaligNet and AligNet disagree the most is a close-up image of broccoli and an aerial view of a forest canopy, which UnaligNet rates as highly similar, presumably due to their similar color and texture. By contrast, AligNet rates images images with highly different styles, but the same semantic content, as more similar; e.g. a photo of a red panda and painting of red pandas, or a photo and a 3D rendering of a military vehicle.

We then ran an online human experiment via Prolific ($N = 49$ participants) testing which images participants rated (on a 1-5 Likert scale) as semantically more similar in content, when explicitly instructed to ignore superficial visual features. We present the results in Extended Data Fig. 3a. We find strong alignment between the participants' judgments and the AligNet model — the participants consistently gave higher similarity ratings to the images that AligNet represented as more similar. This effect was highly significant (paired t-test: $t(48) = 20.260, p < 0.001$); in fact, *every* participant gave numerically higher similarity scores to the pairs AligNet rated as more similar. Most importantly, the correlation between model- and human-judged similarity is strongly positive for the aligned model (Spearman =0.641, p<.001) and negative for the unaligned model (Spearman =–0.122, p = 0.087).

These strong results support our claim that AligNet is uniquely changing the model's

representation structure—even within low-level categories—to align more closely with human semantic knowledge.

Supplementary References

[84] Joshua C Peterson, Joshua T Abbott, and Thomas L Griffiths. Evaluating (and improving) the correspondence between deep neural networks and human representations. *Cognitive Science*, 42(8):2648–2669, 2018.

[85] Radoslaw M. Cichy, Nikolaus Kriegeskorte, Kamila M. Jozwik, Jasper J.F. van den Bosch, and Ian Charest. The spatiotemporal neural dynamics underlying perceived similarity for real-world objects. *NeuroImage*, 194:12–24, 2019. ISSN 1053-8119. doi: https://doi.org/10.1016/j.neuroimage.2019.03.031.

[86] Marcie L. King, Iris I.A. Groen, Adam Steel, Dwight J. Kravitz, and Chris I. Baker. Similarity judgments and cortical visual responses reflect different properties of object and scene categories in naturalistic images. *NeuroImage*, 197:368–382, 2019. ISSN 1053-8119. doi: https://doi.org/10.1016/j.neuroimage.2019.04.079.

[87] Martin N. Hebart, Charles Y. Zheng, Francisco Pereira, and Chris I. Baker. Revealing the multidimensional mental representations of natural objects underlying human similarity judgements. *Nature Human Behaviour*, 4(11):1173–1185, 2020. doi: 10.1038/s41562-020-00951-3.

[88] Joshua C. Peterson, Joshua T. Abbott, and Thomas L. Griffiths. Adapting deep network features to capture psychological representations. In Anna Papafragou, Daniel Grodner, Daniel Mirman, and John C. Trueswell, editors, *Proceedings of the 38th Annual Meeting of the Cognitive Science Society, Recogbizing and Representing Events, CogSci 2016, Philadelphia, PA, USA*. cognitivesciencesociety.org, 2016.

[89] Martin N Hebart, Adam H Dickter, Alexis Kidder, Wan Y Kwok, Anna Corriveau, Caitlin Van Wicklin, and Chris I Baker. Things: A database of 1,854 object concepts and more than 26,000 naturalistic object images. *PloS one*, 14(10):e0223792, 2019.

[90] Johannes Mehrer, Courtney J. Spoerer, Emer C. Jones, Nikolaus Kriegeskorte, and Tim C. Kietzmann. An ecologically motivated image dataset for deep learning yields better models of human vision. *Proceedings of the National Academy of Sciences*, 118 (8):e2011417118, 2021. doi: 10.1073/pnas.2011417118.

[91] Lukas Muttenthaler, Jonas Dippel, Lorenz Linhardt, Robert A. Vandermeulen, and Simon Kornblith. Human alignment of neural network representations. In *The Eleventh International Conference on Learning Representations*, 2023.

[92] Talia Konkle, Colin Conwell, Jacob S Prince, and George A Alvarez. What can 5.17 billion regression fits tell us about the representational format of the high-level human visual system? *Journal of Vision*, 22(14):4422–4422, 2022.

[93] Gemini Team, Rohan Anil, Sebastian Borgeaud, Yonghui Wu, Jean-Baptiste Alayrac, Jiahui Yu, Radu Soricut, Johan Schalkwyk, Andrew M Dai, and Anja Hauth. Gemini: a family of highly capable multimodal models. *arXiv preprint arXiv:2312.11805*, 2023.

[94] Gemini Team, Gheorghe Comanici, Eric Bieber, Mike Schaekermann, Ice Pasupat, Noveen Sachdeva, Inderjit Dhillon, Marcel Blistein, Ori Ram, Dan Zhang, Evan Rosen, et al. Gemini 2.5: Pushing the frontier with advanced reasoning, multimodality, long context, and next generation agentic capabilities. *arXiv preprint arXiv:2507.06261*, 2025.

[95] Yaqing Wang, Quanming Yao, James T. Kwok, and Lionel M. Ni. Generalizing from a few examples: A survey on few-shot learning. *ACM Comput. Surv.*, 53(3), June 2020. ISSN 0360-0300. doi: 10.1145/3386252.

[96] Olga Russakovsky, Jia Deng, Hao Su, Jonathan Krause, Sanjeev Satheesh, Sean Ma, Zhiheng Huang, Andrej Karpathy, Aditya Khosla, Michael Bernstein, et al. Imagenet large scale visual recognition challenge. *International journal of computer vision*, 115: 211–252, 2015.

[97] Bolei Zhou, Agata Lapedriza, Aditya Khosla, Aude Oliva, and Antonio Torralba. Places: A 10 million image database for scene recognition. *IEEE Transactions on Pattern Analysis and Machine Intelligence*, 40(6):1452–1464, 2018. doi: 10.1109/TPAMI.2017.2723009.

[98] Alex Krizhevsky and Geoffrey Hinton. Learning multiple layers of features from tiny images. Technical Report 0, University of Toronto, Toronto, Ontario, 2009.

[99] Catherine Wah, Steve Branson, Peter Welinder, Pietro Perona, and Serge Belongie. The caltech-ucsd birds-200-2011 dataset, 2011.

[100] Jonathan Krause, Jia Deng, Michael Stark, and Li Fei-Fei. Collecting a large-scale dataset of fine-grained cars. In *Second Workshop on Fine-Grained Visual Categorization*, 2013.

[101] Maria-Elena Nilsback and Andrew Zisserman. Automated flower classification over a large number of classes. In *Computer Vision, Graphics & Image Processing, 2008. ICVGIP'08. Sixth Indian Conference on*, pages 722–729. IEEE, 2008.

[102] Omkar M Parkhi, Andrea Vedaldi, Andrew Zisserman, and CV Jawahar. Cats and dogs. In *IEEE Conference on Computer Vision and Pattern Recognition (CVPR)*, pages 3498–3505. IEEE, 2012.

[103] Yi Yang and Shawn Newsam. Bag-of-visual-words and spatial extensions for land-use classification. In *Proceedings of the 18th SIGSPATIAL International Conference on Advances in Geographic Information Systems*, GIS '10, page 270–279, New York, NY, USA, 2010. Association for Computing Machinery. ISBN 9781450304283. doi: 10.1145/1869790.1869829.

[104] Guillaume Alain and Yoshua Bengio. Understanding intermediate layers using linear classifier probes. In *5th International Conference on Learning Representations*. OpenReview.net, 2017.

[105] Lukas Muttenthaler, Lorenz Linhardt, Jonas Dippel, Robert A Vandermeulen, Katherine Hermann, Andrew Lampinen, and Simon Kornblith. Improving neural network representations using human similarity judgments. In A. Oh, T. Naumann, A. Globerson, K. Saenko, M. Hardt, and S. Levine, editors, *Advances in Neural Information Processing Systems*, volume 36, pages 50978–51007. Curran Associates, Inc., 2023.

[106] Renrui Zhang, Wei Zhang, Rongyao Fang, Peng Gao, Kunchang Li, Jifeng Dai, Yu Qiao, and Hongsheng Li. Tip-adapter: Training-free adaption of clip for few-shot classification. In Shai Avidan, Gabriel Brostow, Moustapha Cissé, Giovanni Maria Farinella, and Tal Hassner, editors, *Computer Vision – ECCV 2022*, pages 493–510, Cham, 2022. Springer Nature Switzerland. ISBN 978-3-031-19833-5.

[107] Lucas Beyer, Olivier J Hénaff, Alexander Kolesnikov, Xiaohua Zhai, and Aäron van den Oord. Are we done with imagenet? *arXiv preprint arXiv:2006.07159*, 2020.

[108] Benjamin Recht, Rebecca Roelofs, Ludwig Schmidt, and Vaishaal Shankar. Do ImageNet classifiers generalize to ImageNet? In Kamalika Chaudhuri and Ruslan Salakhutdinov, editors, *Proceedings of the 36th International Conference on Machine Learning*, volume 97 of *Proceedings of Machine Learning Research*, pages 5389–5400. PMLR, 09–15 Jun 2019.

[109] Dan Hendrycks, Kevin Zhao, Steven Basart, Jacob Steinhardt, and Dawn Song. Natural adversarial examples. In *Proceedings of the IEEE/CVF Conference on Computer Vision and Pattern Recognition (CVPR)*, pages 15262–15271, 2021.

[110] Dan Hendrycks and Thomas Dietterich. Benchmarking neural network robustness to common corruptions and perturbations. In *International Conference on Learning Representations*, 2019.

[111] Dan Hendrycks, Steven Basart, Norman Mu, Saurav Kadavath, Frank Wang, Evan Dorundo, Rahul Desai, Tyler Zhu, Samyak Parajuli, Mike Guo, Dawn Song, Jacob Steinhardt, and Justin Gilmer. The many faces of robustness: A critical analysis

of out-of-distribution generalization. In *Proceedings of the IEEE/CVF International Conference on Computer Vision (ICCV)*, pages 8340–8349, 2021.

[112] Chang Liu, Yinpeng Dong, Wenzhao Xiang, Xiao Yang, Hang Su, Jun Zhu, Yuefeng Chen, Yuan He, Hui Xue, and Shibao Zheng. A comprehensive study on robustness of image classification models: Benchmarking and rethinking. *International Journal of Computer Vision*, pages 1–23, 2024.

[113] Shibani Santurkar, Dimitris Tsipras, and Aleksander Madry. Breeds: Benchmarks for subpopulation shift. *arXiv preprint arXiv:2008.04859*, 2020.

[114] Xiaohua Zhai, Basil Mustafa, Alexander Kolesnikov, and Lucas Beyer. Sigmoid loss for language image pre-training. *Proceedings of the IEEE/CVF International Conference on Computer Vision (ICCV)*, 2023.

[115] Alec Radford, Jong Wook Kim, Chris Hallacy, Aditya Ramesh, Gabriel Goh, Sandhini Agarwal, Girish Sastry, Amanda Askell, Pamela Mishkin, Jack Clark, Gretchen Krueger, and Ilya Sutskever. Learning transferable visual models from natural language supervision. In Marina Meila and Tong Zhang, editors, *Proceedings of the 38th International Conference on Machine Learning*, volume 139 of *Proceedings of Machine Learning Research*, pages 8748–8763. PMLR, 2021.

[116] Michael Tschannen, Alexey Gritsenko, Xiao Wang, Muhammad Ferjad Naeem, Ibrahim Alabdulmohsin, Nikhil Parthasarathy, Talfan Evans, Lucas Beyer, Ye Xia, Basil Mustafa, Olivier Hénaff, Jeremiah Harmsen, Andreas Steiner, and Xiaohua Zhai. SigLIP 2: Multilingual vision-language encoders with improved semantic understanding, localization, and dense features. *arXiv [cs.CV]*, 20 February 2025.

[117] Richard Zhang, Phillip Isola, Alexei A. Efros, Eli Shechtman, and Oliver Wang. The unreasonable effectiveness of deep features as a perceptual metric. In *Proceedings of the IEEE Conference on Computer Vision and Pattern Recognition (CVPR)*, 2018.

[118] Martin N Hebart, Oliver Contier, Lina Teichmann, Adam H Rockter, Charles Y Zheng, Alexis Kidder, Anna Corriveau, Maryam Vaziri-Pashkam, and Chris I Baker. Things-data, a multimodal collection of large-scale datasets for investigating object representations in human brain and behavior. *eLife*, 12:e82580, 2023. ISSN 2050-084X. doi: 10.7554/eLife.82580.

[119] Lukas Muttenthaler, Charles Y Zheng, Patrick McClure, Robert A Vandermeulen, Martin N Hebart, and Francisco Pereira. VICE: Variational Interpretable Concept Embeddings. *Advances in Neural Information Processing Systems*, 35:33661–33675, 2022.

[120] Lukas Muttenthaler and Martin N. Hebart. Thingsvision: A python toolbox for streamlining the extraction of activations from deep neural networks. *Frontiers in Neuroinformatics*, 15:45, 2021. ISSN 1662-5196. doi: 10.3389/fninf.2021.679838.

[121] Ehsan Amid and Manfred K. Warmuth. Trimap: Large-scale dimensionality reduction using triplets, 2020.
