# Supplementary material for: Aligning machine and human visual representations across abstraction levels
Source: Nature. 2025 Nov 12;647(8089):349–55. doi: 10.1038/s41586-025-09631-6 (PMC12611773; doi:10.1038/s41586-025-09631-6)
Supplement: Supplementary file 2 — Reporting Summary [file 41586_2025_9631_MOESM2_ESM.pdf]

nature portfolio

Corresponding author(s): Lukas Muttenthaler, Klaus Robert-Muller, Andrew Lampinen

Last updated by author(s): Feb 12, 2025

Reporting Summary

Nature Portfolio wishes to improve the reproducibility of the work that we publish. This form provides structure for consistency and transparency in reporting. For further information on Nature Portfolio policies, see our Editorial Policies and the Editorial Policy Checklist.

Statistics

For all statistical analyses, confirm that the following items are present in the figure legend, table legend, main text, or Methods section.

n/a	Confirmed	
☐	☒	The exact sample size (*n*) for each experimental group/condition, given as a discrete number and unit of measurement
☐	☒	A statement on whether measurements were taken from distinct samples or whether the same sample was measured repeatedly
☐	☒	The statistical test(s) used AND whether they are one- or two-sided<br>*Only common tests should be described solely by name; describe more complex techniques in the Methods section.*
☐	☒	A description of all covariates tested
☐	☒	A description of any assumptions or corrections, such as tests of normality and adjustment for multiple comparisons
☐	☒	A full description of the statistical parameters including central tendency (e.g. means) or other basic estimates (e.g. regression coefficient) AND variation (e.g. standard deviation) or associated estimates of uncertainty (e.g. confidence intervals)
☐	☒	For null hypothesis testing, the test statistic (e.g. F, t, r) with confidence intervals, effect sizes, degrees of freedom and P value noted<br>*Give P values as exact values whenever suitable.*
☒	☐	For Bayesian analysis, information on the choice of priors and Markov chain Monte Carlo settings
☒	☐	For hierarchical and complex designs, identification of the appropriate level for tests and full reporting of outcomes
☐	☒	Estimates of effect sizes (e.g. Cohen's *d*, Pearson's *r*), indicating how they were calculated

Our web collection on statistics for biologists contains articles on many of the points above.

Software and code

Policy information about availability of computer code

Data collection	Data collection used a web-browser based presentation of stimuli built on jspysych; the behavioral experiment code is documented in our github repository for the LEVELS dataset.
Data analysis	We used python libraries including numpy, scikit learn, and R libraries (dplyr, tidyr, ggplot) in the analysis of these data.

For manuscripts utilizing custom algorithms or software that are central to the research but not yet described in published literature, software must be made available to editors and reviewers. We strongly encourage code deposition in a community repository (e.g. GitHub). See the Nature Portfolio guidelines for submitting code & software for further information.

Data

Policy information about availability of data

All manuscripts must include a data availability statement. This statement should provide the following information, where applicable:

- Accession codes, unique identifiers, or web links for publicly available datasets
- A description of any restrictions on data availability
- For clinical datasets or third party data, please ensure that the statement adheres to our policy

The synthetically created AligNet data will be made publicly available upon publication. We already made the Levels data available on GIN prior to publication: https://doi.org/10.12751/g-node.

hg4tdz. Both the analysis and the experiment code for Levels are hosted on GitHub and archived on the Zenodo platform (see https://zenodo.org/doi/10.5281/zenodo.13749101).

Research involving human participants, their data, or biological material

Policy information about studies with human participants or human data. See also policy information about sex, gender (identity/presentation), and sexual orientation and race, ethnicity and racism.

Reporting on sex and gender	Of the 508 participants in the LEVELS data collection, there were 209 who identified as female, and 289 who identified as male, 3 diverse, and 7 missing demographic information. We did not incorporate gender into our analyses as there is little a priori reason to expect it would affect these basic semantic cognition effects.
Reporting on race, ethnicity, or other socially relevant groupings	Per the IRB, we did not collect demographic information on race, ethnicity, etc. as it did not seem relevant to the experiments performed.
Population characteristics	The eligibility criteria were that participants had to be between 18 and 50 years old, fluent in English, have a normal or corrected-to-normal vision, no colorblindness, and have a minimum approval rating of 95% on Prolific. These criteria were applied through Prolific recruitment filters.
Recruitment	We recruited participants online via Prolific Academic (https://www.prolific.ac).
Ethics oversight	Participants provided informed consent before starting the experiment. The experiment was approved by the internal review board (IRB) of the Max Planck Institute for Human Development

Note that full information on the approval of the study protocol must also be provided in the manuscript.

Field-specific reporting

Please select the one below that is the best fit for your research. If you are not sure, read the appropriate sections before making your selection.

☐ Life sciences ☒ Behavioural & social sciences ☐ Ecological, evolutionary & environmental sciences

For a reference copy of the document with all sections, see nature.com/documents/nr-reporting-summary-flat.pdf

Behavioural & social sciences study design

All studies must disclose on these points even when the disclosure is negative.

Study description	We collected a new multi-level similarity judgment dataset from N = 473 human participants, which we named Levels. This dataset is based on the triplet odd-one-out task and included judgments on three different types of triplets: coarse-grained semantic, which require deciding on the odd-one-out in broadly different categories; fine-grained semantic, which involved discerning subtle distinctions within the same category; and class-boundary, which tested for the capacity to identify category boundaries. This dataset allowed us to evaluate whether neural network models respond similarly as humans for the same set of stimuli on various levels of abstraction, and to examine the extent to which the models capture the inherent uncertainty in human judgments, as inferred from response latencies.
Research sample	. We recruited = 508 participants (209 female, 289 male, 3 diverse, = 7 missing demographic information due to revocation of study consent; mean age = 31.75 ± SD: 8.04 years) online via Prolific Academic (https://www.prolific.ac). The eligibility criteria were that participants had to be between 18 and 50 years old, fluent in English, have a normal or corrected-to-normal vision, no colorblindness, and have a minimum approval rating of 95% on Prolific. Participants provided informed consent before starting the experiment. The experiment lasted approximately 45 minutes. Participants were reimbursed with £7.7 for completing the experiment and received an additional bonus payment of £0.77.
Sampling strategy	Participants were sampled randomly from the Prolific participants who met the criteria at the time of recruitment.
Data collection	The experiment was run online using jsPsych v7.3.3 and custom plugins. Participants were asked to provide demographic information, including their age and gender. Thereafter, they viewed written instructions about the task and performed six practice trials (2 trials per triplet level of abstraction). Participants were free to repeat the instructions until they felt confident to perform the experiment. The experiment proper comprised = 330 experiment trials. Each trial started with a fixation cross (1s), followed by the presentation of a triplet (max. 15s). Participants were asked to select the “odd-one-out” using the right, left, or downward facing arrow keys on their keyboard. Responses could be entered between 1-15s after triplet onset, after which the next trial started. Trials in which participants failed to submit a response were rare (M = 0.27% of trials; min = 0.00%, max = 6.06%). The serial order of triplet types (e.g., fine-grained or coarse-grained semantic) and ImageNet classes (e.g., dogs or birds) was counterbalanced across the experiment. We additionally counterbalanced the serial position of trial types across participants using a Latin-Square approach

[LSD; Grant, 1948]. Participants could take short breaks (self-paced for up to 2 minutes) after = 50, 150, and, 200 experiment trials; Experimental trials were interleaved with = 16 catch trials (class border triplets), which were predefined based on low model uncertainty and 100% agreement among participants on these specific triplets during piloting. Catch trial performance was used as an indicator of adequate task engagement (see participant exclusion criteria).

Timing	The participants were recruited between March 7th and April 16th 2024.
Data exclusions	Participants performing below 90% correct on catch trials (= 19, 3 female, 16 male), or failing to respond in the allotted time window (15s) in more than 10 trials (= 9, 4 female, 4 male, 1 diverse) were excluded. Thus, (= 473) participants remained in the dataset (202 female, 269 male, 2 diverse; mean age = 31.82 ± SD = 8.03 years). Of these participants, (= 448) were each tested with a different selection of triplets, while ensuring that each triplet was presented = 5 times across the entire sample of participants (see information on stimuli sampling below). Due to a server glitch during trial assignment, the remaining (= 25) participants shared their exact triplet selection with one other participant in the sample. These (N = 25) participants were excluded from the response times (RT) and uncertainty estimation (see Sec. 2.2.1) to restrict analysis to participants with different sets of triplets.
Non-participation	6 participants did not complete due to technical issues, and 1 terminated participation early. 7 participants revoked consent after the study was completed.
Randomization	The experiment design involved presenting various types of triplet stimuli within-subjects, and different sets and orderings of triplets across subjects. Instead of randomly sampling triplets—which would reproduce dataset biases—we stratified sampling by superclasses. ImageNet classes follow the WordNet hierarchy Deng et al. [2009]; Russakovsky et al. [2015], which includes higher-level classes. For instance, all dog breeds can be summarized as a single dog superclass. To avoid presenting dogs, birds, and other fine-grained classes that are overrepresented in ImageNet more frequently to the participants than other categories, we grouped the ImageNet classes into 717 coarse-grained WordNet superclasses. We uniformly at random sampled images from those 717 superclasses to construct the different kinds of triplets. Note that for all superclasses with more than one class, we uniformly at random chose one subclass and either uniformly at random sampled one image, two images (without replacement), or three images (without replacement) from that subclass, depending on the triplet type. For most superclasses that were comprised by a single subclass only, i.e., a one-to-one-mapping, we could skip the subclass sampling part. Triplet sampling resulted in (= 450) predefined experiment trial sets, of which (= 448) were used for testing. Across these, each triplet was presented within (= 5) different experiment files. This sampling process ensured a balanced distribution of triplets across the sample, and the repetition of each triplet in five different participants allowed for the calculation of an uncertainty distribution for each triplet.

Reporting for specific materials, systems and methods

We require information from authors about some types of materials, experimental systems and methods used in many studies. Here, indicate whether each material, system or method listed is relevant to your study. If you are not sure if a list item applies to your research, read the appropriate section before selecting a response.

Materials & experimental systems

n/a	Involved in the study
☒	☐ Antibodies
☒	☐ Eukaryotic cell lines
☒	☐ Palaeontology and archaeology
☒	☐ Animals and other organisms
☒	☐ Clinical data
☒	☐ Dual use research of concern
☒	☐ Plants

Methods

n/a	Involved in the study
☒	☐ ChIP-seq
☒	☐ Flow cytometry
☒	☐ MRI-based neuroimaging

Plants

Seed stocks	*Report on the source of all seed stocks or other plant material used. If applicable, state the seed stock centre and catalogue number. If plant specimens were collected from the field, describe the collection location, date and sampling procedures.*
Novel plant genotypes	*Describe the methods by which all novel plant genotypes were produced. This includes those generated by transgenic approaches, gene editing, chemical/radiation-based mutagenesis and hybridization. For transgenic lines, describe the transformation method, the number of independent lines analyzed and the generation upon which experiments were performed. For gene-edited lines, describe the editor used, the endogenous sequence targeted for editing, the targeting guide RNA sequence (if applicable) and how the editor was applied.*
Authentication	*Describe any authentication procedures for each seed stock used or novel genotype generated. Describe any experiments used to assess the effect of a mutation and, where applicable, how potential secondary effects (e.g. second site T-DNA insertions, mosiacism, off-target gene editing) were examined.*
